# Economic and health consequence frames affect COVID-19 vaccine incentive attitudes in Germany– a survey based framing experiment

**DOI:** 10.1186/s12889-025-23279-x

**Published:** 2025-06-04

**Authors:** Sebastian Jäckle, James K. Timmis

**Affiliations:** 1 Department of Political Science, University of Freiburg, 79085 Freiburg, Germany; 2 Athena Institute for Research on Innovation and Communication in Health and Life Sciences, Vrije Universiteit Amsterdam, Amsterdam, 1081 HV The Netherlands

**Keywords:** Vaccine uptake, Framing, COVID-19, SARS-CoV-2, Immunization incentives

## Abstract

**Background:**

SARS-CoV-2 vaccines have significantly reduced human and economic losses. Nevertheless, vaccine hesitancy remains a major issue in many countries, including Germany. Recent studies have shown that public health framing and incentives can boost immunization rates. However, available evidence is fragmented and inconclusive regarding the effectiveness of different framing messages, types of incentives, and the size of financial incentives across different populations.

**Methods:**

This randomized, controlled survey experiment elicited the attitudes of 6,685 Germans towards 4 financial/non-financial SARS-CoV-2 immunization incentives (food voucher, football tickets, participation in lottery, immediate monetary compensation), and tested whether framing (individual/collective, health/economic consequences) affected said attitudes. We assigned participants to five study arms (control: no frame; experiment: 1 of 4 frames) and measured attitudes towards immunization incentives, and the amount of monetary compensation deemed appropriate, should such an incentive be considered.

**Results:**

While > 75% of our sample considered all 4 incentives to be not meaningful, all frames increased favorable views towards the financial incentives lottery/money and the average amount deemed acceptable for immediate monetary compensation. Interaction models showed that all frames have similar effects across core subgroups, e.g. age-cohorts, gender, vaccine doses.

**Conclusions:**

Across a sample of 6,685 Germans, we show that 4 different frames detailing the potential individual/collective consequences of COVID-19 have very similar effects on attitudes towards monetary incentives for SARS-CoV-2 immunization. Our results suggest that the existence of frames rather than specific narratives is key to increasing favorable views towards immunization incentives.

**Clinical trial number:**

Not applicable.

**Supplementary Information:**

The online version contains supplementary material available at 10.1186/s12889-025-23279-x.

## Introduction

Besides the provision of clean water and basic sanitation, immunization is considered the most important and (cost-)effective public health tool for preventing the spread of communicable diseases, and reducing pertinent morbidity and mortality [1]. Vaccines have saved countless lives and driven the demographic dividend and thus economic prosperity and welfare initially witnessed across contemporary high-income countries (HICs), and more recently across middle and low-income countries (LMICs) [2]. According to the WHO, immunization annually prevents 3.5-5 million deaths that would have otherwise occurred from infectious diseases [3].

The COVID-19 pandemic caused by the SARS-CoV-2 strain (and multiple mutations thereof), directly or indirectly resulted in an estimated 15 million deaths worldwide in 2020 and 2021 [4], and the total economic costs have been projected to reach a cumulative 13.8 trillion USD by the end of 2024 [5]. By the same token, a recent study estimated that, in the year between 12.2020 and 12.2021, vaccines against SARS-CoV-2 prevented up to 20 million deaths globally that would have otherwise resulted from COVID-19 [6]. Studies have also shown that SARS-CoV-2 vaccines have prevented economic losses amounting to hundreds of billions of dollars worldwide [7, 8]. Despite these highly favorable statistics, and public health authorities communicating the compelling evidence demonstrating the excellent safety (and cost-effectiveness) profiles, in particular of mRNA-technology based SARS-CoV-2 vaccines, vaccine hesitancy and refusal (VacHes) remain major issues.

While efforts to alleviate concerns and persuade individuals to have vaccines are for the most part effective, the repercussions of the substantial increase of VacHes over the past decade (frequently initially based on Mumps, Measles and Rubella vaccines [9]), led the World Health Organization to just recently– 2019, i.e. before the onset of the COVID-19 pandemic– designate VacHes one of the top *Ten threats to global health* [10]. VacHes is a highly complex and multifaceted phenomenon. Previous studies have shown that, besides better explored determinants of VacHes– such as concerns over vaccine effectiveness, potential side-effects, and distrust towards government agencies and ‘Big Pharma’– socio-political and psychosocial attitudes, as well as social group norms (and pertinent social capital) can play important roles in individual decisions on immunization [11–14]. This is important as there exists evidence that socioeconomic factors, and attitudes associated with VacHes, can act as conduits for VacHes spill-over effects on other jurisdictions and countries– especially due to organized (foreign) anti-immunization activities, more recently predominantly via social media [15, 16].

A recent study found that ~ 22% of the German population was SARS-CoV-2 vaccine hesitant in 2021. This compares with a global average of ~ 20%. Among the surveyed European countries, only Poland had a larger proportion (38%) of vaccine hesitant individuals [17]. However, VacHes is not uniformly strong across different regions in Germany. On average, individuals residing in Eastern German, and to a slightly lesser degree in Southern German, states tend to be substantially more hesitant than their peers in Northern and Northwestern states. Germany is a particularly important case for investigation, as anti-vaccination groups have been central in driving the global third generation anti-immunization wave [18]. Therefore, it is essential to better understand which measures improve uptake in Germany.

When faced by suboptimal SARS-CoV-2 immunization rates, and despite sufficient supply and access to pertinent vaccines beginning of 2022, a range of European countries had been or were considering immunization mandates [19]. However, mandates are an *ultima ratio* that can provoke severe resistance in those who are hesitant and even more so those who are refusing based on a range of, often refutable, ethical arguments [20]. In any case, evidence regarding the effectiveness of mandates is mixed. While some studies find that mandates lead to intended increases in vaccine uptake, others indicate that vaccine hesitant individuals can become vaccine refusers due to *inter alia* the perceived wrongful interference of state actors in matters considered an individual choice. German public health authorities and politicians debated mandates for specific cohorts, such as healthcare professionals and vulnerable groups, but also for the general population [21]. The German parliament ultimately decided to introduce a SARS-CoV-2 vaccine mandate for professionals working in health and social care institutions in December 2021, which was met by fierce protests across, but especially in East, Germany [22, 23].

It is widely acknowledged that vaccine promotion and messaging is an important component of immunization campaigns to inform immunization decisions. In particular directive, succinct and factual information (e.g. statistics) about COVID-19 and SARS-CoV-2 vaccines were considered highly persuasive in a recent US study– personal immunization reminders (i.e. behavioral nudging) and dose reservations have been shown to increase uptake, just as emphasizing that a particular vaccine in question is free [24–27]. A recent study in the US found that reminders meaningfully increased booster uptake [28]. Regarding appeals-based messages, public health messaging strategies frequently utilize cues that can be grouped into individual or community-focused messages based on factual accounts of benefit and risk but also emotional appeals, such as protection of others and loved ones [29]. While the associations between immunization intentions/decisions and frames based on the risk of personal economic and social function losses or the economic costs to society resulting from serious COVID-19 have been explored, the evidence base is inconclusive. Importantly, while studies have been published regarding the impact of vaccination on Long COVID, there exists paucity of evidence regarding the effectiveness of public health messages on vaccine uptake framed around the risks of life-changing sequelae following COVID-19. This is surprising as Long COVID has become a major long-term and impactful reality for patients whose lives have been upended in matters that are central to personal economic and social functioning– according to McNabb et al., “1) occupational and financial; 2) healthcare-related; and 3) social and emotional support” are key dimensions affecting the quality of life for those with Long COVID [30]. Likewise aggregate economic costs resulting from Long COVID due to productivity losses, and treatment and rehabilitation of Long COVID patients are significant– in Germany alone these were estimated at a total of 5.7 billion EUR in 2021 and 9.3 billion in 2022 [31]. Therefore, more evidence on the persuasiveness of Long COVID risk based public health messages on vaccine decisions is needed.

Various studies indicate that incentives can be (a) significantly more effective than public health messaging alone and (b) much less disruptive than mandates to increase uptake of vaccines [32, 33]. During the SARS-CoV-2 pandemic, an unprecedented number and range of incentives (in scope but also in monetary value) were tested. Examples include.

i. small non-financial rewards (e.g. free grilled sausage),
ii. direct financial payout or vouchers of, in relative terms, lower value (e.g. 10–250 USD), or.
iii. participation in lotteries, in some cases with substantial rewards, such as, educational scholarships or cash payouts– 3 × 50.000 USD in Philadelphia, 1.000.000 AUD in Australia and 5 × 1.000.000 USD in Ohio [34, 35].

However, the evidence base regarding the effectiveness of different types of monetary incentives is fragmented. Most reports included in a recent systematic review of studies on SARS-CoV-2 vaccine incentive effectiveness originated in the US (*n* = 18), while three were from Germany, and one each from Australia, Poland and the UK [36]. The review does not only show the geographic concentration of evidence originating from the US but also that outcome measures are often not comparable across studies: a little over half of studies included in the review measured *de facto vaccine uptake* (*n* = 14) while the others measured *immunization intention* (*n* = 9) or *self-reported immunization status* (*n* = 1). Regardless, the review came to the conclusion that, overall, “high financial incentives and the Vax-a-million lottery [in Australia] are attributed to a higher vaccination rate, while the low amount of financial incentives, other lotteries, and persuasive messages have small or non-significant effects” [36]. Although evidence indeed suggests that the 1.000.000 AUD incentive in Australia positively contributed to reaching a 80% vaccination rate [35], results for the 5 × 1.000.000 USD incentive in Ohio were mixed [37–39]. Another systematic review of 26 studies on different types of incentives came to somewhat different, and overall sobering results. While it detected no evidence at all of a positive effect on vaccination for lotteries and non-cash incentives, it found that direct cash transfers increased coverage and immunization intentions, however, the effect was not particularly strong [40].

Regarding small direct payments, the evidence base is particularly mixed. For Germany, a factorial survey experiment came to the conclusion that a 50 EUR incentive increases the willingness-to-accept a vaccine among the undecided by about 5% points– in combination with two other incentives (providing freedoms, i.e. regaining access to public events, such as concerts, and spaces, for instance restaurants, and vaccination at local doctors) even up to 13% points. Similarly, a Swedish study found that a modest monetary incentive of 24 USD was sufficient to increase the de facto SARS-CoV-2 immunization rate by 4.2% among ~ 9000 respondents. By the same token, other studies from the US either found no positive effect of small incentives (10–100 USD) or found that effects are strongest for larger amounts (1000 USD) [26, 41, 42]. A recent evidence brief came to the conclusion that, indeed, there exists a knowledge gap regarding the incentive threshold for vaccination [43]. Evidence demonstrating what (non-)hesitant individuals in Germany consider an adequate financial incentive for hesitant individuals to have a vaccine is lacking. This is all the more important, as financial incentives covered by public funds are only sustainable if they are supported (over-time) by the majority of the population.

There exists also evidence that disincentives (losses), such as the prospect of job loss or increased insurance premiums, are more effective at increasing uptake than most types of monetary rewards (gains). Nevertheless, while lotteries and lower value gift cards were (once again) not persuasive in a recent US study, higher value gift cards were basically as effective as the loss-based disincentives [44]. Brewer et al. recently argued that three criteria are central in improving the likelihood that financial incentives have the intended effects: monetary rewards must be (i) *certain*, (ii) *immediate* and (iii) *valued* by recipients [45]. This once again indicates that lotteries might not be dependable public health tools, a finding echoed by Campos-Mercade et al. [46]. In a more recent working paper, Campos-Mercade et al. also found that guaranteed small monetary incentives (20 EUR) increased booster uptake by up to 13% points among a Swedish general population sample [47]. In any case, the overall variation in conclusions of previous studies in various jurisdictions on the effectiveness of different types of financial rewards (and value) as incentives for immunization renders generalizability highly challenging. While German politicians debated (nation-wide) financial rewards for vaccination in 2021 [48], these were ultimately not implemented. However, non-financial rewards were offered at the regional level by municipalities or public-private-partnerships, such as rewarding SARS-CoV-2 immunization with a free grilled sausage (which was adopted by many German cities) or tickets to an upcoming football (soccer) match (in Lower Saxony)– both of which were reportedly successful– yet scientific validation studies are lacking [49].

We have previously shown that, in particular in Germany, and besides sociopolitical viewpoints, attitudes towards esoteric worldviews and complementary and alternative medicine (CAM) play an important role in VacHes [11]. Moreover, we found that certain drivers of immunization decisions (voluntary considerations versus external pressures) are associated with specific attitudes towards vaccination [12]. While identifying attitudes towards vaccination and VacHes narratives in Germany is relevant from a diagnostic standpoint, the elicitation of attitudes towards potential remedies, i.e. mechanisms that potentially increase uptake in Germany, is particularly important. This is because, as alluded to above, German anti-vaccination groups are central in fuelling the contemporary (global) increase in vaccine hesitancy [18]. Eliciting the views of Germans towards immunization incentives does not only have important implications for addressing VacHes in Germany but also for VacHes ‘exported’ to other jurisdictions.

To the best of our knowledge, evidence regarding the association between Long COVID messaging and preferences of the general population on providing immunization incentives does not exist. Moreover, while some of the framing studies mentioned above used concrete statistics of overall national economic costs due to the COVID-19 pandemic, we consider the average hospitalization cost of COVID-19 cases to be a more direct and therefore readily relatable metric: in Germany, the average hospitalization cost per case for COVID-19 patients without mechanical ventilation (MV) were 5,800 EUR, with MV 34,200 EUR, and for those that required extracorporeal membrane oxygenation 92,000 EUR [50]. It is also not clear if information frames regarding the costs directly associated with hospitalization due to severe COVID-19 impacts attitudes towards incentives for vaccine hesitant individuals. We also consider the associations that we previously identified between specific immunization drivers and sociopolitical/psychosocial attitude patterns.

Our study is guided by the following research question: What are the attitudes of Germans towards different (non-)financial SARS-CoV-2 immunization incentives, and do different framing messages regarding (1) health consequences as well as personal economic and social function losses due to Long COVID and (2) collective economic losses associated with hospitalization due to severe COVID-19 have an impact on shaping their attitudes towards immunization incentives? Furthermore, the study explores to what extent certain frames show better/worse results with certain groups of persons.

## Methods

### Analytical strategy

This analysis is based on a randomized, controlled framing experiment included in an online survey on political, social and health questions that was conducted in Germany. During the survey participants were randomly assigned to one of five groups. Four groups were presented with one of four frames, i.e. short newspaper texts on different consequences of Long COVID and not being vaccinated (see Supplementary Note 1 for the exact wording). The fifth group (control) was not presented with a frame. Table 1 gives an overview of the overall research design and Supplementary Table 7 lists all questions and answer options from the survey and their exact wording as well as their position within the survey. Most of the controls used in the regression models, except for the socio-demographic variables, were asked before the frames were presented to minimize the risk of conditioning on post-treatment variables.

**Table 1:** Overview of study design

Framing experiment	Survey participants were presented with one of the following:				
	Control condition No article	Long COVID Frame 1 “Pandemic: Recovered but not healthy: Long COVID still in focus” - General example - Risk of range of mild to life-changing sequelae - Vaccination reduces risk of Long COVID	Long COVID Frame 2 “Discontinued health insurance payments - Long COVID patients face financial crash” - Specific, emotional, personal example - Risk of poverty due to life-changing sequelae	Economic Frame 1 “The cost of treating unvaccinated COVID-19 patients” - High costs of COVID-19 hospitalization - Recommendation of compulsory immunization and out-of-pocket cost sharing (unvaccinated)	Economic Frame 2 “Health insurance companies calculate: COVID-19 patients in intensive care cost tens of thousands of euros” - Serious cases cost more than treatment of cancer patients and injuries caused by accidents
	All participants were then asked to answer				
Dependent variable	Outcome I: How would you rate the following incentives to encourage people to have a SARS-CoV-2 vaccination? *Non-financial incentives* • Free ticket for football match (henceforth: *football ticket*) • Free grilled sausage (henceforth: *sausage*) *Financial incentives* • Vaccination lottery (chance to win big) (henceforth: *lottery*) • Money for vaccination (henceforth: *money*) Level: • 0 = Not at all meaningful • 0.25 = Rather not meaningful • 0.5 = Neutral • 0.75 = Rather meaningful • 1.0 = Very meaningful			Outcome II: Provided there was a financial incentive for SARS-CoV-2 vaccination, how much money would you consider appropriate? Level: • Not at all, I think the concept of financial incentives is wrong. • Up to 19.99 Euro • 20 to 49.99 Euro • 50 to 199.99 Euro • 200 to 499.99 Euro • 500 Euro or more	
Controls	• SARS-COV-2 vaccination status (n of doses received; 0–4) & perception of situation • Attitudes towards esoteric and medical belief systems & religion • Political and psychosocial factors • Socio-demographic factors				
Statistical analysis					
Methods	• Descriptive statistics (by vaccination status) • Comparison of framing group mean attitudes towards incentives (Dunnett’s test) • Bivariate OLS regression (& Ordinal Logit as crosscheck) • OLS regression including controls (& Ordinal Logit as crosscheck) • Interaction models (frame x control variable)				

### Study population

**Fig. 1:**
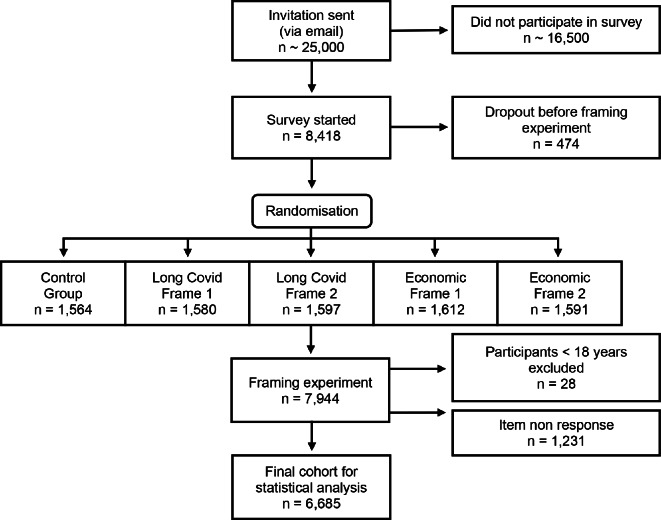
Participant flow chart. Annotation: Online survey conducted in Germany between June 30th and July 17th, 2022. With 5.6% dropout was low. Item non-response was between 0.35% and 0.55% for the outcome variables and between 0.11% and 4.57% for the controls (see Supplementary Table 8). The final sample reduced via listwise deletion shows no compositional differences to the original raw data (see Supplementary Table 9)

The survey was part of a long-time panel study at the University of Freiburg, Germany (Politikpanel Deutschland) that has surveyed several tens of thousands of people since 2017. The panel consists of a wide and heterogeneous group of ~ 25,000 individuals who were invited to participate in our survey on political and social questions. In order to increase the response rate, we used an incentive: participants had the chance to win one of 25 coupons worth 20 Euros (to be used at Amazon, a German Bookstore, an online organic supermarket, or donated to UNICEF). We also sent two reminders. Since each invitation link was only valid for one participant, multiple participation was not possible. The field time was June 30– July 17, 2022. It was administered via the online survey software Unipark. A week before the start of the survey, the questionnaire was pretested once by *n* = 12 students who were not aware of the framing experiment and the purpose of the survey. The pretest resulted in minor changes in the wording of the survey questions. 8,418 individuals started the survey, providing informed consent at the very beginning of the online survey (in line with the EU General Data Protection Regulation)– 474 (5.6%) dropped out before the framing experiment (see Fig. 1). 1,564 participants were assigned to the control group, and between 1,580 and 1,612 to one of the four framing groups. Participants younger than 18 (*n* = 28) were excluded from the analysis. Item non-response was comparatively low (between less than 0.6% for all the dependent variables and a maximum of 4.6% for the control variable “perception of social division”, see Supplementary Table 8). A total of 1,231 cases were deleted due to missing values in one of the variables, so that the final cohort for the statistical analysis comprises 6,685 cases. Importantly, a comparison between the full original data and the latter dataset that we used for the regression analyses shows virtually no differences in composition (Supplementary Table 9). Item non-response thus does not introduce substantial systematic bias into the dataset which in turn means that a complete cases regression analysis (listwise deletion) is feasible and alternative imputation approaches are unnecessary. As is the case with all self-selecting online surveys, our sample is not de facto representative of the general public. Nevertheless, due to the different recruitment channels, the distribution of participants in terms of socio-demographic characteristics is relatively close to that of the German population as a whole, as a detailed comparison between the Politikpanel Deutschland sample and the German population in terms of gender, age, education and party affiliation demonstrates [51]. Because of this, and since we are interested in individual level effects (of frames) and not in overall population characteristics– which means that representativeness is not crucial– we use unweighted data in the statistical analyses. The survey data is stored on a secure and GDPR-conform Unipark server. Any potentially identifying information (particularly email addresses) were dropped from the dataset before the start of the analysis, so that it is impossible to subsequently identify individual participants.

### The framing experiment

All four frames were real news articles from online news outlets in Germany published between December 2021 and June 2022. They were each shortened to 114–252 words (Supplementary Note 1). The articles were chosen based on the idea that providing information on different consequences of (non)vaccination may influence the attitudes towards vaccination incentives. Two frames focused on Long COVID and two on the economic consequences/high costs of COVID-19 hospitalization: Long COVID frame 1 explained that even after mild infections a significant number of patients develops long-term complications with serious symptoms (fatigue, brain-fog, shortness of breath). It also explicitly points out that vaccination reduces the risk of Long COVID. Long COVID frame 2 uses the example of a 34-year-old woman with severe Long COVID symptoms. The article shows, in an emotional fashion, that Long COVID is not only a health issue, but that patients often also have to deal with social functioning and economic decline. Economic frame 1 describes the high costs for a COVID-19 intensive care patient (on average 10,200 euros), which are borne by the population. It also presents the demand of a medical association to introduce both a general vaccination mandate and a cost-sharing scheme for hospital costs for admitted unvaccinated individuals. Economic frame 2 also presents the high costs of ventilated inpatient treatment of COVID-19, also detailing that the treatment of these patients is higher than for cancer patients.

### Measures

After the presentation of the frames, all participants were asked about their attitudes towards four types of incentives for a SARS-CoV-2 vaccination that had either been applied locally in Germany or at least been discussed as options within German politics: free ticket for a football match, free grilled sausage, vaccination lottery and direct monetary compensation. The answers on a five-point Likert scale were used as Outcome (I) Participants were additionally asked how much money they would consider appropriate for a direct cash incentive– provided there was such a monetary incentive (Levels: Not at all, I think the concept of financial incentives is wrong; up to 19,99 EUR; 20 to 49,99 EUR; 50 to 199,99 EUR; 200 to 499,99 EUR; 500 EUR or more). For the statistical analysis the answers from this 6-point scale were recoded to the means of the categories (e.g. “20 to 50 Euro” ➔ 35 Euro) except for the first and the last categories (“nothing” and “500 and more Euro”) which were coded as 0 and 500. The resulting metric scale was applied as Outcome (II) Since the five groups were randomized, any differences found in the outcomes between the groups should be caused by the different frames. Yet, since it is plausible that other factors are also relevant in understanding the within-group variation and thus the overall variation in both outcomes, we additionally asked for a range of controls, e.g. the number of SARS-CoV-2 doses received, attitudes towards Waldorf schools, and complementary and alternative medicine, political ideology and voting intention, and BIG-5 personality traits (Supplementary Table 7 lists all controls). For additional information on the operationalization of controls and the reliability and validity of the two applied survey instruments (solidarity scale and Big Five Inventory BFI 10) see [12].

### Characteristics of participants

Supplementary Fig. 6 compares the survey data (reduced via listwise deletion to the 6,685 cases which are used in the later statistical analyses) with official statistics from the Federal Statistical Office of Germany on gender, age, education and place of residence by state (Bundesland). This figure also presents a comparison of our sample with respect to party affiliation to the mean of four representative opinion polls that were conducted in Germany between June 28th and July 6th, 2022 (Forschungsgruppe Wahlen, FORSA, Infratest Dimap, YouGov), which use exactly the same question (Sunday Question) as we do in our survey to query voting intention. Males, higher educated individuals, those living in the city state of Bremen and the south-western state of Baden-Wuerttemberg and individuals who intend to vote for the Green party are overrepresented.

### Statistical analysis

Data were analyzed using Stata 15.1. We first described both outcome variables using stacked bar plots comparing the groups of vaccinated and unvaccinated participants. For Outcome II we additionally calculated the mean amount of financial compensation deemed acceptable as an incentive stratified by the different answers given to the question how meaningful financial compensation is considered overall. In order to test the effects of the frames, we present the means of Outcome I and II for the four framing groups in comparison to the control group. We additionally calculated Dunnett’s tests for the multiple comparison of the four framing group means with the mean of the reference category (control group) [52]. In order to investigate the associations of the four frames with the attitudes towards the incentives in more detail we started by estimating bivariate OLS regressions on both Outcomes. As crosscheck we estimated the regressions using ordinal logistic regression models. In the next step we test whether the effects found in the bivariate analyses prevail when the controls discussed above are included in the model. The effects of the frames are presented using plots based on these multiple OLS models. These figures show the predicted meaningfulness of each incentive (Outcome I) as well as the money deemed acceptable as compensation for vaccination (Outcome II) by framing group and the b-coefficients from the multiple OLS model for Outcome I. All these figures include 95% CI as uncertainty estimates. We also estimated additional models to control for the following additional variables: self-perceived importance of six specific factors for vaccination (among those who had received at least one dose of SARS-COV-2 vaccination), self-perceived knowledge about SARS-COV-2 vaccination, and public/private health insurance [12]. Finally, we estimated interaction models testing to what extent specific frames are associated with specific positive or negative attitudes towards certain incentives.

### Study registration

The framing experiment was not pre-registered. This was neither required under local guidelines nor did it seem appropriate, given the, at least partially, exploratory nature of the research question.

### Data availability

The datasets generated and/or analyzed and underlying code for this study are available in the Harvard Dataverse repository, 10.7910/DVN/5RW50M.

## Results

### Sample

Our online survey was conducted in Germany from June 30– July 17, 2022. 8,418 individuals started the survey. 6,685 participants answered all of our questions. Figure 1 shows an overview of the participant flow– further details on participants, missing data and representativeness can be found in Methods.

Overall, our descriptive analysis shows that in July 2022 merely a minority of 10–25% among our German sample held a positive view towards vaccination incentives with *sausage*, *football tickets* and *money* being less popular than *lottery*. Furthermore, attitudes towards the incentives, as well as the amount of money– if financial compensation was deemed appropriate at all– do not depend on the gender of the respondents.

**Fig. 2:**
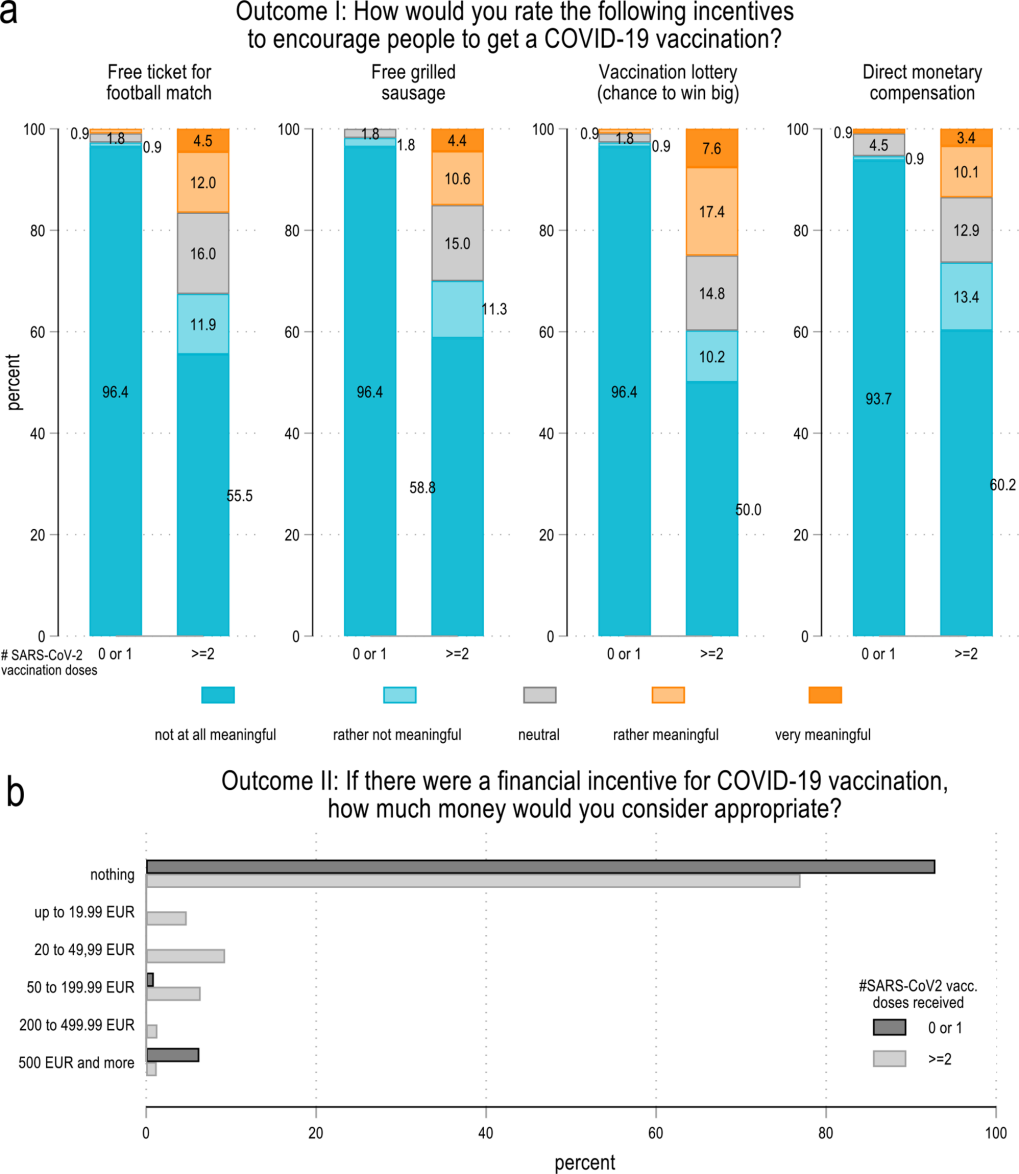
Baseline (= control group) descriptive statistics of dependent variables by vaccination status. Annotation: Panel **a** shows percentages of survey participants’ attitudes towards four COVID-19 vaccination incentives by number of vaccination doses received (0/1: not/not fully vaccinated; ≥2: fully vaccinated). Panel **b** shows percentages of survey participants’ views on the appropriate level of a potential direct monetary compensation for COVID-19 vaccination by number of vaccination doses received (0/1: not/not fully vaccinated; ≥2: fully vaccinated). *N* = 1,329 (control group only). Survey data from Politikpanel Deutschland, 06/30 − 07/17/2022 (https://www.politikpanel.uni-freiburg.de/)

As a baseline, Fig. 2 shows the attitudes of those survey participants who were not presented with any frame (= control group) towards the four incentives (Outcome I), as well as how much money they consider appropriate as a financial incentive for vaccination (Outcome II) by their vaccination status. The majority of them (63.4–75.4%) considers all four incentives to be rather not or not meaningful. Among the four alternatives, the incentive *lottery* is most likely to be considered meaningful– but even in this case, less than 1/4 of the respondents consider such a lottery to be rather or very meaningful.

The data show major differences between fully vaccinated (≥2 doses) and not or not fully vaccinated (≤1 dose) control group respondents. Those who are fully vaccinated are less negative regarding all four incentives compared to not/not fully vaccinated respondents. The non-financial incentives (*football tickets* and *sausage*) as well as the financial incentive *lottery* are seen very negatively by not/not fully vaccinated respondents, with more than 96% rating these incentives as not meaningful at all. While the incentive *lottery* is considered most useful by the vaccinated (25.0% rate it as rather or very meaningful), the not/not fully vaccinated consider *money* to be the most compelling incentive (5.4% are at least neutral in that regard). With respect to the question how much money is considered appropriate for vaccination, 77.0% of the fully vaccinated and 92.9% of the not/not fully vaccinated said “nothing”. Interestingly, while only 1.2% of the fully vaccinated consider ≥500 EUR as appropriate, more than five times as many not/not fully vaccinated individuals (6.3%) indicated that they view ≥500 EUR as appropriate. Leaving the large majority of those aside who consider money as a vaccination incentive completely inappropriate, on average vaccinated respondents would consider payments of 97.80 EUR to unvaccinated individuals to be appropriate to encourage them to have the vaccine; not/not fully vaccinated respondents would pay 453.13 EUR.^1^ Gender plays no major role in the baseline (= control group) attitudes towards the four incentives (Supplementary Fig. 1).

**Fig. 3:**
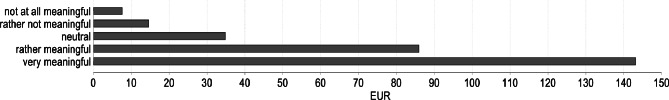
Baseline (= control group) average money deemed acceptable as compensation for vaccination by the attitude towards money as an incentive for vaccination in general. Annotation: *N* = 1,329 (control group only). Survey data from Politikpanel Deutschland, 06/30 − 07/17/2022 (https://www.politikpanel.uni-freiburg.de/)

Figure 3 combines Outcome I and II and stratifies the amount of money participants deem appropriate with respect to how meaningful they generally rate money as an incentive for vaccination. The illustration shows the expected variation– those who regard money as not being a meaningful incentive on average consider a compensation of less than 8 EUR to be appropriate while those who indicate that money is a meaningful incentive would on average view more than 143 EUR to be adequate.

In a next step, we explored if, and if so to what extent, attitudes towards vaccination incentives differ if respondents are presented with different pieces of information (frames) detailing the potential consequences of severe COVID-19 or Long COVID– the frames covered inter alia sequelae and social function losses, as well as individual and/or collective economic consequences.

**Fig. 4:**
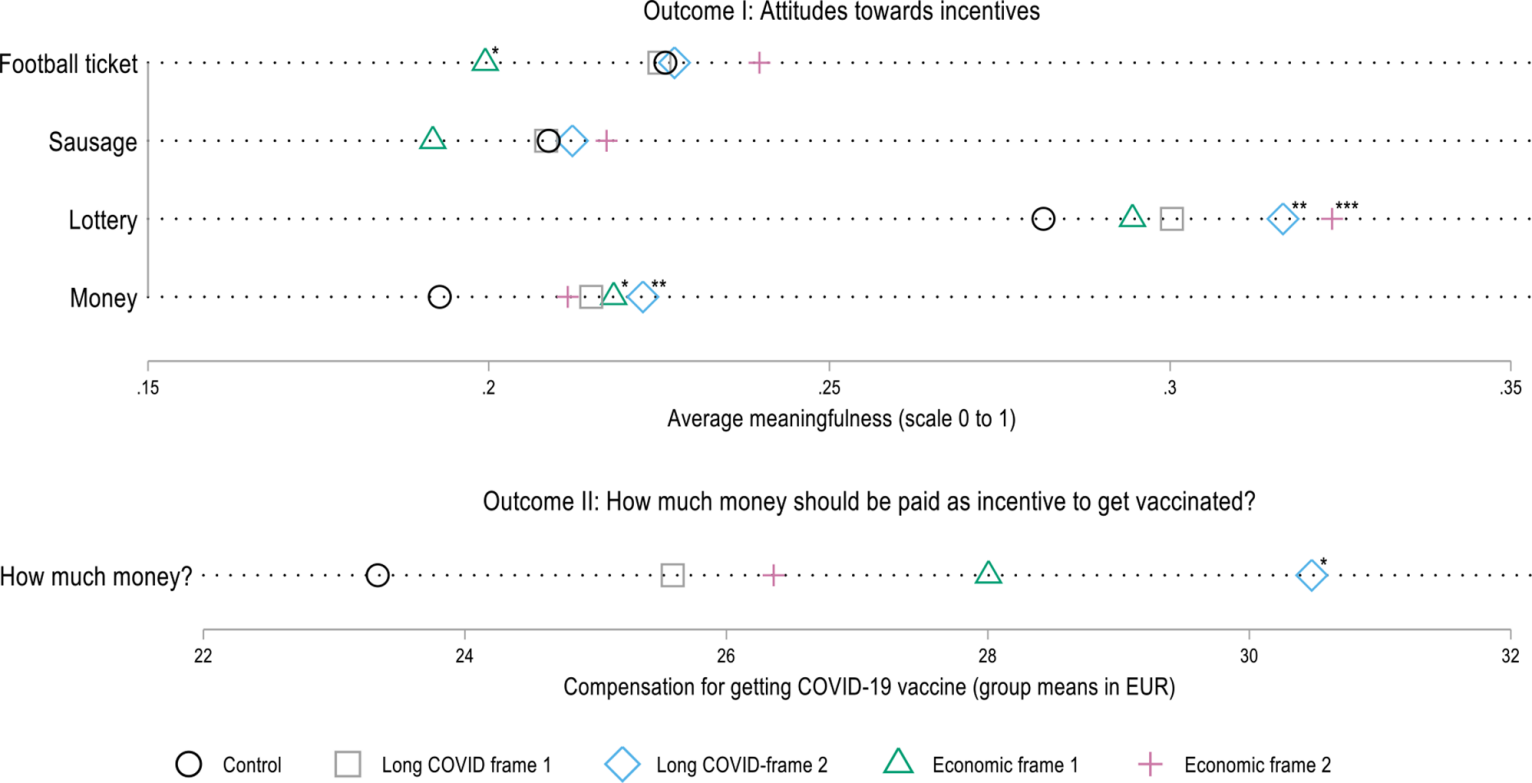
Differences in the means of outcome variables between the framing groups (+ results of Dunnett’s test). Annotation: *N* = 6,685. Survey data from Politikpanel Deutschland, 06/30 − 07/17/2022 (https://www.politikpanel.uni-freiburg.de/). Stars indicate significant differences to control group calculated via Dunnett’s test. * *p* < 0.1; ** *p* < 0.05; *** *p* < 0.01

Figure 4 presents respondents’ average attitudes towards the four incentive strategies (Outcome I) and the mean money they would deem appropriate for a vaccination incentive (Outcome II) grouped by the experimental frame they received. First of all, our results once again show for Outcome I that all four incentives tend to be not meaningful to respondents (average meaningfulness scale scores range from 0.19 to 0.32; scale 0–1), with lottery being viewed least negatively. The major insight from this figure is, however, that certain differences between the five groups (control group and four different framing groups) are apparent (Dunnett’s tests). While the specific frame with which the respondents are presented does not make a major difference to how meaningful they view a *football ticket* or *sausage* as an incentive, larger differences can be observed for *lottery* and *money*. For these two incentives, the averages of all framing groups are higher, at least by trend, than the average of the control group. This means that individuals presented with any of the four frames detailing consequences of severe COVID-19 or Long COVID view the incentives *lottery* and *money* to be more meaningful than the control group. The largest differences in the average meaningfulness can be observed for the *lottery* incentive between the control group and those participants who were presented with Economic frame 2 (*high costs of COVID-19 hospitalization: serious cases requiring mechanical ventilation on average cost more than treatment of cancer patients and injuries caused by accidents*) or Long COVID frame 2 (*specific*,* emotional example: Risk of life-changing sequelae leading to poverty and social exclusion*).

Overall, the results for Outcome II point in the same direction. Participants who are presented with one of the four frames are on average, at least by trend, willing to provide somewhat larger financial incentives. Yet, these results do not reach the 95% level of confidence. Among the entire sample of all survey participants, those in the control group indicate that they consider an average of 23.33 EUR to be appropriate, while those presented with Long COVID frame 2 regard 30.48 EUR to be an appropriate monetary incentive. For those respondents who did not answer “nothing” to the question on how much money they consider to be appropriate, the magnitude and differences between the control group’s mean (107.67 EUR) and the mean of the groups with Long COVID frame 2 and Economic frame 1 (127.67 EUR/118.80 EUR) are significantly larger.

When pooling either (i) the two Long COVID frames & the two Economic frames, or (ii) all four frames and comparing the means of these pooled frames with the mean of the control group, significant differences for the incentive lottery (all at *p* < 0.05) and for the incentive money (*p* < 0.05 for the pooled Long COVID frames, *p* < 0.1 for the pooled Economic frames, and *p* < 0.01 for the all four frames pooled together) can be observed. For Outcome II, the mean amount of money which respondents consider appropriate as an incentive to get vaccinated differs significantly (*p* < 0.1) from the control groups’ mean (see Supplementary Table 1).

Bivariate OLS regressions on Outcome I show that, despite a negative association between Economic frame 1 and the incentive *football tickets* (*p* < 0.05), none of the other frames are statistically significantly associated with attitudes towards the two non-financial incentives *sausage* and *football* (Supplementary Fig. 2). Compared to the control group, attitudes towards the incentive *money* are significantly (*p* < 0.05) more positive in the Long COVID frame 2 and Economic frame 1 groups. The attitudes of those participants who received the Long COVID frame 2 or Economic frame 2 are significantly (*p* < 0.05) more positive towards the incentive *lottery*. Long COVID frame 2 also shows the strongest association with the amount of money considered appropriate by respondents (= Outcome II). The participants in this group would on average consider 7.14 EUR more to be adequate than those in the control group. Yet, overall, the strongest correlation can be observed between Economic frame 2 and *lottery*. The group that received this frame gives *lottery* an average meaningfulness score of 0.32 which is 0.04 points (= 15.0%) higher than the score in the control group (0.28). These results do not change substantially when the models are estimated using ordinal logistic regression.

**Fig. 5:**
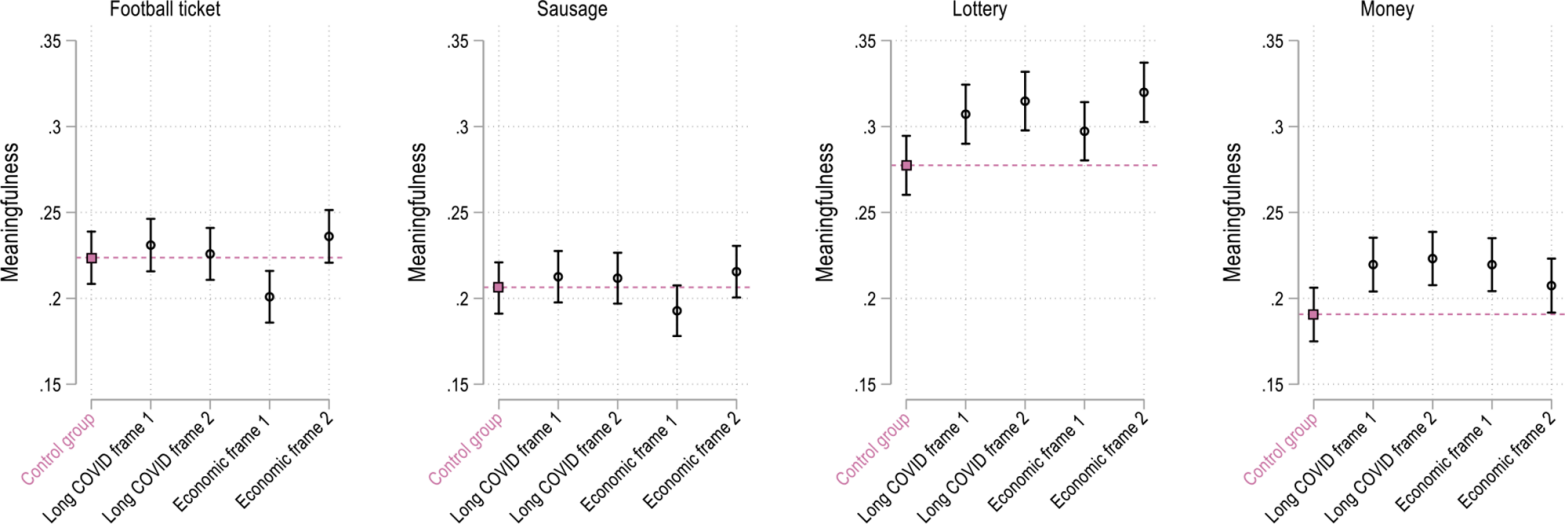
Predicted meaningfulness of vaccination incentives based on multiple OLS model. Annotation: *N* = 6,685. Survey data from Politikpanel Deutschland, 06/30 − 07/17/2022 (https://www.politikpanel.uni-freiburg.de/). Predictions + 95% CI based on multiple OLS models including a range of controls. See Supplementary Table 2 for the full models. Again, as is the case for the bivariate models, the results for the frames do not change substantively when we estimate ordinal logit models instead of OLS models (see Supplementary Fig. 3)

Figure 5 presents the meaningfulness scores for the four framing groups based on predictions from OLS models controlling for the full set of controls mentioned in Table 1. For the two incentives *money* and *lottery* the predicted meaningfulness scores across all four framing groups are significantly higher (*p* < 0.05) than in the control group. For the incentives *football tickets* and *sausage* such positive framing effects were not detected. These results do not change when the four frames are pooled (see Supplementary Fig. 4).

**Fig. 6:**
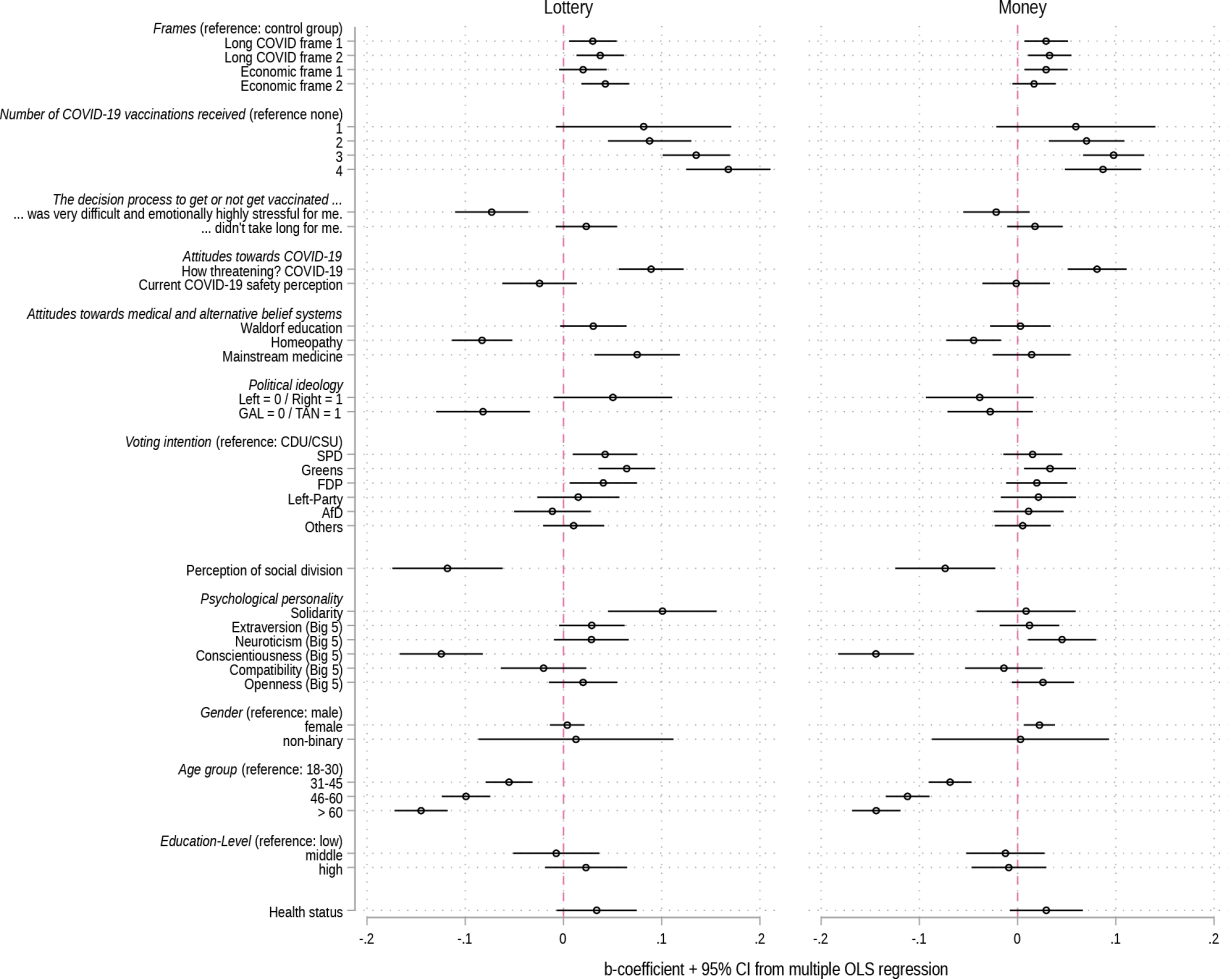
OLS regression explaining attitudes towards money and lottery as vaccination incentives (b-coefficients + 95% CI). Annotation: *N* = 6,685. Survey data from Politikpanel Deutschland, 06/30 − 07/17/2022 (https://www.politikpanel.uni-freiburg.de/). The models additionally control for state, religion and household income. Coefficients for these three variables not presented due to space restrictions. See Supplementary Table 2 for the full models including also models for the other two incentives football and sausage

Figure 6 presents the b-coefficients for all of the variables included in the multiple OLS models, showing the attitudes towards the two incentives for which the frames work best: *money* and *lottery*.^2^ Compared to the control group, the estimates of the coefficients in all experimental groups are positive. This means that participants who received one of the frames are more positive towards *money* and *lottery* as immunization incentives compared to those who were not presented with a frame. The strongest association can be found for Economic frame 2 and attitudes towards *lottery*. Those who received this frame view *lottery* 0.043 points (scale 0–1, *p* < 0.001) more positively than the control group. Regarding controls, the regression model demonstrates that, the more *vaccine doses received* and the higher the degree of *threat perceived by COVID-19*, the more positive participant attitudes were towards both financial incentives. Negative attitudes towards *money* and *lottery* are instead more often prevalent among participants who are older, who are positive towards homeopathy, who perceive a high degree of social division in society, and who have a conscientious personality.

Additional crosschecks for Outcome I show that, among those participants who received at least one dose of SARS-CoV-2 vaccination, while controlling for the self-perceived importance of six concrete drivers for the decision to have a (further) dose of vaccine– protecting self, protecting others, official medical advice/recommendation, participation in public events, vocational mandates, and peer pressure (Supplementary Fig. 5)– the associations that were found in the main models between the frames and attitudes toward the four incentives remain highly robust (Supplementary Table 3). Regarding associations between the aforementioned immunization drivers and the four immunization incentives tested here, we find that the more important participants rate the immunization driver *protection of others* as a driver for their own vaccination decision, the more positively they view *football tickets*, *sausage* and *lottery* as incentives, but not *money*. With respect to the latter, only persons who indicated, that *peer pressure* was an important driver for their immunization decision regard money as a meaningful incentive. It is noteworthy, that *protecting self*, the driver rated on average as most important overall, has no significant association with any of the incentives. While a higher degree of self-perceived knowledge of SARS-CoV-2 vaccination (Supplementary Table 4) is positively associated with a favorable view of immunization incentives *football*, *sausage*, and *lottery* (once again, not *money*), controlling for self-perceived knowledge does not alter the overall effects of the frames on views towards all four incentives. Moreover, controlling for statutory/private health insurance status does not affect the associations found between the frames and views on incentives (Supplementary Table 5).

**Fig. 7:**
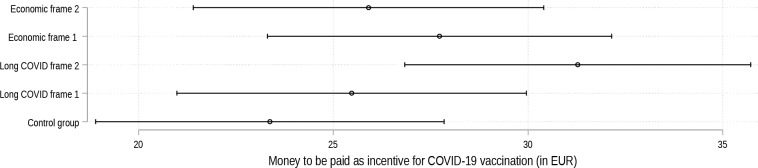
Money considered acceptable as incentive for SARS-COV-2 vaccination. Annotation: *N* = 6,685. Survey data from Politikpanel Deutschland, 06/30 − 07/17/2022 (https://www.politikpanel.uni-freiburg.de/). Predictions based on OLS regression + 95% CI. The model additionally controls for the full set of controls as in Fig. 6. See Supplementary Table 6 for the full model

Figure 7 presents the results for Outcome II, i.e. how much money the different experimental groups would consider acceptable vis-à-vis the control group as compensation for SARS-CoV-2 vaccination. The model, which controls for the same range of factors as the models for Outcome I, predicts that, on average, participants in all experimental groups consider a larger amount of money to be adequate as an incentive than those in the control group. Yet, this association is only significant for Long COVID frame 2 (*p* < 0.05), i.e. 7.90 EUR more than the control group. Additional models controlling for concrete drivers of vaccination decisions, self-perceived knowledge about SARS-CoV-2 vaccination and the type of health insurance yield no significantly different results (Supplementary Table 6). The predicted amount of money considered appropriate as an incentive for the pooled framing groups (either the two Long COVID and the two Economic frames, or all four frames pooled), are significantly (*p* < 0.05) larger than the control group’s mean (see Supplementary Fig. 7).

**Fig. 8:**
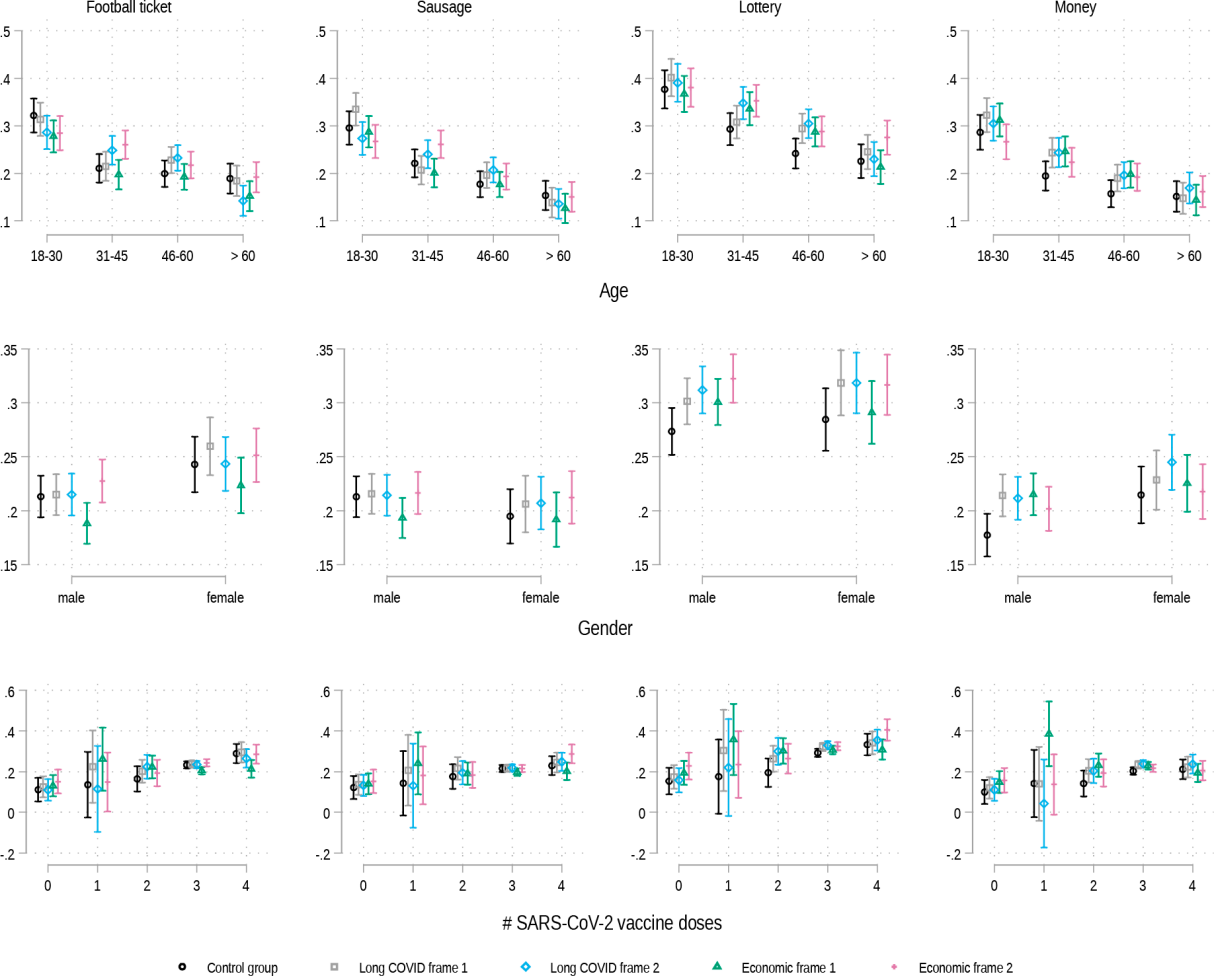
Predicted meaningfulness of vaccination incentives based on interaction models: frame x age group/gender/number of SARS-CoV-2 vaccine doses received. Annotation: *N* = 6,685. Survey data from Politikpanel Deutschland, 06/30 − 07/17/2022 (https://www.politikpanel.uni-freiburg.de/). Predictions based on OLS regression including interactions + 95% CI

Interaction models testing to what extent specific frames show bigger effects among certain groups of participants with respect to specific incentives yield only few statistically significant results. Figure 8 exemplifies this, presenting the results from models which include the interaction terms frames*age, frames*gender and frames*number of SARS-CoV-2 vaccine doses. The figure shows that most frames have very similar effects among the experimental groups, yet, some noteworthy differences exist: e.g. Economic frame 2 has a bigger positive effect on the attitudes towards the incentives *football ticket* and *sausage* in the age group 31–45 compared to the three other frames. Economic frame 2 has the smallest effect among this age group concerning the incentive *money*. With respect to both financial incentives, all frames work best in the age groups 31–45 and 45–60. While female participants are generally more positive towards the incentive *money* than men, all four frames show bigger effects for males than for women. The graphs in the bottom row of Fig. 8 show that, overall, there exist only small differences in the framing effects by immunization status. Those participants who had received none, or only one dose of the SARS-CoV-2 in most cases rated the meaningfulness of the four incentives very similarly regardless of whether they were in the control or one of the experimental groups. Only the incentive *money* was rated as much more meaningful by those participants who had received only one dose, if they were presented with Economic frame 1.

## Discussion

Based on a randomized, controlled framing experiment in a final cohort of *n* = 6,685 respondents to a German survey (June/July 2022), we tested whether four different framing messages differentially affect respondents’ attitudes towards four SARS-CoV-2 immunization incentives (Outcome I), namely two non-financial (*free grilled sausage*, *free football tickets*) and two financial alternatives (*participation in immunization lottery [high payout]*, *direct monetary compensation for immunization*). The frames described individual or collective consequences (covering adverse health effects, social function losses, and/or economic losses) as a result of severe COVID-19 or Long COVID. Across the entire sample, the vast majority of participants (60.3-73.78%, depending on the incentive under consideration) viewed all four of the incentives we tested as *less* or *not meaningful*. Not surprisingly, individuals who had received ≥2 doses (i.e. fully vaccinated) were significantly less negative towards all four incentives than those who were not/not fully vaccinated (i.e. had received ≤1 dose). We explored, in a separate question (Outcome II), which level was deemed meaningful if direct monetary compensation for immunization were to be introduced. While ~ 77% of the sample were not willing to pay any sum as direct compensation, we found differential views across the remaining participants ranging from (on average) < 7 EUR (not in favor of direct compensation) to 145 EUR (highly in favor). 1.6% of fully vaccinated and 5.1% of not/not fully vaccinated individuals even favored the highest payment category (≥500 EUR).

Regarding framing effects, based on multiple regression analysis, we found that– despite an unexpected negative effect of Economic frame 1 (*high costs of COVID-19 hospitalization: proposal of compulsory immunization and out-of-pocket cost sharing of unvaccinated*) on the meaningfulness of the incentive *football tickets*– none of the frames had significant effects on the non-financial incentives (*sausage*, *football tickets*). Nevertheless, both Long COVID frames (frame 1: *general example– risk of developing mild to life-changing sequelae because of COVID-19*; frame 2: *concrete*,* personal example– life-changing sequelae and risk of poverty because of COVID-19*) increase the likelihood of participants indicating positive attitudes towards incentives *lottery* and *money*. While Economic frame 1 increases the likelihood of participants indicating positive attitudes towards *money*, Economic frame 2 (*high costs of COVID-19 hospitalization: serious cases cost more than treatment of cancer patients and injuries caused by accidents*) increases the likelihood of participants indicating positive attitudes towards *lottery*. Those participants who read Long COVID frame 2 also considered significantly higher amounts of direct monetary payments to be adequate as incentives: 23.33 EUR (95% CI: 18.89–27.85 EUR) in the control group; 31.28 EUR (95% CI: 26.84–35.72 EUR) in the Long COVID frame 2 group. Additional interaction tests show that all four frames have very similar effects across several subgroups (age-cohorts, gender, number of SARS-CoV-2 vaccine doses)– i.e. most of the framing effects are not significantly moderated by these three factors. The only stronger interaction can be found for gender: male participants who received any of the four frames were significantly more positive towards *money* as an incentive, while for women only the Long COVID frame 2 had a slightly positive impact on the incentive *money* (the other three frames showed no effect on women’s’ attitude towards this incentive).

The majority of published studies attempt to identify associations between (a) frames, or groups thereof, and (b) vaccination intentions. However, these studies provide conflicting evidence, and it is highly challenging if not impossible to properly compare their findings and potentially draw conclusions for other settings. In Italy, for example, a study found that frames covering personal and collective health risks and national economic risk (e.g. employment loss) increased immunization intentions overall but, in direct comparison, did not have a significant differential impact on vaccine uptake [53]. In the US, a study found that economic vis-à-vis health risk frames make a difference along party lines– a collective economic consequences of the pandemic frame tended to increase Republicans’ intention to have a SARS-CoV-2 vaccine while the respective collective health consequences frame tended to increase Democrats’ intention to have the vaccine [54]. Conversely, another study in the US found that– irrespective of partisanship– both a personal and a collective health consequences frame increased immunization intention across the board while an economic cost frame had little effect [55]. In any case, vis-à-vis the control group, all frames in our study (6 out of 8 statistically significantly; *p* < 0.05) increased the likelihood of favorable views towards the two financial immunization incentives (*lottery* & *money*) independent of a participant’s immunization status (0–4 doses of vaccine). On the attitudes towards the two other non-financial incentives free *football ticket* and grilled *sausage* the frames had no similar positive effects.

Other studies have explored the effects of positive or negative attribute frames on immunization intentions [29] and their associations with more or less well-known vaccines [56]. While the evidence is, once again, somewhat conflicting as the former study shows little effect on intentions, the authors of the latter study conclude that negative attribute frames increase immunization intentions for better known vaccines and positive attribute frames for less well-known vaccines (in this case for Moderna’s mRNA vaccine in 2021). We tested the effects of negative attribute frames only, and attitudes towards immunization incentives are arguably of a different nature than intentions, however, positive attribute frames, e.g. highlighting the individual and collective benefits of consequences averted (the latter of which will, for most individuals, be less-well known/abstract concepts before they are exposed to these consequences directly), might have had a different effect on the number or magnitude of favorable views towards the four immunization incentives in our study. This might be even more so the case for those with a higher perceived threat by COVID-19. Therefore, future studies should focus on exploring the effects of positive attribute frames on attitudes towards incentives.

Our overall results suggest that not the particular narrative of a frame but its existence can be highly important. This being said, while we used framing messages that had differential foci (economic & Long COVID), our frames remained somewhat broad in nature, and it is entirely possible that we would have observed different effects and effect magnitudes if we had designed and tested frames for more specific target groups. It would, therefore, be intriguing to explore the effects of frames designed to resonate with so-called attitude roots, a concept coined by Hornsey and Fielding in 2017 [57]. Attitude roots are “underlying fears, ideologies, worldviews, and identity needs that sustain and motivate specific ‘surface’ attitudes like climate scepticism and creationism.” Hornsey and Fielding identified six types of attitude roots and concluded that public campaigns attempting change behaviour should not focus on changing peoples’ minds about an issue but highlighting the benefits of showing a desired behaviour within the context of their attitude roots, i.e. providing a basis for rationalisation. Empirically testing the effects of differential immunisation uptake and incentive framing messages designed to tie into existing attitude roots would likely be a worthwhile endeavour– in particular as more recent research is exploring COVID-19 specific attitude roots [58].

Our study has a number of strengths that set it apart from previous research. First, it tested the effects of two concrete frame groups that describe, on the one hand, individual and collective, and, on the other, health and economic consequences resulting from severe COVID-19 or Long COVID (an often suboptimally addressed topic in public health messaging) on incentives for vaccination that were proposed or implemented in Germany during the height of the SARS-CoV-2 pandemic in a large-n study of individuals in Germany, while controlling for a host of further potential explanatory factors. Second, in the absence of vaccine administration data permitting randomized controlled trials [41, 47, 59], many other studies use data on vaccine intentions– a proxy measure that can be seriously flawed. We use a different approach: our study measures the effects of incentives on vaccination based on participant attitudes towards these incentives and controls for de facto immunization status to explore potential differences in responses due to concrete outcomes of underlying vaccine willingness vis-à-vis refusal/hesitance. The limitations of this study include that it is based on a survey conducted in Germany only. In addition, while our main interest was to better understand the effects of four framing messages on attitudes towards incentives, we did not elicit the beliefs of participants regarding the effectiveness of the incentives under consideration and thus did not distinguish between effectiveness beliefs and attitudes towards the incentives (our survey asked for the perceived meaningfulness of vaccination incentives, see Supplementary Table 7). Although transferability of the results to other countries is not readily possible, as there is little scientific consensus on the effects of specific framing messages and our results suggest that the presence of a frame rather than its specific content is the central element of persuasion, this might be a universal effect also found in other settings. A further limitation is that our participants were self-selecting. Nevertheless, the sample shows sufficient variation with regard to the potential socio-demographic and psychological grouping factors we tested. Moreover, we were not able to test if the effects and associations measured here remain stable over time, as this was a cross-sectional analysis.

## Conclusion

In public health, even a 1% point change in behavior often has a substantial effect on outcomes– in this case a 1% point change could reduce the risk of severe COVID-19 or Long COVID for ~ 850,000 Germans. As our data show that all four framing strategies indeed have a small, yet statistically significant (or at least by trend) positive effect on attitudes towards immunization incentives (even among the unvaccinated), we contend that immunization rate improvement strategies should always make the effort– even if resources are scarce– to include some sort of short-form narrative framing. Naturally, also the source of the narrative– ‘the messenger’– can make an important difference as to how narratives and frames affect uptake (see e.g [60]). Although our randomized, controlled survey experiment shows that the majority of our sample from Germany is not in favor of either financial nor non-financial incentives for SARS-CoV-2 vaccination [12], social capital can be key in convincing individuals and groups thereof to have or reject a vaccine. Although framing has a positive effect on incentives, it is important that further sociological effects are systematically utilized to complement immunization campaigns.

## Electronic supplementary material

Below is the link to the electronic supplementary material.

Supplementary Material 1

## Data Availability

The dataset and Stata code supporting the conclusions of this article is available in the Harvard Dataverse repository, 10.7910/DVN/5RW50M.

## Footnotes

1 For the calculation of these arithmetic means the answers from the original 6-point scale were recoded to the means of the categories (e.g. “20 to 50 Euro” ➔ 35 Euro) except for the first and the last categories (“nothing” and “500 and more Euro”) which were coded as 0 and 500. The resulting metric scale is also applied as Outcome II in the following.

2 The results for the other two incentives (*football tickets* and *sausage*) can be found in Supplementary Table 2.
