# Supplementary material for: Economic and health consequence frames affect COVID-19 vaccine incentive attitudes in Germany– a survey based framing experiment
Source: BMC Public Health. 2025 Jun 4;25:2071. doi: 10.1186/s12889-025-23279-x (PMC12135310; doi:10.1186/s12889-025-23279-x)
Supplement: Supplementary file 1 — Supplementary Material 1 [file 12889_2025_23279_MOESM1_ESM.docx]

**Supplementary Material**

Jäckle, Sebastian /Timmis, James K. (2025): Economic and health consequence frames affect COVID-19 vaccine incentive attitudes in Germany – a survey based framing experiment, in BMC Public Health

[Checklist for Reporting Of Survey Studies (CROSS) for: *Economic and health consequence frames affect COVID-19 vaccine incentive attitudes in Germany – a survey based framing experiment* 2](#_Toc199450795)

[Supplementary Note 1: Frames 5](#_Toc199450796)

[Supplementary Note 2: Consent to participate – German original 10](#_Toc199450797)

[Supplementary Table 1: Means of outcome variables of control group and the pooled framing groups (+ results of mean differences tests) 12](#_Toc199450798)

[Supplementary Table 2: Outcome I - Main models, multiple OLS regression (b-coefficients) 13](#_Toc199450799)

[Supplementary Table 3: Outcome I - Cross check models A: concrete drivers for vaccination decision, multiple OLS regression (b-coefficients) 17](#_Toc199450800)

[Supplementary Table 4: Outcome I - Cross check models B: self perceived knowledge about COVID-19 vaccination, multiple OLS regression (b-coefficients) 21](#_Toc199450801)

[Supplementary Table 5: Outcome I - Cross check models C: private health insurance, multiple OLS regression (b-coefficients) 25](#_Toc199450802)

[Supplementary Table 6: Outcome II - Main model & cross check models, multiple OLS regression (b-coefficients) 29](#_Toc199450803)

[Supplementary Table 7: Survey Questions 33](#_Toc199450804)

[Supplementary Table 8: Missing values – item non-response 40](#_Toc199450805)

[Supplementary Table 9: Descriptive statistics for the original dataset and the final dataset used in the regression models (reduced via listwise deletion) 42](#_Toc199450806)

[Supplementary Figure 1: Descriptive statistics of dependent variables by gender 45](#_Toc199450807)

[Supplementary Figure 2: Associations between frames and dependent variables – bivariate regression analyses, comparison between OLS and ordinal logit models (reference = control group; + 95% CI, full sample) 46](#_Toc199450808)

[Supplementary Figure 3: Associations between frames and dependent variables – multiple regression analyses, comparison between OLS and Ologit models (reference = control group; + 95% CI, full sample) 47](#_Toc199450809)

[Supplementary Figure 4: Predicted meaningfulness of vaccination incentives based on multiple OLS models with pooled frames 48](#_Toc199450810)

[Supplementary Figure 5: Average importance of concrete drivers for SARS-CoV-2 vaccination 49](#_Toc199450812)

[Supplementary Figure 6: Distribution of main demographic variables in the sample compared to reality 50](#_Toc199450813)

[Supplementary Figure 7: Money deemed acceptable as incentive for COVID-19 vaccination (pooled frames: a) Long COVID frames/Economic frames; b) all four frames pooled) 51](#_Toc199450814)

[Literature 51](#_Toc199450815)

## Checklist for Reporting Of Survey Studies (CROSS) for: *Economic and health consequence frames affect COVID-19 vaccine incentive attitudes in Germany – a survey based framing experiment*

| **Section/topic** | **Item** | **Item description** | **Reported on page #** |
| --- | --- | --- | --- |
| **Title and abstract** | | |  |
| Title and abstract | 1a | State the word “survey” along with a commonly used term in title or abstract to introduce the study’s design. | 1 |
|  | 1b | Provide an informative summary in the abstract, covering background, objectives, methods, findings/results, interpretation/discussion, and conclusions. | 2 |
| **Introduction** | | |  |
| Background | 2 | Provide a background about the rationale of study, what has been previously done, and why this survey is needed. | 3-7 |
| Purpose/aim | 3 | Identify specific purposes, aims, goals, or objectives of the study. | 6/7 |
| **Methods** | | |  |
| Study design | 4 | Specify the study design in the methods section with a commonly used term (e.g., cross-sectional or longitudinal). | 7 |
|  | 5a | Describe the questionnaire (e.g., number of sections, number of questions, number and names of instruments used). | 7-9 |
| Data collection methods | 5b | Describe all questionnaire instruments that were used in the survey to measure particular concepts. Report target population, reported validity and reliability information, scoring/classification procedure, and reference links (if any). | 7-9 |
|  | 5c | Provide information on pretesting of the questionnaire, if performed (in the article or in an online supplement). Report the method of pretesting, number of times questionnaire was pre-tested, number and demographics of participants used for pretesting, and the level of similarity of demographics between pre-testing participants and sample population. | 8 |
|  | 5d | Questionnaire if possible, should be fully provided (in the article, or as appendices or as an online supplement). | Supp. Tab. 7 |
| Sample characteristics | 6a | Describe the study population (i.e., background, locations, eligibility criteria for participant inclusion in survey, exclusion criteria). | 8 |
|  | 6b | Describe the sampling techniques used (e.g., single stage or multistage sampling, simple random sampling, stratified sampling, cluster sampling, convenience sampling). Specify the locations of sample participants whenever clustered sampling was applied. | 8 |
|  | 6c | Provide information on sample size, along with details of sample size calculation. | 8 |
|  | 6d | Describe how representative the sample is of the study population (or target population if possible), particularly for population-based surveys. | 8, 10, Supp.  Fig. 6 |
| Survey  administration | 7a | Provide information on modes of questionnaire administration, including the type and number of contacts, the location where the survey was conducted (e.g., outpatient room or by use of online tools, such as SurveyMonkey). | 8 |
|  | 7b | Provide information of survey’s time frame, such as periods of recruitment, exposure, and follow-up days. | 8 |
|  | 7c | Provide information on the entry process:  –>For non-web-based surveys, provide approaches to minimize human error in data entry.  –>For web-based surveys, provide approaches to prevent “multiple participation” of participants. | 8 |
| Study preparation | 8 | Describe any preparation process before conducting the survey (e.g., interviewers’ training process, advertising the survey). | 8 |
| Ethical considerations | 9a | Provide information on ethical approval for the survey if obtained, including informed consent, institutional review board [IRB] approval, Helsinki declaration, and good clinical practice [GCP] declaration (as appropriate). | 8 |
|  | 9b | Provide information about survey anonymity and confidentiality and describe what mechanisms were used to protect unauthorized access. | 8 |
| Statistical  analysis | 10a | Describe statistical methods and analytical approach. Report the statistical software that was used for data analysis. | 10 |
|  | 10b | Report any modification of variables used in the analysis, along with reference (if available). | 9 |
|  | 10c | Report details about how missing data was handled. Include rate of missing items, missing data mechanism (i.e., missing completely at random [MCAR], missing at random [MAR] or missing not at random [MNAR]) and methods used to deal with missing data (e.g., multiple imputation). | 8 and Supp.Tab. 8 & 9 |
|  | 10d | State how non-response error was addressed. | 8 |
|  | 10e | For longitudinal surveys, state how loss to follow-up was addressed. | n/a |
|  | 10f | Indicate whether any methods such as weighting of items or propensity scores have been used to adjust for non-representativeness of the sample. | 8 |
|  | 10g | Describe any sensitivity analysis conducted. | 10, Supp. Fig. 2 & 3 |
| **Results** | | |  |
| Respondent characteristics | 11a | Report numbers of individuals at each stage of the study. Consider using a flow diagram, if possible. | 11 |
|  | 11b | Provide reasons for non-participation at each stage, if possible. | 11 |
|  | 11c | Report response rate, present the definition of response rate or the formula used to calculate response rate. | 11 |
|  | 11d | Provide information to define how unique visitors are determined. Report number of unique visitors along with relevant proportions (e.g., view proportion, participation proportion, completion proportion). | 11 |
| Descriptive  results | 12 | Provide characteristics of study participants, as well as information on potential confounders and assessed outcomes. | 12-14 & Supp. Tab. 9 |
| Main findings | 13a | Give unadjusted estimates and, if applicable, confounder-adjusted estimates along with 95% confidence intervals and p-values. | 12-16 |
|  | 13b | For multivariable analysis, provide information on the model building process, model fit statistics, and model assumptions (as appropriate). | 10, 16-19 |
|  | 13c | Provide details about any sensitivity analysis performed. If there are considerable amount of missing data, report sensitivity analyses comparing the results of complete cases with that of the imputed dataset (if possible). | 10, 15-16, Supp. Fig. 2 & 3 Supp. Tab. 3-6 |
| **Discussion** | | |  |
| Limitations | 14 | Discuss the limitations of the study, considering sources of potential biases and imprecisions, such as non-representativeness of sample, study design, important uncontrolled confounders. | 23 |
| Interpretations | 15 | Give a cautious overall interpretation of results, based on potential biases and imprecisions and suggest areas for future research. | 21-23 |
| Generalizability | 16 | Discuss the external validity of the results. | 23 |
| **Other sections** | | |  |
| Role of funding source | 17 | State whether any funding organization has had any roles in the survey’s design, implementation, and analysis. | 1 |
| Conflict of interest | 18 | Declare any potential conflict of interest. | 1 |
| Acknowledgements | 19 | Provide names of organizations/persons that are acknowledged along with their contribution to the research. | n/a |

## Supplementary Note 1: Frames

Control Group

*German original*

An dieser Stelle folgen nun noch ein paar weitere Fragen zu Impfungen.

*English translation*

Here are a few more questions about vaccinations.

Long COVID frame 1 (general example): Risk of range of mild to life-changing sequelae

*German original*

An dieser Stelle folgen nun noch ein paar weitere Fragen zu Impfungen.

Über die möglichen, langfristigen Auswirkungen einer COVID-19 Infektion (Long COVID) wird weiterhin viel diskutiert.
Lesen Sie bitte hierzu diesen kurzen Textauszug aus einem Nachrichtenartikel.

**Pandemie: Genesen, aber nicht gesund: Long COVID weiter im Fokus**

In einer Stellungnahme des Corona-Expertenrats der Bundesregierung aus dem Mai heißt es, laut Studien entwickle die Mehrheit derer, die mit schwerem COVID-19-Verlauf auf Intensivstationen behandelt wurden, Langzeitkomplikationen. Auch nach milder Infektion erfüllten zehn Prozent die Post-COVID-Kriterien. [...]

Jördis Frommhold, Lungenfachärztin und Chefärztin der Median Klinik Heiligendamm, geht von hunderttausenden Long-COVID-Betroffenen in Deutschland aus. Konsens in Expertenkreisen herrscht zur Annahme, dass vollständiger Impfschutz das Risiko für Langzeitfolgen nach einer Corona-Infektion klar verringert. Einer englischen Studie zufolge reduzieren Grundimpfungen und Booster das Long-COVID-Risiko um 50 Prozent, einer israelischen Studie zufolge um zwei Drittel. [...]

Frommhold betont, wie groß die Abstufung bei möglichen Symptomen sei – viele schränkten im Alltag kaum oder gar nicht ein, andere könnten im Extremfall zu längerfristiger Arbeitsunfähigkeit oder Bettlägerigkeit führen. [...]

Zu den häufigsten Symptomen gehört eine pathologische, als «Fatigue» bezeichnete Müdigkeit. Beeinträchtigungen der Leistungs- und Merkfähigkeit, der Konzentration oder ein «Gehirnnebel» (Brain Fog) treten auch oft auf. Wortfindungsstörungen und weitere kognitive Einschränkungen werden häufig beklagt, ebenso wie allgemeine Schwäche, Atemnot oder Kurzatmigkeit und andauernder Husten.

*Quelle: ZEIT ONLINE 08.06.2022*

*English translation*

Here are a few more questions about vaccination.

There is still a lot of discussion about the possible long-term effects of a COVID-19 infection (Long COVID).

Please read this short text excerpt from a news article.

**Pandemic: Recovered but not healthy: Long COVID still in focus**

A May statement by the federal government's Corona Expert Council said that according to studies, the majority of those treated in intensive care units with severe covid 19 developed long-term complications. Even after mild infection, ten percent met the post-COVID criteria. [...]

Jördis Frommhold, lung specialist and head physician at the Median Clinic Heiligendamm, assumes that hundreds of thousands of people in Germany are affected by Long COVID. There is a consensus among experts that full vaccination protection clearly reduces the risk of long-term consequences after a corona infection. According to an English study, basic vaccinations and boosters reduce the long-covid risk by 50 per cent, according to an Israeli study by two thirds. [...]

Frommhold emphasises how great the gradation of possible symptoms is - many hardly restrict or do not restrict everyday life at all, others can lead to long-term incapacity to work or bedriddenness in extreme cases. [...]

One of the most common symptoms is a pathological tiredness known as "fatigue". Impairments in performance and memory, concentration or a "brain fog" also often occur. Word-finding disorders and other cognitive impairments are frequently complained of, as are general weakness, shortness of breath and persistent coughing.

*Source: ZEIT ONLINE 08.06.2022*

Long COVID frame 2 (specific, emotional example): Risk of poverty due to life-changing sequelae

*German original*

An dieser Stelle folgen nun noch ein paar weitere Fragen zu Impfungen.

Über die möglichen, langfristigen Auswirkungen einer COVID-19 Infektion (Long COVID) wird weiterhin viel diskutiert.
Lesen Sie bitte hierzu diesen kurzen Textauszug aus einem Nachrichtenartikel.

**Eingestellte Krankenkassenzahlungen – Long-COVID-Patienten droht der finanzielle Absturz**

Schätzungsweise zehn Prozent aller Coronainfizierten, die sich nach vollständiger Impfung mit Sars-CoV-2 angesteckt haben, leiden einer Studie zufolge unter Long COVID. Das wäre in Deutschland annähernd eine halbe Million Menschen, von denen niemand weiß, wie lange ihre Beschwerden anhalten und ob sie je wieder vollständig gesund werden.

Für viele Betroffene kommt nun eine weitere Sorge hinzu. Sie erfahren, dass ihre finanzielle Absicherung in Gefahr ist, weil Arbeitgeber und Krankenkassen nicht länger zahlen. Das Arbeitslosengeld fließt für ein bis zwei Jahre. Danach folgt das ALG II, besser bekannt als Hartz IV. Was das bedeutet, nennt Thorsten Schäfer von der Hamburger Beratungsstelle Arbeit und Gesundheit das »volle Hartz-IV-Programm«: Das Ersparte muss aufgebraucht werden, Wohnungsgrößen werden geprüft, Hilfssätze gekürzt, wenn der Partner gut verdient. »Eine krasse Abstiegserfahrung für Menschen, die teils Jahrzehnte in das System eingezahlt haben«, sagt Schäfer. [...]

Natascha S., 34 Jahre alt, hat Long COVID. [...] Aber Natascha hat noch Glück, sie wohnt mietfrei in ihrem früheren Elternhaus. Sonst käme sie jetzt nicht mehr über die Runden, sagt sie. Ihre Mutter lebt in der Nähe, unterstützt sie, wo sie kann. Ihr Mann hat seine Arbeitszeit auf 30 Stunden reduziert, damit er sich um sie kümmern kann – das belastet die Familienfinanzen zusätzlich.

Sie hofft noch auf eine Erwerbsunfähigkeitsrente, doch der Weg dahin ist bürokratisch und kompliziert. Schon die Verfahren zur Anerkennung von Long COVID als Berufskrankheit oder Arbeitsunfall dauern nicht selten ein Jahr oder länger. Solange nichts entschieden ist, erhalten die Betroffenen oft erst einmal gar kein Geld.

*Quelle: Spiegel 11.06.2022*

*English translation*

Here are a few more questions about vaccination.

There is still a lot of discussion about the possible long-term effects of a COVID-19 infection (Long COVID).

Please read this short text excerpt from a news article.

**Discontinued health insurance payments - Long COVID patients face financial crash**

According to a study, an estimated ten per cent of all corona patients who have contracted Sars-CoV-2 after being fully vaccinated suffer from Long COVID. In Germany, that would be almost half a million people, of whom no one knows how long their symptoms will last and whether they will ever fully recover.

For many of those affected, there is now an additional worry. They find out that their financial security is in danger because employers and health insurance companies no longer pay. Unemployment benefit is paid for one to two years. This is followed by ALG II, better known as Hartz IV. Thorsten Schäfer from the Hamburg counselling centre Arbeit und Gesundheit (Work and Health) calls what this means the "full Hartz IV programme": savings have to be used up, flat sizes are checked, assistance rates are reduced if the partner earns well. "A blatant experience of decline for people who have paid into the system, in some cases for decades," says Schäfer. [...]

Natascha S., 34 years old, has Long COVID. [...] But Natascha is still lucky, she lives rent-free in her former parents' house. Otherwise she wouldn't be able to make ends meet now, she says. Her mother lives nearby and supports her where she can. Her husband has reduced his working hours to 30 so that he can take care of her - this puts an additional strain on the family finances.

She still hopes for a disability pension, but the path to it is bureaucratic and complicated. Even the procedures for recognising Long COVID as an occupational disease or accident at work often take a year or more. As long as no decision has been made, those affected often receive no money at all.

*Source: Spiegel 11.06.2022*

Economic frame 1: high costs of COVID-19 hospitalization: compulsory immunisation and out-of-pocket cost sharing (unvaccinated)

*German original*

An dieser Stelle folgen nun noch ein paar weitere Fragen zu Impfungen.

Behandlungskosten einer COVID-19 Erkrankung im Krankenhaus können hoch sein.

Lesen Sie bitte hierzu diesen kurzen Textauszug aus einem Nachrichtenartikel.

**Das kostet die Behandlung ungeimpfter Corona-Patienten**

Schon für einen einzelnen Patienten liegt der Preis für die Behandlung auf einer Intensivstation bei mehreren Tausend Euro. So schlagen die zehn Prozent der beatmungspflichtigen Patienten mit den höchsten Kosten von etwa 77.000 Euro zu Buche. [...] Berechnet man den Durchschnitt, liegen die Kosten pro Fall bei 10.200 Euro.

Die deutsche Krankenhausgesellschaft beschrieb den durchschnittlichen COVID-Intensivpatienten unlängst als "derzeit ungeimpft, männlich und mittleren Alters". Durch das gesunkene Durchschnittsalter der Patienten müssen die Ärzte und Pfleger länger um das Leben der Erkrankten kämpfen. Aktuell trägt diese Kosten die Solidargemeinschaft.

Vor rund zwei Wochen hatte sich die Kassenärztliche Vereinigung Berlin (KV) für eine allgemeine Impfpflicht und eine Kostenbeteiligung Ungeimpfter an Krankenhausleistungen ausgesprochen. [...] "Mit einer Impfpflicht allein kommen wir hier nicht mehr weiter", hieß es vom KV-Vorstand.

*Quelle: T-Online 01.12.2021*

*English translation*

Here are a few more questions about vaccination.

Treatment costs of COVID-19 disease in hospital can be high.

Please read this short text excerpt from a news article.

**The cost of treating unvaccinated Corona patients**

Even for a single patient, the price of treatment in an intensive care unit is several thousand euros. For example, the ten percent of patients requiring ventilation have the highest costs of about 77,000 euros. [...] If one calculates the average, the costs per case are 10,200 euros.

The German Hospital Association recently described the average covid intensive care patient as "currently unvaccinated, male and middle-aged". Due to the lower average age of the patients, doctors and nurses have to fight longer for the life of the sick. Currently, these costs are borne by the solidarity community.

About a fortnight ago, the Association of Statutory Health Insurance Physicians (KV) in Berlin spoke out in favour of a general vaccination obligation and cost sharing for unvaccinated patients in hospital services. [...] "We can no longer get anywhere with compulsory vaccination alone," said the KV board.

*Source: T-Online 01.12.2021*

Economic frame 2: high costs of COVID-19 hospitalization: serious cases cost more than treatment of cancer patients and injuries caused by accidents

*German original*

An dieser Stelle folgen nun noch ein paar weitere Fragen zu Impfungen.

Behandlungskosten einer COVID-19 Erkrankung im Krankenhaus können hoch sein.

Lesen Sie bitte hierzu diesen kurzen Textauszug aus einem Nachrichtenartikel.

**Krankenkassen rechnen vor: Corona-Patienten auf Intensivstation kosten zehntausende Euro**

[...] Eine Auswertung des wissenschaftlichen Dienstes der AOK rechnete vor, dass die stationäre Behandlung eines Corona-Patienten, der mit einer Ecmo-Maschine beatmet werden muss, im Schnitt 92.000 Euro kostet. [...] Nimmt man alle Fälle zusammen, kommt man auf einen Durchschnitt von 10.200 Euro pro Patienten.

Auf etwa 1.500 bis 3.000 Euro täglich bezifferte etwa der Referatsleiter Krankenhausfinanzierung der Landeskrankenhausgesellschaft, Andreas Weichert, die Kosten für die Intensivbehandlung. Mehrere Intensivmediziner hatten die durchschnittliche Liegezeit von COVID-Patienten auf Intensivstationen auf zwei bis drei Wochen geschätzt.

"Der Aufwand für die Behandlung schwer erkrankter Corona-Patienten ist wesentlich höher als beispielsweise der Aufwand für onkologische Patienten beziehungsweise für die Versorgung von Unfallopfern", erklärte Weichert.

*Quelle: FOCUS Online 05.01.2022*

*English translation*

Here are a few more questions about vaccination.

Treatment costs of COVID-19 disease in hospital can be high.

Please read this short text excerpt from a news article.

**Health insurance companies calculate: Corona patients in intensive care cost tens of thousands of euros**

[...] An evaluation of the scientific service of the AOK calculated that the inpatient treatment of a Corona patient who has to be ventilated with an ECMO machine costs on average 92,000 euros. [...] If you take all cases together, you come to an average of 10,200 euros per patient.

Andreas Weichert, head of the hospital financing department of the state hospital association, put the costs for intensive care treatment at about 1,500 to 3,000 euros per day. Several intensive care physicians had estimated the average length of stay of COVID patients in intensive care units at two to three weeks.

"The cost of treating seriously ill corona patients is much higher than, for example, the cost of oncological patients or the cost of treating accident victims," explained Weichert.

*Source: FOCUS Online 05.01.2022*

## Supplementary Note 2: Consent to participate – German original

Das für das Politikpanel Deutschland verantwortliche Team des Lehrstuhls für Vergleichende Regierungslehre an der Universität Freiburg hält sich streng an die gesetzlichen Datenschutzvorschriften der Europäischen Datenschutz-Grundverordnung (DSGVO) und verarbeitet Ihre Daten ausschließlich zu wissenschaftlichen Zwecken.

Sämtliche personenbezogenen Angaben werden streng vertraulich behandelt und nur anonymisiert ausgewertet. Es ist damit keinerlei Rückschluss auf einzelne Personen möglich. Entsprechend erfolgen auch alle Veröffentlichungen von Forschungsergebnissen ausschließlich in anonymisierter Form und lassen zu keinem Zeitpunkt Rückschlüsse auf Sie als Person zu! Sofern Kontaktdaten erhoben werden, werden diese von den Umfragedaten getrennt gespeichert.

Bevor die Umfrage beginnt, möchten wir Sie noch darüber informieren, wie Ihre Daten geschützt werden.

| Kontakt | Prof. Dr. Uwe Wagschal  Albert-Ludwigs-Universität Freiburg  Seminar für Wissenschaftliche Politik  Werthmannstraße 12  79085 Freiburg  E-mail: politikpanel@politik.uni-freiburg.de |
| --- | --- |
| Welche personenbezogenen Daten werden erfasst und verarbeitet | Persönliche Daten (Geschlecht, Alter, Art der Krankenversicherung, Religion). Falls Sie am Gewinnspiel teilnehmen wollen, erfragen wir gesondert eine E-Mail-Adresse, die ausschließlich zur Benachrichtigung im Falle eines Gutschein-Gewinns genutzt und direkt im Anschluss an diese Benachrichtigung gelöscht wird. |
| Welche besondere Kategorien personenbezogener Daten werden erfasst und verarbeitet | Politische und gesellschaftliche Einstellungen |
| Gesetzliche Grundlage für die Verarbeitung | Die Verarbeitung der personenbezogenen Daten basiert auf der expliziten Zustimmung des Teilnehmers/der Teilnehmerin nach Art. 9 (2) Absatz a der DSGVO. |
| Empfänger und Kategorien von Empfängern von personenbezogenen Daten | Die personenbezogenen Daten können ausschließlich von den an dem Projekt beteiligten Wissenschaftlern/Wissenschaftlerinnen an den Universitäten Freiburg eingesehen und genutzt werden. Die Daten werden unter keinen Umständen an Dritte weitergegeben. |
| Automatisiertes Verfahren | Alle Daten der Umfrage sind auf einem bestmöglich gesicherten Server gespeichert (vgl. https://www.unipark.com/datenschutz/). Werden E-Mail-Adressen von Teilnehmenden erfragt, werden diese ausschließlich für die angegebenen Zwecke (erneute Befragung) genutzt. Die Mailingliste wird gemäß den Datenschutzstandards der Universität Freiburg auf universitätseigenen Servern betrieben. |
| Information zu den Rechten der Datensubjekte | Teilnehmerinnen und Teilnehmer haben die Möglichkeit unter politikpanel@politik.uni-freiburg.de eine Anfrage zu stellen bezüglich Zugang zu den eigenen Daten, einer Korrektur der eigenen Daten, einer Löschung der Daten, einer Einschränkung der Datenverarbeitung, einem Widerspruch zur Datenverarbeitung insgesamt, sowie der Datenübertragbarkeit (Art. 15-21 DSGVO). Um aus der Mailingliste des Politikpanel ausgetragen zu werden genügt eine E-Mail an politikpanel-liste-unsubscribe@politik.uni-freiburg.de. |
| Information über das Recht, die Zustimmung zu widerrufen | Teilnehmerinnen und Teilnehmer haben das Recht jederzeit ihre gegebene Zustimmung zu widerrufen. Vor einem solchen Widerruf der Zustimmung bleibt die Verarbeitung der Daten rechtmäßig. |
| Datenschutzbehörde | Teilnehmerinnen und Teilnehmer haben das Recht der Beschwerde bei einer Aufsichtsbehörde (Art. 77 DSGVO). |
| Wie lange werden die personenbezogenen Daten verarbeitet | 4 Jahre |

| □ | Ich stimme zu, dass meine personenbezogenen Daten gemäß den hier aufgeführten Angaben verarbeitet werden. | □ | Ich möchte nicht teilnehmen |
| --- | --- | --- | --- |

## Supplementary Table 1: Means of outcome variables of control group and the pooled framing groups (+ results of mean differences tests)

| Attitudes towards incentive … | Control | Compared to… | | |
| --- | --- | --- | --- | --- |
|  |  | any of the two Long COVID frames (pooled) | any of the two economic frames (pooled) | any of the four frames (pooled) |
|  |  | …via Dunett’s test | | … via two-sample t-test |
| football ticket | .2259 | .2262 | .2192 | .2227 |
| sausage | .2088 | .2104 | .2043 | .2073 |
| lottery | .2814 | .3085** | .3088** | .3087** |
| money | .1928 | .2189** | .2150* | .2170*** |
|  |  |  |  |  |
| How much money should be paid as incentive to get vaccinated? (in Euro) | 23.33 | 28.06 | 27.20 | 27.63* |

*Note:* N = 6,685. Survey data from Politikpanel Deutschland, 06/30-07/17/2022 (<https://www.politikpanel.uni-freiburg.de/>). Stars indicate significant differences to control group calculated via Dunnett's test/two sample t-test. * p < 0.1; ** p < 0.05; *** p < 0.01.

## Supplementary Table 2: Outcome I - Main models, multiple OLS regression (b-coefficients)

| Outcome I: Meaningfulness of … … as an incentive for vaccination | …free ticket for football match… | …free grilled sausage… | …vaccination lottery (chance to win big)… | …money… |
| --- | --- | --- | --- | --- |
| Frame (Reference: Control group) | | | | |
| Long COVID frame 1 | 0.00738 | 0.00656 | 0.0298^*^ | 0.0291^*^ |
|  | (0.67) | (0.61) | (2.40) | (2.58) |
|  |  |  |  |  |
| Long COVID frame 2 | 0.00224 | 0.00572 | 0.0374^**^ | 0.0326^**^ |
|  | (0.20) | (0.53) | (3.03) | (2.90) |
|  |  |  |  |  |
| Economic frame 1 | -0.0227^*^ | -0.0132 | 0.0198 | 0.0290^**^ |
|  | (-2.07) | (-1.24) | (1.61) | (2.59) |
|  |  |  |  |  |
| Economic frame 2 | 0.0125 | 0.00951 | 0.0425^***^ | 0.0168 |
|  | (1.13) | (0.88) | (3.42) | (1.49) |
|  |  |  |  |  |
| Number of COVID-19 vaccinations received (Reference: no vaccinations) | | | | |
| one vaccination received | 0.0555 | 0.0492 | 0.0816 | 0.0594 |
|  | (1.37) | (1.24) | (1.79) | (1.44) |
|  |  |  |  |  |
| two vaccinations received | 0.0780^***^ | 0.0574^**^ | 0.0875^***^ | 0.0703^***^ |
|  | (4.06) | (3.05) | (4.05) | (3.57) |
|  |  |  |  |  |
| three vaccinations received | 0.105^***^ | 0.0762^***^ | 0.135^***^ | 0.0978^***^ |
|  | (6.73) | (5.01) | (7.71) | (6.15) |
|  |  |  |  |  |
| four vaccinations received | 0.142^***^ | 0.105^***^ | 0.168^***^ | 0.0871^***^ |
|  | (7.33) | (5.54) | (7.68) | (4.39) |
|  |  |  |  |  |
| Immunization decision was very difficult and emotionally highly stressful for me. | -0.0344^*^ | -0.0518^**^ | -0.0732^***^ | -0.0216 |
|  | (-2.04) | (-3.14) | (-3.86) | (-1.25) |
|  |  |  |  |  |
| Immunization decision didn't take long for me. | 0.0485^***^ | 0.0422^**^ | 0.0231 | 0.0178 |
|  | (3.44) | (3.06) | (1.46) | (1.24) |
|  |  |  |  |  |
| How threatening? COVID-19 | 0.0881^***^ | 0.0946^***^ | 0.0891^***^ | 0.0810^***^ |
|  | (5.89) | (6.46) | (5.29) | (5.29) |
|  |  |  |  |  |
| Current COVID-19 safety perception | 0.0126 | 0.00820 | -0.0245 | -0.00127 |
|  | (0.73) | (0.49) | (-1.27) | (-0.07) |
|  |  |  |  |  |
| Waldorf education | 0.00569 | -0.0134 | 0.0302 | 0.00299 |
|  | (0.37) | (-0.89) | (1.74) | (0.19) |
|  |  |  |  |  |
| Homeopathy | -0.0356^*^ | -0.0624^***^ | -0.0830^***^ | -0.0446^**^ |
|  | (-2.56) | (-4.59) | (-5.30) | (-3.13) |
|  |  |  |  |  |
| Mainstream medicine | 0.0325 | 0.0541^**^ | 0.0749^***^ | 0.0144 |
|  | (1.65) | (2.81) | (3.37) | (0.71) |
|  |  |  |  |  |
| Religious denomination (Reference: no denomination) | | | | |
| Roman-Catholic | 0.0116 | -0.00637 | -0.00767 | -0.00659 |
|  | (1.18) | (-0.66) | (-0.69) | (-0.66) |
|  |  |  |  |  |
| Protestant | 0.0291^**^ | 0.0185 | 0.00995 | 0.00662 |
|  | (2.91) | (1.89) | (0.88) | (0.64) |
|  |  |  |  |  |
| Evangelical Free Church | -0.00135 | -0.00627 | -0.0216 | 0.0152 |
|  | (-0.10) | (-0.49) | (-1.47) | (1.14) |
|  |  |  |  |  |
| Orthodox Christian | -0.0427 | -0.00995 | 0.0934 | 0.0424 |
|  | (-0.62) | (-0.15) | (1.20) | (0.60) |
|  |  |  |  |  |
| Jewish | 0.160 | 0.145 | 0.0243 | -0.0552 |
|  | (1.86) | (1.72) | (0.25) | (-0.63) |
|  |  |  |  |  |
| Muslim | 0.0192 | -0.128^*^ | 0.0560 | -0.0194 |
|  | (0.37) | (-2.50) | (0.95) | (-0.36) |
|  |  |  |  |  |
| Other | 0.0233 | 0.0321 | -0.00388 | 0.00138 |
|  | (0.93) | (1.30) | (-0.14) | (0.05) |
|  |  |  |  |  |
| Left = 0/Right = 1 | -0.00834 | -0.0203 | 0.0502 | -0.0385 |
|  | (-0.30) | (-0.76) | (1.63) | (-1.37) |
|  |  |  |  |  |
| GAL = 0/TAN = 1 | -0.0110 | -0.0226 | -0.0820^***^ | -0.0278 |
|  | (-0.51) | (-1.07) | (-3.37) | (-1.25) |
|  |  |  |  |  |
| Voting Intention (Reference: CDU/CSU: Christian democrats) | | | | |
| SPD (Social democrats) | 0.0366^*^ | 0.0144 | 0.0423^*^ | 0.0154 |
|  | (2.46) | (0.99) | (2.52) | (1.01) |
|  |  |  |  |  |
| Greens | 0.0423^**^ | 0.0193 | 0.0643^***^ | 0.0332^*^ |
|  | (3.22) | (1.50) | (4.35) | (2.47) |
|  |  |  |  |  |
| FDP (Liberals) | 0.0328^*^ | 0.0109 | 0.0405^*^ | 0.0197 |
|  | (2.11) | (0.72) | (2.32) | (1.24) |
|  |  |  |  |  |
| Left-Party (Socialist) | 0.0249 | -0.0192 | 0.0150 | 0.0213 |
|  | (1.31) | (-1.03) | (0.70) | (1.10) |
|  |  |  |  |  |
| AfD (Populist right wing) | -0.0135 | -0.0276 | -0.0114 | 0.0114 |
|  | (-0.76) | (-1.59) | (-0.57) | (0.63) |
|  |  |  |  |  |
| Other | 0.00452 | -0.00927 | 0.0102 | 0.00533 |
|  | (0.32) | (-0.67) | (0.64) | (0.37) |
|  |  |  |  |  |
| Perception of social division | -0.102^***^ | -0.121^***^ | -0.118^***^ | -0.0736^**^ |
|  | (-4.00) | (-4.86) | (-4.13) | (-2.83) |
|  |  |  |  |  |
| Solidarity | 0.0823^**^ | 0.0650^**^ | 0.101^***^ | 0.00876 |
|  | (3.28) | (2.65) | (3.56) | (0.34) |
|  |  |  |  |  |
| Extraversion (Big 5) | 0.0292 | 0.000897 | 0.0287 | 0.0122 |
|  | (1.93) | (0.06) | (1.69) | (0.79) |
|  |  |  |  |  |
| Neuroticism (Big 5) | 0.0413^*^ | 0.0252 | 0.0283 | 0.0454^*^ |
|  | (2.39) | (1.49) | (1.46) | (2.57) |
|  |  |  |  |  |
| Conscientiousness (Big 5) | -0.0961^***^ | -0.0960^***^ | -0.124^***^ | -0.144^***^ |
|  | (-5.02) | (-5.12) | (-5.76) | (-7.34) |
|  |  |  |  |  |
| Compatibility (Big 5) | 0.0210 | 0.0109 | -0.0204 | -0.0138 |
|  | (1.07) | (0.57) | (-0.92) | (-0.68) |
|  |  |  |  |  |
| Openness (Big 5) | 0.0216 | 0.0121 | 0.0199 | 0.0259 |
|  | (1.37) | (0.78) | (1.12) | (1.61) |
|  |  |  |  |  |
| State (Reference: North Rhine-Westphalia) | | | | |
| Baden-Wuerttemberg | -0.00880 | 0.00307 | 0.00710 | -0.00938 |
|  | (-0.80) | (0.28) | (0.57) | (-0.83) |
|  |  |  |  |  |
| Bavaria | -0.0230 | 0.00683 | -0.00587 | 0.000690 |
|  | (-1.88) | (0.57) | (-0.43) | (0.06) |
|  |  |  |  |  |
| Berlin | 0.0228 | 0.0189 | -0.0113 | 0.0117 |
|  | (1.20) | (1.02) | (-0.53) | (0.60) |
|  |  |  |  |  |
| Brandenburg | -0.0444 | -0.0323 | -0.0377 | -0.0322 |
|  | (-1.77) | (-1.32) | (-1.34) | (-1.26) |
|  |  |  |  |  |
| Bremen | 0.00633 | 0.0174 | 0.00188 | 0.00280 |
|  | (0.35) | (0.98) | (0.09) | (0.15) |
|  |  |  |  |  |
| Hamburg | -0.0229 | 0.0111 | 0.0150 | 0.0321 |
|  | (-0.87) | (0.43) | (0.51) | (1.20) |
|  |  |  |  |  |
| Hesse | 0.0125 | 0.0303 | 0.0140 | -0.0197 |
|  | (0.76) | (1.90) | (0.76) | (-1.18) |
|  |  |  |  |  |
| Mecklenburg-Vorpommern | 0.00273 | -0.00735 | -0.0337 | -0.00696 |
|  | (0.09) | (-0.25) | (-1.00) | (-0.23) |
|  |  |  |  |  |
| Lower Saxony | -0.000890 | 0.0190 | 0.0106 | 0.000542 |
|  | (-0.06) | (1.37) | (0.66) | (0.04) |
|  |  |  |  |  |
| Rhineland Palatinate | -0.0318 | -0.00515 | -0.0363 | -0.0483^*^ |
|  | (-1.66) | (-0.27) | (-1.68) | (-2.45) |
|  |  |  |  |  |
| Saarland | 0.00721 | 0.0228 | 0.0275 | 0.0314 |
|  | (0.19) | (0.62) | (0.65) | (0.82) |
|  |  |  |  |  |
| Saxony | 0.0326 | 0.0298 | 0.0115 | 0.0284 |
|  | (1.61) | (1.50) | (0.51) | (1.37) |
|  |  |  |  |  |
| Saxony-Anhalt | -0.0491 | 0.0171 | -0.0758^*^ | -0.0301 |
|  | (-1.55) | (0.55) | (-2.13) | (-0.93) |
|  |  |  |  |  |
| Schleswig Holstein | -0.0164 | 0.00805 | 0.0251 | 0.0207 |
|  | (-0.78) | (0.39) | (1.06) | (0.96) |
|  |  |  |  |  |
| Thuringia | 0.00377 | 0.0270 | 0.0248 | 0.0470 |
|  | (0.16) | (1.14) | (0.91) | (1.89) |
|  |  |  |  |  |
| Foreign country | -0.0194 | -0.0194 | -0.0426 | -0.0405 |
|  | (-0.59) | (-0.60) | (-1.16) | (-1.21) |
|  |  |  |  |  |
| Gender (Reference: Male) |  |  |  |  |
| female | 0.0322^***^ | -0.00807 | 0.00371 | 0.0224^**^ |
|  | (4.05) | (-1.04) | (0.41) | (2.75) |
|  |  |  |  |  |
| non-binary | 0.0300 | 0.0319 | 0.0126 | 0.00316 |
|  | (0.67) | (0.73) | (0.25) | (0.07) |
|  |  |  |  |  |
| Age group (Reference: 18-30) | | | | |
| 31-45 | -0.0703^***^ | -0.0658^***^ | -0.0555^***^ | -0.0687^***^ |
|  | (-6.47) | (-6.18) | (-4.53) | (-6.17) |
|  |  |  |  |  |
| 46-60 | -0.0825^***^ | -0.102^***^ | -0.0994^***^ | -0.112^***^ |
|  | (-7.40) | (-9.34) | (-7.91) | (-9.80) |
|  |  |  |  |  |
| > 60 | -0.125^***^ | -0.151^***^ | -0.145^***^ | -0.144^***^ |
|  | (-10.14) | (-12.56) | (-10.47) | (-11.42) |
|  |  |  |  |  |
| Educational attainment (Reference: low) | | | | |
| middle | -0.0130 | -0.00846 | -0.00750 | -0.0123 |
|  | (-0.66) | (-0.44) | (-0.34) | (-0.60) |
|  |  |  |  |  |
| high | 0.00681 | 0.0119 | 0.0228 | -0.00878 |
|  | (0.36) | (0.64) | (1.07) | (-0.45) |
|  |  |  |  |  |
| Household income (Reference: I find it very difficult to make ends meet) | | | | |
| I find it somewhat difficult to make ends meet | 0.0247 | 0.00593 | 0.0374 | 0.0119 |
|  | (0.94) | (0.23) | (1.26) | (0.44) |
|  |  |  |  |  |
| I can make ends meet | 0.0327 | 0.0100 | 0.0426 | -0.00889 |
|  | (1.35) | (0.42) | (1.56) | (-0.36) |
|  |  |  |  |  |
| I can live somewhat comfortably on the income | 0.0279 | 0.00572 | 0.0424 | -0.0161 |
|  | (1.15) | (0.24) | (1.55) | (-0.65) |
|  |  |  |  |  |
| I can live very comfortably on the income | 0.0462 | 0.0168 | 0.0441 | -0.01000 |
|  | (1.82) | (0.67) | (1.54) | (-0.38) |
|  |  |  |  |  |
| does not apply (no own household income) | 0.00968 | -0.00915 | 0.0237 | -0.0306 |
|  | (0.25) | (-0.24) | (0.54) | (-0.76) |
|  |  |  |  |  |
| Health status | 0.0543^**^ | 0.0420^*^ | 0.0337 | 0.0294 |
|  | (2.94) | (2.32) | (1.62) | (1.55) |
|  |  |  |  |  |
| Constant | 0.0239 | 0.172^**^ | 0.136^*^ | 0.258^***^ |
|  | (0.42) | (3.12) | (2.13) | (4.47) |
| Observations | 6685 | 6685 | 6685 | 6685 |
| *R*^2^ | 0.134 | 0.147 | 0.168 | 0.110 |
| Adjusted *R*^2^ | 0.125 | 0.139 | 0.159 | 0.101 |

*Note*: T statistics in parentheses. * p < 0.05, ** p < 0.01, *** p < 0.001; GAL, green/alternative/liberal; TAN, traditional/authoritarian/nationalist.

## Supplementary Table 3: Outcome I - Cross check models A: concrete drivers for vaccination decision, multiple OLS regression (b-coefficients)

| Outcome I: Meaningfulness of … … as an incentive for vaccination | …free ticket for football match… | …free grilled sausage… | …vaccination lottery (chance to win big)… | …money… |
| --- | --- | --- | --- | --- |
| Frame (Reference: Control goup) | | | | |
| Long COVID frame 1 | 0.00801 | 0.00721 | 0.0335^*^ | 0.0309^*^ |
|  | (0.67) | (0.61) | (2.48) | (2.53) |
|  |  |  |  |  |
| Long COVID frame 2 | 0.00323 | 0.00436 | 0.0406^**^ | 0.0357^**^ |
|  | (0.27) | (0.37) | (3.03) | (2.94) |
|  |  |  |  |  |
| Economic frame 1 | -0.0260^*^ | -0.0159 | 0.0196 | 0.0277^*^ |
|  | (-2.18) | (-1.36) | (1.46) | (2.28) |
|  |  |  |  |  |
| Economic frame 2 | 0.00947 | 0.00694 | 0.0394^**^ | 0.0141 |
|  | (0.79) | (0.59) | (2.93) | (1.16) |
|  |  |  |  |  |
| Drivers of SARS-CoV-2 vaccination decision | | | | |
| Protecting self | 0.00540 | 0.00379 | 0.0410 | -0.0144 |
|  | (0.27) | (0.20) | (1.85) | (-0.72) |
|  |  |  |  |  |
| Protecting others | 0.0468^**^ | 0.0519^**^ | 0.0607^**^ | 0.0254 |
|  | (2.67) | (3.02) | (3.08) | (1.42) |
|  |  |  |  |  |
| Participation in public events | 0.0195 | -0.00605 | 0.0393^**^ | 0.0207 |
|  | (1.56) | (-0.49) | (2.79) | (1.62) |
|  |  |  |  |  |
| Vocational mandates | 0.000833 | -0.0321^**^ | -0.00907 | 0.00175 |
|  | (0.07) | (-2.91) | (-0.72) | (0.15) |
|  |  |  |  |  |
| Medical advice/recommendation of the Permanent Vaccination Commission (STIKO) | 0.0189 | 0.00781 | 0.0150 | -0.00775 |
|  | (1.35) | (0.57) | (0.96) | (-0.55) |
|  |  |  |  |  |
| Peer pressure | 0.0370^*^ | 0.0312^*^ | 0.0450^**^ | 0.0442^**^ |
|  | (2.52) | (2.17) | (2.73) | (2.96) |
|  |  |  |  |  |
| Number of COVID-19 vaccinations received (Reference: one vaccination received) | | | | |
| two vaccinations received | 0.0107 | -0.00175 | -0.0154 | 0.00438 |
|  | (0.26) | (-0.04) | (-0.33) | (0.10) |
|  |  |  |  |  |
| three vaccinations received | 0.0248 | 0.00848 | 0.0115 | 0.0267 |
|  | (0.60) | (0.21) | (0.25) | (0.64) |
|  |  |  |  |  |
| four vaccinations received | 0.0646 | 0.0372 | 0.0426 | 0.0194 |
|  | (1.50) | (0.88) | (0.88) | (0.44) |
|  |  |  |  |  |
| Immunization decision was very difficult and emotionally highly stressful for me. | -0.0302 | -0.0422^*^ | -0.0703^**^ | -0.0363 |
|  | (-1.44) | (-2.05) | (-2.99) | (-1.70) |
|  |  |  |  |  |
| Immunization decision didn't take long for me. | 0.0514^**^ | 0.0416^**^ | 0.0184 | 0.0237 |
|  | (3.16) | (2.61) | (1.01) | (1.43) |
|  |  |  |  |  |
| How threatening? COVID-19 | 0.0792^***^ | 0.0853^***^ | 0.0714^***^ | 0.0867^***^ |
|  | (4.61) | (5.07) | (3.70) | (4.97) |
|  |  |  |  |  |
| Current COVID-19 safety perception | 0.0149 | 0.00974 | -0.0216 | 0.00338 |
|  | (0.80) | (0.54) | (-1.04) | (0.18) |
|  |  |  |  |  |
| Waldorf education | 0.00225 | -0.0150 | 0.0298 | 0.00110 |
|  | (0.13) | (-0.91) | (1.58) | (0.06) |
|  |  |  |  |  |
| Homeopathy | -0.0343^*^ | -0.0590^***^ | -0.0831^***^ | -0.0436^**^ |
|  | (-2.26) | (-3.96) | (-4.86) | (-2.82) |
|  |  |  |  |  |
| Mainstream medicine | 0.0136 | 0.0464^*^ | 0.0547^*^ | 0.000165 |
|  | (0.59) | (2.06) | (2.12) | (0.01) |
|  |  |  |  |  |
| Religious denomination (Reference: no denomination) | | | | |
| Roman-Catholic | 0.0103 | -0.00458 | -0.0126 | -0.00682 |
|  | (0.97) | (-0.44) | (-1.05) | (-0.63) |
|  |  |  |  |  |
| Protestant | 0.0305^**^ | 0.0203 | 0.00954 | 0.00829 |
|  | (2.82) | (1.92) | (0.78) | (0.75) |
|  |  |  |  |  |
| Evangelical Free Church | -0.00195 | -0.00235 | -0.0232 | 0.0158 |
|  | (-0.14) | (-0.17) | (-1.45) | (1.09) |
|  |  |  |  |  |
| Orthodox Christian | -0.0565 | -0.0124 | 0.138 | 0.0779 |
|  | (-0.67) | (-0.15) | (1.45) | (0.90) |
|  |  |  |  |  |
| Jewish | 0.169 | 0.160 | 0.0286 | -0.0636 |
|  | (1.82) | (1.75) | (0.27) | (-0.67) |
|  |  |  |  |  |
| Muslim | -0.00571 | -0.147^**^ | 0.0436 | -0.0431 |
|  | (-0.10) | (-2.59) | (0.67) | (-0.73) |
|  |  |  |  |  |
| Other | 0.0271 | 0.0371 | -0.00674 | -0.000282 |
|  | (0.97) | (1.35) | (-0.21) | (-0.01) |
|  |  |  |  |  |
| Left = 0/Right = 1 | -0.0104 | -0.0273 | 0.0577 | -0.0559 |
|  | (-0.34) | (-0.90) | (1.66) | (-1.78) |
|  |  |  |  |  |
| GAL = 0/TAN = 1 | -0.00939 | -0.0180 | -0.0848^**^ | -0.0281 |
|  | (-0.39) | (-0.76) | (-3.14) | (-1.15) |
|  |  |  |  |  |
| Voting Intention (Reference: CDU/CSU: Christian democrats) | | | | |
| SPD (Social democrats) | 0.0363^*^ | 0.0116 | 0.0398^*^ | 0.0114 |
|  | (2.29) | (0.75) | (2.24) | (0.71) |
|  |  |  |  |  |
| Greens | 0.0407^**^ | 0.0149 | 0.0631^***^ | 0.0331^*^ |
|  | (2.89) | (1.08) | (3.99) | (2.32) |
|  |  |  |  |  |
| FDP (Liberals) | 0.0343^*^ | 0.0139 | 0.0408^*^ | 0.0191 |
|  | (2.06) | (0.85) | (2.18) | (1.13) |
|  |  |  |  |  |
| Left-Party (Socialist) | 0.0292 | -0.0186 | 0.0231 | 0.0240 |
|  | (1.42) | (-0.92) | (1.00) | (1.14) |
|  |  |  |  |  |
| AfD (Populist right wing) | -0.0000949 | -0.0122 | 0.0111 | 0.0257 |
|  | (-0.00) | (-0.58) | (0.47) | (1.19) |
|  |  |  |  |  |
| Other | 0.0118 | -0.00737 | 0.0217 | 0.0103 |
|  | (0.76) | (-0.49) | (1.25) | (0.66) |
|  |  |  |  |  |
| Perception of social division | -0.112^***^ | -0.127^***^ | -0.121^***^ | -0.0788^**^ |
|  | (-4.02) | (-4.65) | (-3.89) | (-2.79) |
|  |  |  |  |  |
| Solidarity | 0.0695^*^ | 0.0457 | 0.0877^**^ | -0.00378 |
|  | (2.46) | (1.65) | (2.76) | (-0.13) |
|  |  |  |  |  |
| Extraversion (Big 5) | 0.0228 | 0.000854 | 0.0213 | 0.0107 |
|  | (1.37) | (0.05) | (1.14) | (0.63) |
|  |  |  |  |  |
| Neuroticism (Big 5) | 0.0403^*^ | 0.0239 | 0.0207 | 0.0396^*^ |
|  | (2.15) | (1.30) | (0.98) | (2.07) |
|  |  |  |  |  |
| Conscientiousness (Big 5) | -0.106^***^ | -0.102^***^ | -0.137^***^ | -0.152^***^ |
|  | (-5.06) | (-4.96) | (-5.78) | (-7.12) |
|  |  |  |  |  |
| Compatibility (Big 5) | 0.0143 | 0.00431 | -0.0244 | -0.0213 |
|  | (0.66) | (0.20) | (-1.01) | (-0.97) |
|  |  |  |  |  |
| Openness (Big 5) | 0.0190 | 0.0104 | 0.0192 | 0.0278 |
|  | (1.11) | (0.62) | (1.00) | (1.59) |
|  |  |  |  |  |
| State (Reference: North Rhine-Westphalia) | | | | |
| Baden-Wuerttemberg | -0.0120 | 0.000982 | 0.00520 | -0.0123 |
|  | (-1.00) | (0.08) | (0.39) | (-1.01) |
|  |  |  |  |  |
| Bavaria | -0.0243 | 0.00747 | -0.00613 | 0.00106 |
|  | (-1.83) | (0.57) | (-0.41) | (0.08) |
|  |  |  |  |  |
| Berlin | 0.0238 | 0.0184 | -0.0177 | 0.00850 |
|  | (1.14) | (0.90) | (-0.75) | (0.40) |
|  |  |  |  |  |
| Brandenburg | -0.0468 | -0.0348 | -0.0384 | -0.0327 |
|  | (-1.72) | (-1.31) | (-1.26) | (-1.18) |
|  |  |  |  |  |
| Bremen | 0.00483 | 0.0153 | -0.00438 | 0.00670 |
|  | (0.25) | (0.81) | (-0.20) | (0.34) |
|  |  |  |  |  |
| Hamburg | -0.0281 | 0.0124 | 0.00507 | 0.0365 |
|  | (-1.00) | (0.45) | (0.16) | (1.28) |
|  |  |  |  |  |
| Hesse | 0.0109 | 0.0307 | 0.0110 | -0.0221 |
|  | (0.61) | (1.76) | (0.55) | (-1.22) |
|  |  |  |  |  |
| Mecklenburg-Vorpommern | -0.00511 | -0.0149 | -0.0493 | -0.0283 |
|  | (-0.15) | (-0.45) | (-1.30) | (-0.83) |
|  |  |  |  |  |
| Lower Saxony | -0.000915 | 0.0225 | 0.0105 | -0.00105 |
|  | (-0.06) | (1.49) | (0.61) | (-0.07) |
|  |  |  |  |  |
| Rhineland Palatinate | -0.0281 | -0.00456 | -0.0368 | -0.0503^*^ |
|  | (-1.35) | (-0.22) | (-1.57) | (-2.38) |
|  |  |  |  |  |
| Saarland | -0.00302 | 0.00933 | 0.0212 | 0.0263 |
|  | (-0.07) | (0.23) | (0.46) | (0.63) |
|  |  |  |  |  |
| Saxony | 0.0324 | 0.0352 | 0.00365 | 0.0207 |
|  | (1.42) | (1.57) | (0.14) | (0.89) |
|  |  |  |  |  |
| Saxony-Anhalt | -0.0682 | 0.00858 | -0.106^**^ | -0.0515 |
|  | (-1.93) | (0.25) | (-2.67) | (-1.43) |
|  |  |  |  |  |
| Schleswig Holstein | -0.0179 | 0.00960 | 0.0262 | 0.0233 |
|  | (-0.79) | (0.43) | (1.03) | (1.01) |
|  |  |  |  |  |
| Thuringia | 0.00351 | 0.0296 | 0.0296 | 0.0533 |
|  | (0.13) | (1.13) | (0.98) | (1.96) |
|  |  |  |  |  |
| Foreign country | -0.0266 | -0.0266 | -0.0401 | -0.0424 |
|  | (-0.71) | (-0.73) | (-0.96) | (-1.12) |
|  |  |  |  |  |
| Gender (Reference: male) |  |  |  |  |
| female | 0.0350^***^ | -0.0110 | 0.00292 | 0.0262^**^ |
|  | (4.02) | (-1.29) | (0.30) | (2.96) |
|  |  |  |  |  |
| non-binary | 0.0414 | 0.0396 | 0.0289 | -0.00438 |
|  | (0.83) | (0.81) | (0.52) | (-0.09) |
|  |  |  |  |  |
| Age group (Reference: 18-30) |  |  |  |  |
| 31-45 | -0.0660^***^ | -0.0585^***^ | -0.0487^***^ | -0.0648^***^ |
|  | (-5.63) | (-5.09) | (-3.69) | (-5.43) |
|  |  |  |  |  |
| 46-60 | -0.0797^***^ | -0.0964^***^ | -0.0946^***^ | -0.111^***^ |
|  | (-6.55) | (-8.08) | (-6.92) | (-8.99) |
|  |  |  |  |  |
| > 60 | -0.127^***^ | -0.152^***^ | -0.146^***^ | -0.147^***^ |
|  | (-9.52) | (-11.58) | (-9.72) | (-10.83) |
|  |  |  |  |  |
| Educational attainment (Reference: low) | | | | |
| middle | 0.000257 | 0.00756 | 0.0110 | 0.00170 |
|  | (0.01) | (0.36) | (0.45) | (0.08) |
|  |  |  |  |  |
| high | 0.0215 | 0.0286 | 0.0432 | 0.00574 |
|  | (1.04) | (1.41) | (1.86) | (0.27) |
|  |  |  |  |  |
| Household income (Reference: I find it very difficult to make ends meet) | | | | |
| I find it somewhat difficult to make ends meet | 0.0222 | 0.000301 | 0.0373 | -0.00394 |
|  | (0.73) | (0.01) | (1.09) | (-0.13) |
|  |  |  |  |  |
| I can make ends meet | 0.0350 | 0.0131 | 0.0480 | -0.0229 |
|  | (1.24) | (0.47) | (1.51) | (-0.80) |
|  |  |  |  |  |
| I can live somewhat comfortably on the income | 0.0288 | 0.00842 | 0.0465 | -0.0301 |
|  | (1.02) | (0.30) | (1.47) | (-1.05) |
|  |  |  |  |  |
| I can live very comfortably on the income | 0.0481 | 0.0178 | 0.0474 | -0.0230 |
|  | (1.64) | (0.62) | (1.44) | (-0.77) |
|  |  |  |  |  |
| does not apply (no own household income) | 0.0119 | -0.0172 | 0.0171 | -0.0551 |
|  | (0.27) | (-0.40) | (0.35) | (-1.25) |
|  |  |  |  |  |
| Health status | 0.0597^**^ | 0.0497^*^ | 0.0356 | 0.0319 |
|  | (2.96) | (2.51) | (1.57) | (1.56) |
|  |  |  |  |  |
| Constant | 0.0569 | 0.209^**^ | 0.163^*^ | 0.333^***^ |
|  | (0.78) | (2.94) | (2.00) | (4.50) |
| Observations | 6028 | 6028 | 6028 | 6028 |
| *R*^2^ | 0.107 | 0.123 | 0.130 | 0.094 |
| Adjusted *R*^2^ | 0.097 | 0.113 | 0.120 | 0.084 |

*Note:* T statistics in parentheses. ^*^ *p* < 0.05, ^**^ *p* < 0.01, ^***^ *p* < 0.001; GAL, green/alternative/liberal; TAN, traditional/authoritarian/nationalist.

## Supplementary Table 4: Outcome I - Cross check models B: self perceived knowledge about COVID-19 vaccination, multiple OLS regression (b-coefficients)

| Outcome I: Meaningfulness of … … as an incentive for vaccination | …free ticket for football match… | …free grilled sausage… | …vaccination lottery (chance to win big)… | …money… |
| --- | --- | --- | --- | --- |
| Frame (Reference: Control group) | | | | |
| Long COVID frame 1 | 0.0104 | 0.00828 | 0.0340^*^ | 0.0337^**^ |
|  | (0.88) | (0.72) | (2.55) | (2.81) |
|  |  |  |  |  |
| Long COVID frame 2 | 0.0105 | 0.0121 | 0.0447^***^ | 0.0381^**^ |
|  | (0.90) | (1.05) | (3.37) | (3.20) |
|  |  |  |  |  |
| Economic frame 1 | -0.0261^*^ | -0.0145 | 0.0215 | 0.0306^*^ |
|  | (-2.22) | (-1.26) | (1.62) | (2.56) |
|  |  |  |  |  |
| Economic frame 2 | 0.0214 | 0.0122 | 0.0499^***^ | 0.0216 |
|  | (1.80) | (1.05) | (3.72) | (1.79) |
|  |  |  |  |  |
| Self perception knowledge COVID-19 vaccination | 0.0516^**^ | 0.0724^***^ | 0.0869^***^ | 0.00939 |
|  | (2.65) | (3.81) | (3.95) | (0.47) |
|  |  |  |  |  |
| Number of COVID-19 vaccinations received (Reference: not vaccinated) | | | | |
| one vaccination received | 0.0361 | 0.0305 | 0.0903 | 0.0535 |
|  | (0.80) | (0.69) | (1.77) | (1.17) |
|  |  |  |  |  |
| two vaccinations received | 0.0835^***^ | 0.0702^***^ | 0.107^***^ | 0.0551^*^ |
|  | (3.95) | (3.39) | (4.50) | (2.57) |
|  |  |  |  |  |
| three vaccinations received | 0.111^***^ | 0.0873^***^ | 0.153^***^ | 0.0999^***^ |
|  | (6.57) | (5.27) | (8.03) | (5.81) |
|  |  |  |  |  |
| four vaccinations received | 0.141^***^ | 0.110^***^ | 0.187^***^ | 0.0922^***^ |
|  | (6.79) | (5.45) | (7.99) | (4.39) |
|  |  |  |  |  |
| Immunization decision was very difficult and emotionally highly stressful for me. | -0.0341 | -0.0520^**^ | -0.0809^***^ | -0.0279 |
|  | (-1.90) | (-2.96) | (-3.99) | (-1.53) |
|  |  |  |  |  |
| Immunization decision didn't take long for me. | 0.0470^**^ | 0.0423^**^ | 0.0248 | 0.0165 |
|  | (3.12) | (2.88) | (1.46) | (1.08) |
|  |  |  |  |  |
| How threatening? COVID-19 | 0.0888^***^ | 0.0885^***^ | 0.0788^***^ | 0.0781^***^ |
|  | (5.47) | (5.58) | (4.31) | (4.75) |
|  |  |  |  |  |
| Current COVID-19 safety perception | -0.000335 | -0.00284 | -0.0420^*^ | -0.0121 |
|  | (-0.02) | (-0.16) | (-2.01) | (-0.64) |
|  |  |  |  |  |
| Waldorf education | -0.000968 | -0.0178 | 0.0235 | -0.00849 |
|  | (-0.06) | (-1.10) | (1.26) | (-0.50) |
|  |  |  |  |  |
| Homeopathy | -0.0277 | -0.0476^**^ | -0.0639^***^ | -0.0381^*^ |
|  | (-1.83) | (-3.23) | (-3.75) | (-2.48) |
|  |  |  |  |  |
| Mainstream medicine | 0.0342 | 0.0554^**^ | 0.0695^**^ | -0.00432 |
|  | (1.58) | (2.63) | (2.85) | (-0.20) |
|  |  |  |  |  |
| Religious denomination (Reference: no denomination) | | | | |
| Roman-Catholic | 0.0122 | -0.00349 | -0.00560 | -0.00665 |
|  | (1.15) | (-0.34) | (-0.47) | (-0.62) |
|  |  |  |  |  |
| Protestant | 0.0271^*^ | 0.0152 | 0.00675 | 0.00952 |
|  | (2.54) | (1.46) | (0.56) | (0.88) |
|  |  |  |  |  |
| Evangelical Free Church | -0.000501 | -0.00489 | -0.0151 | 0.0196 |
|  | (-0.04) | (-0.35) | (-0.94) | (1.36) |
|  |  |  |  |  |
| Orthodox Christian | -0.0869 | 0.0231 | 0.0683 | 0.0404 |
|  | (-1.15) | (0.31) | (0.80) | (0.53) |
|  |  |  |  |  |
| Jewish | 0.215^*^ | 0.194^*^ | 0.0205 | -0.0347 |
|  | (2.27) | (2.10) | (0.19) | (-0.36) |
|  |  |  |  |  |
| Muslim | 0.00310 | -0.107 | 0.0228 | 0.0172 |
|  | (0.05) | (-1.80) | (0.33) | (0.28) |
|  |  |  |  |  |
| Other | 0.0288 | 0.0273 | -0.0115 | 0.00269 |
|  | (1.04) | (1.01) | (-0.37) | (0.10) |
|  |  |  |  |  |
| Left = 0/Right = 1 | -0.0253 | -0.0382 | 0.0364 | -0.0755^*^ |
|  | (-0.84) | (-1.31) | (1.08) | (-2.49) |
|  |  |  |  |  |
| GAL = 0/TAN = 1 | -0.00511 | -0.00294 | -0.0776^**^ | -0.00478 |
|  | (-0.22) | (-0.13) | (-2.92) | (-0.20) |
|  |  |  |  |  |
| Voting Intention (Reference: CDU/CSU: Christian democrats) | | | | |
| SPD (Social democrats) | 0.0405^*^ | 0.0203 | 0.0414^*^ | 0.0127 |
|  | (2.53) | (1.30) | (2.29) | (0.78) |
|  |  |  |  |  |
| Greens | 0.0407^**^ | 0.0236 | 0.0603^***^ | 0.0312^*^ |
|  | (2.91) | (1.73) | (3.82) | (2.20) |
|  |  |  |  |  |
| FDP (Liberals) | 0.0435^**^ | 0.0188 | 0.0551^**^ | 0.0128 |
|  | (2.58) | (1.14) | (2.90) | (0.75) |
|  |  |  |  |  |
| Left-Party (Socialist) | 0.0322 | -0.0177 | 0.0218 | 0.0119 |
|  | (1.57) | (-0.88) | (0.94) | (0.57) |
|  |  |  |  |  |
| AfD (Populist right wing) | -0.0102 | -0.0322 | -0.0130 | 0.00890 |
|  | (-0.53) | (-1.71) | (-0.60) | (0.45) |
|  |  |  |  |  |
| Other | 0.00842 | -0.0110 | 0.00729 | -0.00205 |
|  | (0.55) | (-0.74) | (0.42) | (-0.13) |
|  |  |  |  |  |
| Perception of social division | -0.105^***^ | -0.134^***^ | -0.116^***^ | -0.0701^*^ |
|  | (-3.86) | (-5.02) | (-3.77) | (-2.53) |
|  |  |  |  |  |
| Solidarity | 0.0762^**^ | 0.0638^*^ | 0.100^**^ | 0.0111 |
|  | (2.80) | (2.40) | (3.26) | (0.40) |
|  |  |  |  |  |
| Extraversion (Big 5) | 0.0396^*^ | 0.00334 | 0.0266 | 0.0177 |
|  | (2.42) | (0.21) | (1.44) | (1.07) |
|  |  |  |  |  |
| Neuroticism (Big 5) | 0.0384^*^ | 0.0259 | 0.0332 | 0.0493^**^ |
|  | (2.05) | (1.41) | (1.57) | (2.59) |
|  |  |  |  |  |
| Conscientiousness (Big 5) | -0.0978^***^ | -0.0971^***^ | -0.138^***^ | -0.143^***^ |
|  | (-4.74) | (-4.82) | (-5.93) | (-6.85) |
|  |  |  |  |  |
| Compatibility (Big 5) | 0.0163 | 0.00393 | -0.00508 | -0.000514 |
|  | (0.76) | (0.19) | (-0.21) | (-0.02) |
|  |  |  |  |  |
| Openness (Big 5) | 0.0193 | 0.0103 | 0.0147 | 0.0258 |
|  | (1.12) | (0.62) | (0.76) | (1.49) |
|  |  |  |  |  |
| State (Reference: North Rhine-Westphalia) | | | | |
| Baden-Wuerttemberg | -0.0110 | 0.00552 | 0.00891 | -0.0102 |
|  | (-0.93) | (0.48) | (0.67) | (-0.85) |
|  |  |  |  |  |
| Bavaria | -0.0249 | 0.00796 | -0.00376 | -0.00404 |
|  | (-1.89) | (0.62) | (-0.25) | (-0.30) |
|  |  |  |  |  |
| Berlin | 0.0104 | 0.0207 | -0.0150 | 0.0126 |
|  | (0.50) | (1.02) | (-0.64) | (0.60) |
|  |  |  |  |  |
| Brandenburg | -0.0487 | -0.0393 | -0.0262 | -0.0352 |
|  | (-1.79) | (-1.48) | (-0.85) | (-1.27) |
|  |  |  |  |  |
| Bremen | 0.00926 | 0.0211 | -0.000430 | -0.00181 |
|  | (0.49) | (1.14) | (-0.02) | (-0.09) |
|  |  |  |  |  |
| Hamburg | -0.0315 | 0.0118 | 0.0143 | 0.0276 |
|  | (-1.09) | (0.42) | (0.44) | (0.94) |
|  |  |  |  |  |
| Hesse | 0.00184 | 0.0170 | 0.00697 | -0.0375^*^ |
|  | (0.10) | (0.99) | (0.35) | (-2.09) |
|  |  |  |  |  |
| Mecklenburg-Vorpommern | 0.00521 | -0.0158 | -0.0223 | -0.00860 |
|  | (0.16) | (-0.50) | (-0.62) | (-0.26) |
|  |  |  |  |  |
| Lower Saxony | -0.00761 | 0.0144 | 0.000314 | -0.0101 |
|  | (-0.50) | (0.97) | (0.02) | (-0.65) |
|  |  |  |  |  |
| Rhineland Palatinate | -0.0500^*^ | -0.0209 | -0.0522^*^ | -0.0647^**^ |
|  | (-2.42) | (-1.04) | (-2.25) | (-3.10) |
|  |  |  |  |  |
| Saarland | 0.0398 | 0.0610 | 0.0463 | 0.0649 |
|  | (0.98) | (1.54) | (1.01) | (1.58) |
|  |  |  |  |  |
| Saxony | 0.0480^*^ | 0.0378 | 0.0160 | 0.0434 |
|  | (2.20) | (1.77) | (0.65) | (1.96) |
|  |  |  |  |  |
| Saxony-Anhalt | -0.0304 | 0.0559 | -0.0655 | -0.00366 |
|  | (-0.83) | (1.57) | (-1.59) | (-0.10) |
|  |  |  |  |  |
| Schleswig Holstein | -0.0145 | -0.00212 | 0.0206 | 0.000146 |
|  | (-0.65) | (-0.10) | (0.82) | (0.01) |
|  |  |  |  |  |
| Thuringia | 0.000963 | 0.0245 | 0.0310 | 0.0558^*^ |
|  | (0.04) | (0.97) | (1.06) | (2.12) |
|  |  |  |  |  |
| Foreign country | -0.0287 | -0.0353 | -0.0482 | -0.0398 |
|  | (-0.79) | (-1.00) | (-1.18) | (-1.08) |
|  |  |  |  |  |
| Gender (Reference: male) |  |  |  |  |
| female | 0.0277^**^ | -0.0104 | 0.00291 | 0.0153 |
|  | (3.28) | (-1.25) | (0.31) | (1.78) |
|  |  |  |  |  |
| non-binary | 0.0288 | 0.0121 | -0.00770 | -0.0280 |
|  | (0.59) | (0.25) | (-0.14) | (-0.57) |
|  |  |  |  |  |
| Age group (Reference: 18-30) |  |  |  |  |
| 31-45 | -0.0689^***^ | -0.0535^***^ | -0.0525^***^ | -0.0695^***^ |
|  | (-5.74) | (-4.57) | (-3.88) | (-5.71) |
|  |  |  |  |  |
| 46-60 | -0.0817^***^ | -0.0929^***^ | -0.0963^***^ | -0.115^***^ |
|  | (-6.66) | (-7.76) | (-6.97) | (-9.23) |
|  |  |  |  |  |
| > 60 | -0.125^***^ | -0.146^***^ | -0.147^***^ | -0.149^***^ |
|  | (-9.36) | (-11.21) | (-9.74) | (-11.01) |
|  |  |  |  |  |
| Educational attainment (Reference: low) | | | | |
| middle | -0.0119 | -0.00677 | -0.00732 | -0.0114 |
|  | (-0.56) | (-0.32) | (-0.30) | (-0.53) |
|  |  |  |  |  |
| high | 0.00715 | 0.0128 | 0.0210 | -0.00747 |
|  | (0.35) | (0.64) | (0.91) | (-0.36) |
|  |  |  |  |  |
| Household income (Reference: I find it very difficult to make ends meet) | | | | |
| I find it somewhat difficult to make ends meet | 0.0173 | -0.0128 | 0.0105 | 0.0178 |
|  | (0.58) | (-0.44) | (0.31) | (0.59) |
|  |  |  |  |  |
| I can make ends meet | 0.0339 | -0.00383 | 0.0174 | -0.000812 |
|  | (1.24) | (-0.14) | (0.56) | (-0.03) |
|  |  |  |  |  |
| I can live somewhat comfortably on the income | 0.0303 | -0.00799 | 0.0169 | -0.00360 |
|  | (1.10) | (-0.30) | (0.55) | (-0.13) |
|  |  |  |  |  |
| I can live very comfortably on the income | 0.0490 | -0.000432 | 0.0160 | 0.00285 |
|  | (1.72) | (-0.02) | (0.50) | (0.10) |
|  |  |  |  |  |
| does not apply (no own household income) | 0.0441 | -0.0410 | 0.0156 | -0.0640 |
|  | (0.98) | (-0.94) | (0.31) | (-1.41) |
|  |  |  |  |  |
| Health status | 0.0448^*^ | 0.0253 | 0.0323 | 0.0403^*^ |
|  | (2.25) | (1.30) | (1.44) | (1.99) |
|  |  |  |  |  |
| Constant | 0.00582 | 0.146^*^ | 0.107 | 0.255^***^ |
|  | (0.09) | (2.35) | (1.50) | (3.96) |
| Observations | 5764 | 5764 | 5764 | 5764 |
| *R*^2^ | 0.135 | 0.147 | 0.170 | 0.116 |
| Adjusted *R*^2^ | 0.125 | 0.137 | 0.160 | 0.106 |

*Note:* T statistics in parentheses. ^*^ *p* < 0.05, ^**^ *p* < 0.01, ^***^ *p* < 0.001; GAL, green/alternative/liberal; TAN, traditional/authoritarian/nationalist.

## Supplementary Table 5: Outcome I - Cross check models C: private health insurance, multiple OLS regression (b-coefficients)

| Outcome I: Meaningfulness of … … as an incentive for vaccination | …free ticket for football match… | …free grilled sausage… | …vaccination lottery (chance to win big)… | …money… |
| --- | --- | --- | --- | --- |
| Frame (Reference: Control group) | | | | |
| Long COVID frame 1 | 0.00748 | 0.00664 | 0.0297^*^ | 0.0290^*^ |
|  | (0.68) | (0.62) | (2.39) | (2.57) |
|  |  |  |  |  |
| Long COVID frame 2 | 0.00234 | 0.00585 | 0.0375^**^ | 0.0326^**^ |
|  | (0.21) | (0.54) | (3.03) | (2.90) |
|  |  |  |  |  |
| Economic frame 1 | -0.0226^*^ | -0.0135 | 0.0192 | 0.0289^*^ |
|  | (-2.07) | (-1.26) | (1.56) | (2.57) |
|  |  |  |  |  |
| Economic frame 2 | 0.0128 | 0.00985 | 0.0428^***^ | 0.0170 |
|  | (1.16) | (0.91) | (3.44) | (1.50) |
|  |  |  |  |  |
| Private health insurance | -0.00630 | -0.00622 | -0.000183 | 0.00318 |
|  | (-0.74) | (-0.74) | (-0.02) | (0.36) |
|  |  |  |  |  |
| Number of COVID-19 vaccinations received (Reference: not vaccinated) | | | | |
| one vaccination received | 0.0552 | 0.0489 | 0.0818 | 0.0597 |
|  | (1.37) | (1.24) | (1.80) | (1.44) |
|  |  |  |  |  |
| two vaccinations received | 0.0784^***^ | 0.0577^**^ | 0.0880^***^ | 0.0703^***^ |
|  | (4.08) | (3.07) | (4.06) | (3.57) |
|  |  |  |  |  |
| three vaccinations received | 0.105^***^ | 0.0763^***^ | 0.135^***^ | 0.0979^***^ |
|  | (6.74) | (5.01) | (7.71) | (6.14) |
|  |  |  |  |  |
| four vaccinations received | 0.143^***^ | 0.106^***^ | 0.168^***^ | 0.0870^***^ |
|  | (7.35) | (5.56) | (7.68) | (4.38) |
|  |  |  |  |  |
| Immunization decision was very difficult and emotionally highly stressful for me. | -0.0345^*^ | -0.0520^**^ | -0.0735^***^ | -0.0215 |
|  | (-2.05) | (-3.15) | (-3.87) | (-1.25) |
|  |  |  |  |  |
| Immunization decision didn't take long for me. | 0.0487^***^ | 0.0424^**^ | 0.0230 | 0.0178 |
|  | (3.46) | (3.07) | (1.45) | (1.23) |
|  |  |  |  |  |
| How threatening? COVID-19 | 0.0878^***^ | 0.0946^***^ | 0.0895^***^ | 0.0812^***^ |
|  | (5.86) | (6.46) | (5.31) | (5.30) |
|  |  |  |  |  |
| Current COVID-19 safety perception | 0.0124 | 0.00761 | -0.0249 | -0.00106 |
|  | (0.73) | (0.45) | (-1.29) | (-0.06) |
|  |  |  |  |  |
| Waldorf education | 0.00553 | -0.0133 | 0.0309 | 0.00345 |
|  | (0.36) | (-0.88) | (1.78) | (0.22) |
|  |  |  |  |  |
| Homeopathy | -0.0351^*^ | -0.0620^***^ | -0.0832^***^ | -0.0450^**^ |
|  | (-2.52) | (-4.55) | (-5.30) | (-3.15) |
|  |  |  |  |  |
| Mainstream medicine | 0.0325 | 0.0537^**^ | 0.0742^***^ | 0.0141 |
|  | (1.65) | (2.78) | (3.34) | (0.70) |
|  |  |  |  |  |
| Religious denomination (Reference: no denomination) | | | | |
| Roman-Catholic | 0.0114 | -0.00634 | -0.00759 | -0.00654 |
|  | (1.16) | (-0.66) | (-0.69) | (-0.65) |
|  |  |  |  |  |
| Protestant | 0.0292^**^ | 0.0187 | 0.0102 | 0.00683 |
|  | (2.90) | (1.91) | (0.90) | (0.66) |
|  |  |  |  |  |
| Evangelical Free Church | -0.00161 | -0.00644 | -0.0217 | 0.0152 |
|  | (-0.12) | (-0.50) | (-1.47) | (1.14) |
|  |  |  |  |  |
| Orthodox Christian | -0.0423 | -0.00955 | 0.0933 | 0.0422 |
|  | (-0.61) | (-0.14) | (1.20) | (0.60) |
|  |  |  |  |  |
| Jewish | 0.158 | 0.143 | 0.0239 | -0.0548 |
|  | (1.85) | (1.71) | (0.25) | (-0.62) |
|  |  |  |  |  |
| Muslim | 0.0185 | -0.128^*^ | 0.0560 | -0.0192 |
|  | (0.36) | (-2.51) | (0.95) | (-0.36) |
|  |  |  |  |  |
| Other | 0.0234 | 0.0322 | -0.00380 | 0.00140 |
|  | (0.93) | (1.31) | (-0.13) | (0.05) |
|  |  |  |  |  |
| Left = 0/Right = 1 | -0.00620 | -0.0183 | 0.0514 | -0.0389 |
|  | (-0.23) | (-0.68) | (1.66) | (-1.38) |
|  |  |  |  |  |
| GAL = 0/TAN = 1 | -0.0107 | -0.0224 | -0.0818^***^ | -0.0273 |
|  | (-0.49) | (-1.06) | (-3.36) | (-1.23) |
|  |  |  |  |  |
| Voting Intentions (Reference: CDU/CSU: Christian democrats) | | | | |
| SPD (Social democrats) | 0.0361^*^ | 0.0142 | 0.0426^*^ | 0.0155 |
|  | (2.42) | (0.97) | (2.54) | (1.02) |
|  |  |  |  |  |
| Greens | 0.0416^**^ | 0.0189 | 0.0645^***^ | 0.0334^*^ |
|  | (3.16) | (1.47) | (4.35) | (2.48) |
|  |  |  |  |  |
| FDP (Liberals) | 0.0322^*^ | 0.0109 | 0.0406^*^ | 0.0196 |
|  | (2.08) | (0.72) | (2.32) | (1.23) |
|  |  |  |  |  |
| Left-Party (Socialist) | 0.0243 | -0.0195 | 0.0154 | 0.0217 |
|  | (1.28) | (-1.05) | (0.72) | (1.11) |
|  |  |  |  |  |
| AfD (Populist right wing) | -0.0147 | -0.0282 | -0.0112 | 0.0116 |
|  | (-0.83) | (-1.63) | (-0.56) | (0.64) |
|  |  |  |  |  |
| Other | 0.00353 | -0.00981 | 0.0104 | 0.00561 |
|  | (0.25) | (-0.71) | (0.65) | (0.39) |
|  |  |  |  |  |
| Perception of social division | -0.102^***^ | -0.121^***^ | -0.119^***^ | -0.0737^**^ |
|  | (-4.00) | (-4.88) | (-4.17) | (-2.83) |
|  |  |  |  |  |
| Solidarity | 0.0828^***^ | 0.0656^**^ | 0.101^***^ | 0.00907 |
|  | (3.30) | (2.67) | (3.59) | (0.35) |
|  |  |  |  |  |
| Extraversion (Big 5) | 0.0293 | 0.000668 | 0.0283 | 0.0120 |
|  | (1.94) | (0.05) | (1.66) | (0.78) |
|  |  |  |  |  |
| Neuroticism (Big 5) | 0.0409^*^ | 0.0254 | 0.0285 | 0.0453^*^ |
|  | (2.37) | (1.50) | (1.47) | (2.57) |
|  |  |  |  |  |
| Conscientiousness (Big 5) | -0.0960^***^ | -0.0958^***^ | -0.124^***^ | -0.144^***^ |
|  | (-5.01) | (-5.10) | (-5.74) | (-7.33) |
|  |  |  |  |  |
| Compatibility (Big 5) | 0.0208 | 0.0106 | -0.0211 | -0.0143 |
|  | (1.06) | (0.55) | (-0.95) | (-0.71) |
|  |  |  |  |  |
| Openness (Big 5) | 0.0213 | 0.0122 | 0.0204 | 0.0263 |
|  | (1.35) | (0.79) | (1.15) | (1.63) |
|  |  |  |  |  |
| State (Reference: North Rhine-Westphalia) | | | | |
| Baden-Wuerttemberg | -0.00861 | 0.00361 | 0.00750 | -0.00944 |
|  | (-0.78) | (0.33) | (0.60) | (-0.83) |
|  |  |  |  |  |
| Bavaria | -0.0231 | 0.00717 | -0.00545 | 0.000771 |
|  | (-1.89) | (0.60) | (-0.40) | (0.06) |
|  |  |  |  |  |
| Berlin | 0.0226 | 0.0191 | -0.0108 | 0.0119 |
|  | (1.19) | (1.03) | (-0.50) | (0.61) |
|  |  |  |  |  |
| Brandenburg | -0.0445 | -0.0320 | -0.0372 | -0.0321 |
|  | (-1.78) | (-1.30) | (-1.32) | (-1.25) |
|  |  |  |  |  |
| Bremen | 0.00596 | 0.0174 | 0.00233 | 0.00312 |
|  | (0.33) | (0.98) | (0.11) | (0.17) |
|  |  |  |  |  |
| Hamburg | -0.0230 | 0.0114 | 0.0155 | 0.0322 |
|  | (-0.88) | (0.44) | (0.53) | (1.20) |
|  |  |  |  |  |
| Hesse | 0.0123 | 0.0305 | 0.0145 | -0.0195 |
|  | (0.75) | (1.91) | (0.79) | (-1.17) |
|  |  |  |  |  |
| Mecklenburg-Vorpommern | 0.00278 | -0.00690 | -0.0333 | -0.00695 |
|  | (0.09) | (-0.24) | (-0.99) | (-0.23) |
|  |  |  |  |  |
| Lower Saxony | -0.00106 | 0.0193 | 0.0111 | 0.000654 |
|  | (-0.07) | (1.38) | (0.69) | (0.04) |
|  |  |  |  |  |
| Rhineland Palatinate | -0.0318 | -0.00439 | -0.0357 | -0.0483^*^ |
|  | (-1.65) | (-0.23) | (-1.65) | (-2.45) |
|  |  |  |  |  |
| Saarland | 0.00719 | 0.0230 | 0.0278 | 0.0315 |
|  | (0.19) | (0.63) | (0.66) | (0.82) |
|  |  |  |  |  |
| Saxony | 0.0321 | 0.0297 | 0.0120 | 0.0288 |
|  | (1.59) | (1.50) | (0.53) | (1.39) |
|  |  |  |  |  |
| Saxony-Anhalt | -0.0501 | 0.0166 | -0.0754^*^ | -0.0296 |
|  | (-1.58) | (0.54) | (-2.12) | (-0.91) |
|  |  |  |  |  |
| Schleswig Holstein | -0.0148 | 0.0102 | 0.0274 | 0.0218 |
|  | (-0.70) | (0.49) | (1.15) | (1.01) |
|  |  |  |  |  |
| Thuringia | 0.00335 | 0.0271 | 0.0253 | 0.0473 |
|  | (0.14) | (1.14) | (0.92) | (1.90) |
|  |  |  |  |  |
| Foreign country | -0.0183 | -0.0181 | -0.0423 | -0.0410 |
|  | (-0.56) | (-0.56) | (-1.14) | (-1.22) |
|  |  |  |  |  |
| Gender (Reference: male) | | | | |
| female | 0.0319^***^ | -0.00838 | 0.00367 | 0.0225^**^ |
|  | (4.01) | (-1.08) | (0.41) | (2.77) |
|  |  |  |  |  |
| non-binary | 0.0296 | 0.0315 | 0.0129 | 0.00357 |
|  | (0.66) | (0.72) | (0.26) | (0.08) |
|  |  |  |  |  |
| Age group (Reference: 18-30) | | | | |
| 31-45 | -0.0706^***^ | -0.0660^***^ | -0.0557^***^ | -0.0688^***^ |
|  | (-6.49) | (-6.20) | (-4.55) | (-6.18) |
|  |  |  |  |  |
| 46-60 | -0.0823^***^ | -0.102^***^ | -0.0997^***^ | -0.112^***^ |
|  | (-7.37) | (-9.30) | (-7.92) | (-9.82) |
|  |  |  |  |  |
| > 60 | -0.124^***^ | -0.150^***^ | -0.145^***^ | -0.145^***^ |
|  | (-10.01) | (-12.40) | (-10.42) | (-11.39) |
|  |  |  |  |  |
| Educational attainment (Reference: low) | | | | |
| middle | -0.0127 | -0.00812 | -0.00764 | -0.0125 |
|  | (-0.64) | (-0.42) | (-0.34) | (-0.61) |
|  |  |  |  |  |
| high | 0.00804 | 0.0130 | 0.0227 | -0.00940 |
|  | (0.42) | (0.70) | (1.06) | (-0.48) |
|  |  |  |  |  |
| Household income (Reference: I find it very difficult to make ends meet) | | | | |
| I find it somewhat difficult to make ends meet | 0.0252 | 0.00610 | 0.0382 | 0.0120 |
|  | (0.95) | (0.24) | (1.28) | (0.44) |
|  |  |  |  |  |
| I can make ends meet | 0.0332 | 0.0103 | 0.0434 | -0.00889 |
|  | (1.36) | (0.43) | (1.58) | (-0.36) |
|  |  |  |  |  |
| I can live somewhat comfortably on the income | 0.0291 | 0.00664 | 0.0434 | -0.0163 |
|  | (1.19) | (0.28) | (1.58) | (-0.65) |
|  |  |  |  |  |
| I can live very comfortably on the income | 0.0478 | 0.0176 | 0.0443 | -0.0106 |
|  | (1.87) | (0.70) | (1.54) | (-0.41) |
|  |  |  |  |  |
| does not apply (no own household income) | 0.0107 | -0.00861 | 0.0241 | -0.0311 |
|  | (0.27) | (-0.22) | (0.55) | (-0.77) |
|  |  |  |  |  |
| Health status | 0.0550^**^ | 0.0438^*^ | 0.0353 | 0.0295 |
|  | (2.96) | (2.41) | (1.69) | (1.55) |
|  |  |  |  |  |
| Constant | 0.0222 | 0.170^**^ | 0.134^*^ | 0.258^***^ |
|  | (0.39) | (3.07) | (2.10) | (4.45) |
| Observations | 6680 | 6680 | 6680 | 6680 |
| *R*^2^ | 0.134 | 0.147 | 0.168 | 0.110 |
| Adjusted *R*^2^ | 0.125 | 0.139 | 0.159 | 0.101 |

*Note:* T statistics in parentheses. ^*^ *p* < 0.05, ^**^ *p* < 0.01, ^***^ *p* < 0.001; GAL, green/alternative/liberal; TAN, traditional/authoritarian/nationalist.

## Supplementary Table 6: Outcome II - Main model & cross check models, multiple OLS regression (b-coefficients)

| Outcome II: If money is paid for vaccination, how much is adequate? | Main model | Crosscheck concrete drivers for vaccination decision | Crosscheck self-perc eived knowledge of SARS-CoV-2 vaccination | Crosscheck private health insurance |
| --- | --- | --- | --- | --- |
| Frame (Reference: Control group) | | | | |
| Long COVID frame 1 | 2.099 | 3.167 | 2.598 | 2.020 |
|  | (0.65) | (0.96) | (0.76) | (0.62) |
|  |  |  |  |  |
| Long COVID frame 2 | 7.903^*^ | 10.08^**^ | 7.907^*^ | 7.874^*^ |
|  | (2.46) | (3.08) | (2.32) | (2.45) |
|  |  |  |  |  |
| Economic frame 1 | 4.356 | 5.073 | 4.997 | 4.319 |
|  | (1.36) | (1.55) | (1.47) | (1.34) |
|  |  |  |  |  |
| Economic frame 2 | 2.533 | 2.832 | 0.456 | 2.477 |
|  | (0.78) | (0.86) | (0.13) | (0.76) |
|  |  |  |  |  |
| Number of COVID-19 vaccinations received (Reference: not vaccinated) | | | | |
| one vaccination received | -4.453 | 0 | -0.749 | -4.166 |
|  | (-0.38) | (.) | (-0.06) | (-0.35) |
|  |  |  |  |  |
| two vaccinations received | 6.802 | 11.35 | 5.914 | 6.713 |
|  | (1.21) | (0.99) | (0.97) | (1.19) |
|  |  |  |  |  |
| three vaccinations received | 1.733 | 9.000 | 5.699 | 1.635 |
|  | (0.38) | (0.80) | (1.16) | (0.36) |
|  |  |  |  |  |
| four vaccinations received | 3.167 | 11.76 | 7.190 | 2.815 |
|  | (0.56) | (0.99) | (1.20) | (0.49) |
|  |  |  |  |  |
| Immunization decision was very difficult and emotionally highly stressful for me. | 17.14^***^ | 16.90^**^ | 13.89^**^ | 17.19^***^ |
|  | (3.47) | (2.94) | (2.67) | (3.47) |
|  |  |  |  |  |
| Immunization decision didn't take long for me. | -4.137 | 0.212 | -4.616 | -4.281 |
|  | (-1.00) | (0.05) | (-1.06) | (-1.03) |
|  |  |  |  |  |
| How threatening? COVID-19 | 6.566 | 9.881^*^ | 6.588 | 6.679 |
|  | (1.50) | (2.10) | (1.40) | (1.52) |
|  |  |  |  |  |
| Current COVID-19 safety perception | 10.73^*^ | 10.70^*^ | 7.408 | 11.03^*^ |
|  | (2.14) | (2.10) | (1.38) | (2.19) |
|  |  |  |  |  |
| Waldorf education | -12.01^**^ | -12.94^**^ | -10.41^*^ | -11.77^**^ |
|  | (-2.66) | (-2.81) | (-2.17) | (-2.61) |
|  |  |  |  |  |
| Homeopathy | -2.014 | -0.714 | -2.782 | -2.429 |
|  | (-0.49) | (-0.17) | (-0.64) | (-0.59) |
|  |  |  |  |  |
| Mainstream medicine | 1.353 | 0.659 | -1.827 | 1.292 |
|  | (0.23) | (0.10) | (-0.29) | (0.22) |
|  |  |  |  |  |
| Religious denomination (Reference: no denomination) | | | | |
| Roman-Catholic | 1.624 | 2.192 | 0.972 | 1.638 |
|  | (0.56) | (0.75) | (0.32) | (0.57) |
|  |  |  |  |  |
| Protestant | 1.151 | 1.571 | 1.979 | 1.192 |
|  | (0.39) | (0.53) | (0.64) | (0.40) |
|  |  |  |  |  |
| Evangelical Free Church | 5.001 | 3.611 | 6.520 | 5.057 |
|  | (1.30) | (0.92) | (1.59) | (1.32) |
|  |  |  |  |  |
| Orthodox Christian | -0.961 | 12.28 | -17.82 | -1.292 |
|  | (-0.05) | (0.53) | (-0.81) | (-0.06) |
|  |  |  |  |  |
| Jewish | 23.60 | 27.99 | -22.22 | 24.38 |
|  | (0.94) | (1.10) | (-0.81) | (0.97) |
|  |  |  |  |  |
| Muslim | 3.905 | 10.15 | 18.24 | 4.280 |
|  | (0.26) | (0.64) | (1.04) | (0.28) |
|  |  |  |  |  |
| Other | 3.633 | 9.275 | 0.248 | 3.543 |
|  | (0.49) | (1.20) | (0.03) | (0.48) |
|  |  |  |  |  |
| Left = 0/Right = 1 | 0.0113 | -3.671 | -6.423 | -1.039 |
|  | (0.00) | (-0.43) | (-0.74) | (-0.13) |
|  |  |  |  |  |
| GAL = 0/TAN = 1 | -1.918 | -0.354 | 2.587 | -1.857 |
|  | (-0.30) | (-0.05) | (0.38) | (-0.29) |
|  |  |  |  |  |
| Voting Intention (Reference: CDU/CSU: Christian democrats) | | | | |
| SPD (Social democrats) | 6.133 | 6.550 | 5.540 | 6.343 |
|  | (1.40) | (1.51) | (1.20) | (1.45) |
|  |  |  |  |  |
| Greens | 6.693 | 7.607^*^ | 5.380 | 7.017 |
|  | (1.74) | (1.97) | (1.33) | (1.82) |
|  |  |  |  |  |
| FDP (Liberals) | 6.033 | 4.417 | 4.539 | 6.038 |
|  | (1.33) | (0.97) | (0.93) | (1.33) |
|  |  |  |  |  |
| Left-Party (Socialist) | 6.806 | 6.916 | 4.377 | 7.164 |
|  | (1.22) | (1.22) | (0.74) | (1.29) |
|  |  |  |  |  |
| AfD (Populist right wing) | 14.73^**^ | 10.77 | 19.28^***^ | 15.20^**^ |
|  | (2.84) | (1.84) | (3.45) | (2.92) |
|  |  |  |  |  |
| Other | 0.897 | 2.261 | -0.974 | 1.341 |
|  | (0.22) | (0.53) | (-0.22) | (0.32) |
|  |  |  |  |  |
| Perception of social division | -10.14 | -7.961 | -13.24 | -10.10 |
|  | (-1.36) | (-1.04) | (-1.68) | (-1.35) |
|  |  |  |  |  |
| Solidarity | -15.32^*^ | -9.337 | -16.94^*^ | -15.34^*^ |
|  | (-2.08) | (-1.20) | (-2.15) | (-2.08) |
|  |  |  |  |  |
| Extraversion (Big 5) | -4.357 | -3.053 | -1.659 | -4.463 |
|  | (-0.98) | (-0.67) | (-0.35) | (-1.01) |
|  |  |  |  |  |
| Neuroticism (Big 5) | -0.802 | -1.100 | 1.614 | -0.896 |
|  | (-0.16) | (-0.21) | (0.30) | (-0.18) |
|  |  |  |  |  |
| Conscientiousness (Big 5) | -21.65^***^ | -18.02^**^ | -21.69^***^ | -21.64^***^ |
|  | (-3.85) | (-3.12) | (-3.63) | (-3.84) |
|  |  |  |  |  |
| Compatibility (Big 5) | -5.533 | -4.716 | -3.039 | -5.740 |
|  | (-0.96) | (-0.80) | (-0.49) | (-0.99) |
|  |  |  |  |  |
| Openness (Big 5) | 5.211 | 3.415 | 4.643 | 5.449 |
|  | (1.13) | (0.73) | (0.94) | (1.18) |
|  |  |  |  |  |
| State (Reference: North Rhine-Westphalia) | | | | |
| Baden-Wuerttemberg | 1.200 | 0.942 | 1.483 | 0.967 |
|  | (0.37) | (0.29) | (0.43) | (0.30) |
|  |  |  |  |  |
| Bavaria | 6.737 | 7.127 | 4.111 | 6.679 |
|  | (1.88) | (1.95) | (1.08) | (1.86) |
|  |  |  |  |  |
| Berlin | -0.131 | 0.0938 | 1.724 | -0.0511 |
|  | (-0.02) | (0.02) | (0.29) | (-0.01) |
|  |  |  |  |  |
| Brandenburg | 1.258 | 2.729 | 5.272 | 1.280 |
|  | (0.17) | (0.37) | (0.67) | (0.17) |
|  |  |  |  |  |
| Bremen | -2.027 | -2.039 | -1.732 | -1.758 |
|  | (-0.38) | (-0.38) | (-0.32) | (-0.33) |
|  |  |  |  |  |
| Hamburg | 12.77 | 13.61 | 12.19 | 12.82 |
|  | (1.66) | (1.77) | (1.45) | (1.67) |
|  |  |  |  |  |
| Hesse | -4.416 | -6.804 | -4.323 | -4.341 |
|  | (-0.92) | (-1.40) | (-0.85) | (-0.91) |
|  |  |  |  |  |
| Mecklenburg-Vorpommern | 11.43 | 6.300 | 11.83 | 11.29 |
|  | (1.31) | (0.68) | (1.27) | (1.29) |
|  |  |  |  |  |
| Lower Saxony | 3.664 | 2.802 | 2.163 | 3.720 |
|  | (0.88) | (0.66) | (0.49) | (0.89) |
|  |  |  |  |  |
| Rhineland Palatinate | 0.582 | -5.995 | -0.935 | 0.513 |
|  | (0.10) | (-1.05) | (-0.16) | (0.09) |
|  |  |  |  |  |
| Saarland | 3.777 | 6.304 | 9.951 | 3.759 |
|  | (0.34) | (0.56) | (0.85) | (0.34) |
|  |  |  |  |  |
| Saxony | 10.34 | 2.549 | 8.479 | 10.63 |
|  | (1.74) | (0.40) | (1.34) | (1.79) |
|  |  |  |  |  |
| Saxony-Anhalt | 0.225 | -0.585 | 6.537 | 0.850 |
|  | (0.02) | (-0.06) | (0.62) | (0.09) |
|  |  |  |  |  |
| Schleswig Holstein | 4.920 | 4.231 | 1.267 | 4.756 |
|  | (0.80) | (0.68) | (0.20) | (0.77) |
|  |  |  |  |  |
| Thuringia | 6.495 | 9.724 | 8.210 | 6.713 |
|  | (0.91) | (1.32) | (1.09) | (0.94) |
|  |  |  |  |  |
| Foreign country | -5.523 | -5.053 | -2.887 | -6.382 |
|  | (-0.57) | (-0.50) | (-0.28) | (-0.66) |
|  |  |  |  |  |
| Gender (Reference: male) |  |  |  |  |
| female | 0.336 | 0.950 | 0.674 | 0.534 |
|  | (0.14) | (0.40) | (0.28) | (0.23) |
|  |  |  |  |  |
| non-binary | 3.549 | 0.685 | 5.744 | 3.973 |
|  | (0.27) | (0.05) | (0.41) | (0.30) |
|  |  |  |  |  |
| Age group (Reference: 18-30) | | | | |
| 31-45 | -5.569 | -3.538 | -3.862 | -5.595 |
|  | (-1.75) | (-1.10) | (-1.11) | (-1.75) |
|  |  |  |  |  |
| 46-60 | -17.72^***^ | -16.82^***^ | -15.69^***^ | -18.09^***^ |
|  | (-5.41) | (-5.03) | (-4.42) | (-5.51) |
|  |  |  |  |  |
| > 60 | -25.17^***^ | -23.60^***^ | -24.20^***^ | -26.00^***^ |
|  | (-6.97) | (-6.43) | (-6.26) | (-7.15) |
|  |  |  |  |  |
| Educational attainment (Reference: low) | | | | |
| middle | -5.756 | 0.722 | -6.853 | -6.029 |
|  | (-0.99) | (0.12) | (-1.11) | (-1.04) |
|  |  |  |  |  |
| high | -12.03^*^ | -3.574 | -11.38 | -12.92^*^ |
|  | (-2.17) | (-0.63) | (-1.93) | (-2.32) |
|  |  |  |  |  |
| Household income (Reference: I find it very difficult to make ends meet) | | | | |
| I find it somewhat difficult to make ends meet | -10.51 | -11.26 | -14.29 | -10.57 |
|  | (-1.36) | (-1.34) | (-1.67) | (-1.36) |
|  |  |  |  |  |
| I can make ends meet | -13.36 | -13.68 | -20.32^*^ | -13.55 |
|  | (-1.88) | (-1.76) | (-2.56) | (-1.90) |
|  |  |  |  |  |
| I can live somewhat comfortably on the income | -17.34^*^ | -17.42^*^ | -22.38^**^ | -17.93^*^ |
|  | (-2.43) | (-2.25) | (-2.82) | (-2.50) |
|  |  |  |  |  |
| I can live very comfortably on the income | -16.75^*^ | -16.80^*^ | -23.88^**^ | -17.64^*^ |
|  | (-2.24) | (-2.09) | (-2.89) | (-2.35) |
|  |  |  |  |  |
| does not apply (no own household income) | -25.90^*^ | -25.32^*^ | -35.22^**^ | -26.56^*^ |
|  | (-2.25) | (-2.13) | (-2.72) | (-2.31) |
|  |  |  |  |  |
| Health status | 1.988 | 5.767 | 3.874 | 1.414 |
|  | (0.37) | (1.04) | (0.67) | (0.26) |
|  |  |  |  |  |
| Drivers of SARS-CoV-2 vaccination decision | | | | |
| Protecting self |  | -4.670 |  |  |
|  |  | (-0.86) |  |  |
|  |  |  |  |  |
| Protecting others |  | -10.39^*^ |  |  |
|  |  | (-2.15) |  |  |
|  |  |  |  |  |
| Participation in public events |  | 10.42^**^ |  |  |
|  |  | (3.03) |  |  |
|  |  |  |  |  |
| Vocational mandates |  | 3.903 |  |  |
|  |  | (1.26) |  |  |
|  |  |  |  |  |
| Medical advice/recommendation of the Permanent Vaccination Commission (STIKO) |  | -2.033 |  |  |
|  |  | (-0.53) |  |  |
|  |  |  |  |  |
| Peer pressure |  | -6.123 |  |  |
|  |  | (-1.52) |  |  |
|  |  |  |  |  |
| Self perception knowledge COVID-19 vaccination |  |  | 7.939 |  |
|  |  |  | (1.41) |  |
|  |  |  |  |  |
| Private health insurance |  |  |  | 4.794 |
|  |  |  |  | (1.91) |
|  |  |  |  |  |
| Constant | 79.24^***^ | 55.47^**^ | 80.18^***^ | 80.24^***^ |
|  | (4.79) | (2.78) | (4.36) | (4.84) |
| Observations | 6685 | 6028 | 5764 | 6680 |
| R2 | 0.038 | 0.041 | 0.037 | 0.038 |
| Adjusted R2 | 0.028 | 0.029 | 0.026 | 0.028 |

*Note*: T statistics in parentheses. * p < 0.05, ** p < 0.01, *** p < 0.001; GAL, green/alternative/liberal; TAN, traditional/authoritarian/nationalist.

## Supplementary Table 7: Survey Questions

| Variables | *Exact question (German original)* | | |  | *Exact question (English translation)* | | |
| --- | --- | --- | --- | --- | --- | --- | --- |
|  |  | | |  |  | | |
| Dependent variables |  | | |  |  | | |
| *Outcome I* |  | | |  |  | | |
| Meaningfulness of vaccine incentives (scale: 0-1) | Wie beurteilen Sie die folgenden Anreize, um Menschen zu einer Corona-Impfung zu bewegen?   - Freikarte für Fußballspiel - Gratis Grillwurst - Impflotterie (Chance auf großen Gewinn) - Geld gegen Impfung   Level:   - 0 = Gar nicht sinnvoll - 0.25 = Eher nicht sinnvoll - 0.5 = Neutrall - 0.75 = Eher sinnvoll - 1.0 = Sehr sinnvoll | | |  | How would you rate the following incentives to encourage people to have a Corona vaccination?   - Free ticket for football match - Free grilled sausage - Vaccination lottery (chance to win big) - Money for vaccination   Level:   - 0 = Not at all meaningful - 0.25 = Rather not meaningful - 0.5 = Neutral - 0.75 = Rather meaningful - 1.0 = Very meaningful | | |
| *Outcome II* |  | | |  |  | | |
| If money is paid for vaccination, how much is adequate? | Sofern es einen finanziellen Anreiz für eine Corona-Impfung geben würde, wieviel Geld hielten Sie für angemessen?   - Gar nicht, ich halte das Konzept finanzieller Anreize für falsch. - Bis zu 19,99 Euro - 20 bis 49,99 Euro - 50 bis 199,99 Euro - 200 bis 499,99 Euro - 500 Euro und mehr | | |  | Provided there was a financial incentive for Corona vaccination, how much money would you consider appropriate?   - Not at all, I think the concept of financial incentives is wrong. - Up to 19.99 EUR - 20 to 49.99 EUR - 50 to 199.99 EUR - 200 to 499.99 EUR - 500 EUR or more | | |
| Independent variable |  | | |  |  | | |
| Frame (control group; Long COVID 1 & 2, Economic 1 & 2) | Siehe “Note A1: Frames” (S. 2) | | |  | See "Note A1: Frames" (p. 2) | | |
| Controls: COVID-19 vaccination & perception of situation |  | | |  |  | | |
| # SARS-CoV-2 vaccine doses (scale: 0-4) | Haben Sie sich gegen COVID-19 impfen lassen?   - 0 = Nein, ich bin nicht geimpft - 1 = Ja, Erstimpfung erhalten - 2 = Ja, zwei Impfungen erhalten - 3 = Ja, drei Impfungen erhalten - 4 = Ja, vier Impfungen erhalten | | |  | Have you been vaccinated against COVID-19?   - 0 = No, I have not been vaccinated. - 1 = Yes, I got one dose. - 2 = Yes, I got two doses. - 3 = Yes, I got three doses. - 4 = Yes, I got four doses. | | |
| Immunization decision difficult & stressful (scale: 0-1) | Wie empfanden Sie damals die Entscheidung, ob Sie sich impfen lassen sollen oder nicht?  Die Entscheidungsfindung war für mich sehr schwierig und emotional hoch belastend.   - 0 = Stimme gar nicht zu - 0.25 = Stimme eher nicht zu - 0.5 = Teils/teils - 0.75 = Stimme eher zu - 1.0 = Stimme voll und ganz zu | | |  | How did you feel at the time about the decision whether or not to get vaccinated?  The decision making was very difficult for me and emotionally highly stressful.   - 0 = Completely disagree - 0.25 = Rather disagree - 0.5 = Partly/partly - 0.75 = Rather agree - 1.0 = Completely agree | | |
| Immunization decision fast (scale: 0-1) | Wie empfanden Sie damals die Entscheidung, ob Sie sich impfen lassen sollen oder nicht?  Die Entscheidungsfindung dauerte bei mir nicht lange.   - 0 = Stimme gar nicht zu - 0.25 = Stimme eher nicht zu - 0.5 = Teils/teils - 0.75 = Stimme eher zu - 1.0 = Stimme voll und ganz zu | | |  | How did you feel at the time about the decision whether or not to get vaccinated?  The decision making did not last long for me.   - 0 = Completely disagree - 0.25 = Rather disagree - 0.5 = Partly/partly - 0.75 = Rather agree - 1.0 = Completely agree | | |
| Threat of COVID-19 crisis (scale: 0-1) | Die heutige Zeit ist von vielen Krisen geprägt. Für wie bedrohlich halten Sie die folgenden Krisen?   - 0 = Gar nicht bedrohlich - 0.25 = Etwas bedrohlich - 0.5 = Teils/teils - 0.75 = Bedrohlich - 1.0 = Sehr bedrohlich | | |  | Today is marked by many crises. How threatening do you consider the following crises?   - 0 = Not at all threatening - 0.25 = Somewhat threatening - 0.5 = Partly/partly - 0.75 = Threatening - 1.0 = Very threatening | | |
| Safety perception regarding COVID-19 (scale: 0-1) | Wie sicher fühlen Sie sich aktuell vor einer schweren COVID-19 Erkrankung?   - 0 = Sehr unsicher - 0.33 = Eher unsicher - 0.67 = Eher sicher - 1.0 = Sehr sicher | | |  | How safe do you currently feel from severe COVID-19 disease?   - 0 = Very unsafe - 0.33 = Rather unsafe - 0.67 = Rather safe - 1.0 = Very safe | | |
| Controls: attitudes towards esoteric and medical belief systems & religion |  | | |  |  | | |
| Waldorf education (scale: 0-1) | Wie stehen Sie zu den folgenden Dingen?   - Waldorfschulen   Level:   - 0 = Sehr negativ - 0.25 = Eher negativ - 0.5 = Neutral - 0.75 = Eher positiv - 1.0 = Sehr positiv | | |  | How do you feel about the items below?  - Waldorf schools  Level:   - 0 = Very negative - 0.25 = Rather negative - 0.50 = Neutral - 0.75 = Rather positive - 1.0 = Very positive | | |
| Homeopathy  (scale: 0-1) | Wie stehen Sie zu den folgenden Dingen?   - Homeopathie   Level:   - 0 = Sehr negativ - 0.25 = Eher negativ - 0.5 = Neutral - 0.75 = Eher positiv - 1.0 = Sehr positiv | | |  | How do you feel about the items below?  - Homeopathy  Level:   - 0 = Very negative - 0.25 = Rather negative - 0.50 = Neutral - 0.75 = Rather positive - 1.0 = Very positive | | |
| Mainstream medicine  (scale: 0-1) | Wie stehen Sie zu den folgenden Dingen?   - Schulmedizin   Level:   - 0 = Sehr negativ - 0.25 = Eher negativ - 0.5 = Neutral - 0.75 = Eher positiv - 1.0 = Sehr positiv | | |  | How do you feel about the items below?  - Mainstream medicine  Level:   - 0 = Very negative - 0.25 = Rather negative - 0.50 = Neutral - 0.75 = Rather positive - 1.0 = Very positive | | |
| Religious denomination | Welcher Religion gehören Sie an? | | |  | Which religious denomination do you belong to? | | |
|  | - Keiner, ich bin konfessionslos | | |  | - none, I am non-denominational | | |
|  | - Römisch-katholisch | | |  | - Roman-Catholic | | |
|  | - Protestantisch | | |  | - Protestant | | |
|  | - Evangelische/Evangelikale Freikirche | | |  | - Evangelical Free Church | | |
|  | - Orthodoxe Kirche | | |  | - Orthodox Christian | | |
|  | - Jüdisch | | |  | - Jewish | | |
|  | - Muslimisch | | |  | - Muslim | | |
|  | - Sonstige, und zwar … (Freitexteingabe) | | |  | - Other, und zwar … (free text entry) | | |
| Controls: Political and psychosocial |  | | |  |  | | |
| Political ideology (scale 0-1) | Wie würden Sie sich politisch verorten?  Klicken Sie die grau unterlegte Leiste an und schieben Sie dann den Punkt an die gewünschte Position (*Schieberegler basierend auf einer 11-Punkte Skala*) | | |  | How would you position yourself politically?  Click the gray bar and then move the point to the desired position (*slider based on an 11-point scale*) | | |
|  | 0 = links | - | 1 = rechts |  | 0 = left | - | 1 = right |
|  | 0 = ökologisch/alternativ | - | 1 = traditionell/konservativ |  | 0 = ecological/ alternative | - | 1 = traditional/conservative |
| Voting intention | Wenn am Sonntag Bundestagswahl wäre, welche Partei würden Sie mit Ihrer Zweitstimme wählen? | | |  | If there were a federal election on Sunday, which party would you vote for with your Second Vote?^[[1]](#footnote-1)^ | | |
|  | - CDU/CSU | | |  | - CDU/CSU^[[2]](#footnote-2)^ (Christian democrats) | | |
|  | - SPD | | |  | - SPD (Social democrats) | | |
|  | - Grüne | | |  | - Greens | | |
|  | - FDP | | |  | - FDP (Liberals) | | |
|  | - Linke | | |  | - Left-Party (Socialist) | | |
|  | - AfD | | |  | - AfD (Populist right wing) | | |
|  | - Andere, und zwar … (Freitexteingabe) | | |  | - Others, namely … (Free text entry) | | |
| Perception of social division  *The perception of social division score is the arithmetic mean of a battery of five questions on the perception of divisions regarding potential societal cleavages.*  (scale: 0-1) | Für wie gespalten halten Sie die Gesellschaft in Bezug auf die folgenden Bereiche?   - Politische Fragen allgemein - Einkommens- und Vermögensverteilung - Ost- und Westdeutschland - Ökologie und Umweltthemen - Gendern   Level:   - Gar nicht gespalten = 0 - Ein wenig gespalten = 0.25 - Mittelmäßig gespalten = 0.5 - Ziemlich stark gespalten = 0.75 - Sehr stark gespalten = 1 | | |  | How divided do you think society is with regard to the following issues?   - Political questions in general - Distribution of income and wealth - East and West Germany - Ecological and environmental issues - The use of gendered language   Level:   - Not at all divided = 0 - A bit divided = 0.25 - Medium divided = 0.5 - Rather strongly divided = 0.75 - Very strongly divided = 1 | | |
| Solidarity  *The solidarity score is the arithmetic mean of a battery of four questions on attitudes towards social and personal solidarity* (scale: 0-1) | Nun möchten wir Ihnen ein paar Fragen zu Ihren Einstellungen stellen. Wie stehen Sie zu den folgenden Aussagen?   - Wer viel Steuern zahlt, tut damit schon genug für die Gesellschaft. - Die Gesellschaft ist auf freiwilliges Engagement der Bevölkerung angewiesen. - Es ist wichtiger, für das Wohl der Gemeinschaft zu arbeiten, als für das eigene Wohl. - Wenn ein Fremder auf meine Unterstützung angewiesen ist, versuche ich, sie ihm zu geben.   Level:   - 0 = Stimme überhaupt nicht zu - 0.25 = Stimme eher nicht zu - 0.5 = Neutral - 0.75 = Stimme eher zu - 1 = Stimme vollkommen zu | | |  | Now we would like to ask you a few questions about your attitudes. How do you feel about the following statements?   - Those who pay a lot of taxes are already doing enough for society. (*reversed for the calculation of the solidarity score)* - Society depends on the voluntary commitment of the population. - It is more important to work for the good of the community than for your own good. - If a stranger is in need of my support, I try to give it to them.   Level:   - Completely disagree = 0 - Rather disagree = 0.25 - Neutral = 0.5 - Rather agree = 0.75 - Completely agree = 1 | | |
| Big 5  *Personality is measured according to the five-factor model using the validated Big Five Inventory (BFI 10) developed by Rammstedt et al.*(1)*. The Big Five Dimensions scores are the arithmetic mean of each of two personality questions, one of which is formulated negatively and is therefore included in the calculation in reverse order.* (scale 0-1) | An dieser Stelle geht es um unterschiedliche Eigenschaften, die eine Person haben kann. Inwiefern treffen die folgenden Aussagen auf Sie zu?  Level:   - 0 = Trifft überhaupt nicht zu - 0.25 = Trifft eher nicht zu - 0.5 = Trifft mittelmäßig zu - 0.75 = Trifft eher zu - 1 = Trifft voll und ganz zu | | |  | At this point, we are talking about different characteristics that a person can have. To what extent do the following statements apply to you?  Level:   - 0 = Not true at all - 0.25 = Rather not true - 0.5 = Medium true - 0.75 = Rather true - 1 = Completely true | | |
| Extraversion (Big 5) | - Ich bin eher zurückhaltend, reserviert. - Ich gehe aus mir heraus, bin gesellig. | | |  | - I see myself as someone who is reserved (*reversed for the calculation*). - I see myself as someone who is outgoing, sociable. | | |
| Neuroticism (Big 5) | - Ich bin entspannt, lasse mich durch Stress nicht aus der Ruhe bringen. - Ich werde leicht nervös und unsicher. | | |  | - I see myself as someone who is relaxed, handles stress well (*reversed for the calculation*). - I see myself as someone who gets nervous easily. | | |
| Conscientiousness (Big 5) | - Ich bin bequem, neige zur Faulheit. - Ich erledige Aufgaben gründlich. | | |  | - I see myself as someone who tends to be lazy (*reversed for the calculation*). - I see myself as someone who does a thorough job. | | |
| Compatibility (Big 5) | - Ich schenke anderen leicht Vertrauen, glaube an das Gute im Menschen. - Ich neige dazu, andere zu kritisieren. | | |  | - I see myself as someone who is generally trusting. - I see myself as someone who tends to find fault with others (*reversed for the calculation*) | | |
| Openness (Big 5) | - Ich habe nur wenig künstlerisches Interesse. - Ich habe eine aktive Vorstellungskraft, bin fantasievoll. | | |  | - I see myself as someone who has few artistic interests (*reversed for the calculation*). - I see myself as someone who has an active imagination. | | |
| Controls: Socio-demographic |  | | |  |  | | |
| State (Bundesland) | In welchem Bundesland leben Sie? | | |  | In which state do you live? | | |
|  | - Baden-Württemberg | | |  | - Baden-Wuerttemberg | | |
|  | - Bayern | | |  | - Bavaria | | |
|  | - Berlin | | |  | - Berlin | | |
|  | - Brandenburg | | |  | - Brandenburg | | |
|  | - Bremen | | |  | - Bremen | | |
|  | - Hamburg | | |  | - Hamburg | | |
|  | - Hessen | | |  | - Hesse | | |
|  | - Mecklenburg-Vorpommern | | |  | - Mecklenburg-Vorpommern | | |
|  | - Niedersachsen | | |  | - Lower Saxony | | |
|  | - Nordrhein-Westfalen | | |  | - North Rhine-Westphalia | | |
|  | - Rheinland-Pfalz | | |  | - Rhineland Palatinate | | |
|  | - Saarland | | |  | - Saarland | | |
|  | - Sachsen | | |  | - Saxony | | |
|  | - Sachsen-Anhalt | | |  | - Saxony-Anhalt | | |
|  | - Schleswig-Holstein | | |  | - Schleswig Holstein | | |
|  | - Thüringen | | |  | - Thuringia | | |
|  | - Ich lebe im Ausland, und zwar … (Freitexteingabe) | | |  | - Foreign country | | |
| Gender | Welches Geschlecht haben Sie? | | |  | What is your gender? | | |
|  | - Männlich | | |  | - Male | | |
|  | - Weiblich | | |  | - Female | | |
|  | - Nicht binär | | |  | - Non-binary | | |
| Age group | Wie alt sind Sie? | | |  | How old are you? | | |
| 18-30  31-45  46-60  > 60 | - 12 – 99 Jahre | | |  | - 12 – 99 years  (*the answers were grouped into the 4 age brackets, participants younger than 18 years were excluded from the analysis*) | | |
| Educational attainment | Was ist Ihr höchster allgemeinbildender Schulabschluss? | | |  | What is the highest level of general education you have completed? | | |
| Low | - Kein Schulabschluss - Volks-/ Hauptschul-abschluss bzw. Poly-technische Oberschule mit Abschluss 8. oder 9. Klasse - Sonstiges | | |  | - No school-leaving certificate - Secondary school diploma or polytechnic high school diploma with completion of 8th or 9th grade - Other | | |
| Middle | - Mittlere Reife, Realschulabschluss bzw. Polytechnische Oberschule mit Abschluss 10. Klasse | | |  | - Secondary school diploma or polytechnic high school diploma with completion of 10th grade | | |
| High | - Fachabitur - Abitur bzw. Erweiterte Oberschule mit Abschluss 12. Klasse (Hochschulreife) - Noch in der Schule | | |  | - Vocational high school diploma - High school diploma or extended high school with completion of grade 12 (university entrance qualification) - Still at school | | |
| Household income | Wie beurteilen Sie Ihr gegenwärtiges Haushaltseinkommen? Mit meinem/unserem Einkommen kann ich/können wir...  Level: | | |  | How would you rate your current household income? With my/our income I/we can...  Level: | | |
|  | - Eigentlich gar nicht zurechtkommen | | |  | - I find it very difficult to make ends meet | | |
|  | - Nur schwer zurechtkommen | | |  | - I find it quite difficult to make ends meet | | |
|  | - Zurechtkommen | | |  | - I can make ends meet | | |
|  | - Bequem leben | | |  | - I can live quite comfortably on the income | | |
|  | - Sehr bequem leben | | |  | - I can live very comfortably on the income | | |
|  | - Trifft nicht zu (noch kein eigenes Haushaltseinkommen) | | |  | - Does not apply (no own household income) | | |
| Health status  (scale: 0-1) | Wie schätzen Sie – alles in allem – Ihren Gesundheitszustand ein? Würden Sie sagen er ist… | | |  | All in all, how would you rate your state of health? Would you say it is... | | |
|  | - 0 = Sehr schlecht | | |  | - 0 = Very poor | | |
|  | - 0.25 = Schlecht | | |  | - 0.25 = Poor | | |
|  | - 0.5 = Durchschnittlich | | |  | - 0.5 = Average | | |
|  | - 0.75 = Gut | | |  | - 0.75 = Good | | |
|  | - 1 = Sehr gut | | |  | - 1 = Very good | | |
| Controls: additional controls for supplementary cross checks |  | | |  |  | | |
| Self-perceived knowledge of COVID-19 immunization compared to others | Für wie gut informiert in Sachen Corona-Impfung halten Sie sich im Vergleich zu Ihren Gesprächspartnern? | | |  | How well informed do you consider yourself to be on the subject of Corona vaccination compared to your interlocutors? | | |
|  | - 0 = Sehr viel schlechter informiert | | |  | - 0 = Much less informed | | |
|  | - 0.25 = Etwas schlechter informiert | | |  | - 0.25 = Slightly less informed | | |
|  | - 0.5 = Etwa gleichgut informiert | | |  | - 0.5 = About equally well informed | | |
|  | - 0.75 = Etwas besser informiert | | |  | - 0.75 = Slightly better informed | | |
|  | - 1 = Sehr viel besser informiert | | |  | - 1 = Much better informed | | |
| Private health insurance | Wie sind Sie krankenversichert? | | |  | How are you covered by health insurance? | | |
|  | - 0 = Gesetzlich | | |  | - 0 = Statutory health insurance | | |
|  | - 1 = Privat | | |  | - 1 = Private health insurance | | |
| Concrete drivers of SARS-CoV-2 vaccination decision (This question was only asked to those who had received at least one dose of SARS-CoV-2 vaccine; scale: 0-1) | Wie wichtig waren die folgenden Punkte für Ihre Impfentscheidung? | | |  | How important were the following factors for your decision to get vaccinated? | | |
|  | - Mich selbst vor COVID-19 zu schützen | | |  | - Protecting myself from COVID-19 | | |
|  | - Andere vor COVID-19 zu schützen | | |  | - Protecting others from COVID-19 | | |
|  | - Unproblematische Teilnahme am öffentlichen Geschehen | | |  | - Participation in public events | | |
|  | - Notwendigkeit der Impfung bei meiner Arbeit | | |  | - Vocational mandate to get vaccinated | | |
|  | - ärztlicher Ratschlag bzw. allgemeine Impfempfehlung der Ständigen Impfkommission (STIKO) | | |  | - Medical advice/recommendation of the Permanent Vaccination Commission (STIKO) | | |
|  | - mein Bekannten- und Freundeskreis erwartete es | | |  | - Peer pressure | | |
|  | Level:   - 0 = Sehr unwichtig - 0.25 = Eher unwichtig - 0.5 = Mittel wichtig - 0.75 = Eher wichtig - 1 = Sehr wichtig | | |  | Level:   - 0 = Highly unimportant - 0.25 = Mostly unimportant - 0.5 = Semi-important - 0.75 = Mostly important - 1 = Highly important | | |

## Supplementary Table 8: Missing values – item non-response

|  | Missings | |
| --- | --- | --- |
|  | # | % |
| Dependent variables |  |  |
| *Outcome I* |  |  |
| Meaningfulness of vaccine incentives |  |  |
| Free ticket for football match | 42 | 0.53 |
| Free grilled sausage | 44 | 0.55 |
| Vaccination lottery (chance to win big) | 29 | 0.37 |
| Money | 28 | 0.35 |
| *Outcome II* |  |  |
| If money is paid for vaccination, how much is adequate? | 28 | 0.35 |
| Frame (control group; Long COVID 1 & 2, Economic 1 & 2) | 0 | 0 |
| Controls: COVID-19 vaccination & perception of situation |  |  |
| # SARS-CoV-2 vaccine doses | 14 | 0.18 |
| Immunization decision difficult & stressful | 217 | 2.73 |
| Immunization decision fast | 46 | 0.58 |
| Threat of COVID-19 crisis | 24 | 0.30 |
| Safety perception regarding COVID-19 | 9 | 0.11 |
| Controls: attitudes towards esoteric and medical belief systems & religion |  |  |
| Attitudes towards… |  |  |
| Waldorf education | 27 | 0.34 |
| Homeopathy | 13 | 0.16 |
| Mainstream medicine | 16 | 0.20 |
| Religious denomination | 56 | 0.70 |
| Controls: Political & psychosocial |  |  |
| Political ideology |  |  |
| Left - Right | 163 | 2.05 |
| Green/Alternative/Liberal (GAL) - Traditional/Authoritarian/Nationalist (TAN) | 272 | 3.42 |
| Voting intention | 60 | 0.76 |
| Perception of social division | 363 | 4.57 |
| Solidarity | 27 | 0.34 |
| Extraversion (Big 5) | 40 | 0.50 |
| Neuroticism (Big 5) | 29 | 0.37 |
| Conscientiousness (Big 5) | 33 | 0.42 |
| Compatibility (Big 5) | 37 | 0.47 |
| Openness (Big 5) | 36 | 0.45 |
| Controls: Socio-demographic |  |  |
| State (Bundesland) | 52 | 0.65 |
| Gender | 84 | 1.06 |
| Age* | 42 | 0.53 |
| Educational attainment | 44 | 0.55 |
| Household income | 55 | 0.69 |
| Health status | 49 | 0.62 |
| Controls: additional controls for supplementary cross checks |  |  |
| Self-perceived knowledge of COVID-19 immunization compared to others | 1,132 | 14.25 |
| Private health insurance | 56 | 0.70 |
| Concrete drivers of SARS-CoV-2 vaccination decision  (This question was only asked to those who had received at least one dose of SARS-CoV-2 vaccine, which results in higher numbers of missings) |  |  |
| Protecting self | 717 | 9.03 |
| Protecting others | 731 | 9.20 |
| Participation in public events | 726 | 9.14 |
| Vocational mandates | 789 | 9.93 |
| Medical advice/recommendation of the Permanent Vaccination Commission (STIKO) | 727 | 9.15 |
| Peer pressure | 749 | 9.43 |

*Note*: Total number of cases, i.e. participants who started the survey and at least reached the page with the frame: N = 7,944.* Additionally, a total of 28 participants was younger than 18. These participants were excluded from the analyses.

## Supplementary Table 9: Descriptive statistics for the original dataset and the final dataset used in the regression models (reduced via listwise deletion)

|  | Original data | | | Reduced datset for regression analysis (listwise deletion) | | |
| --- | --- | --- | --- | --- | --- | --- |
|  | N | Mean  (or % for categorical data) | Sd | N | Mean (or % for categorical data) | Sd |
| Dependent variables |  |  |  |  |  |  |
| *Outcome I* |  |  |  |  |  |  |
| Meaningfulness of vaccine incentives |  |  |  |  |  |  |
| Free ticket for football match | 7902 | 0.22 | 0.30 | 6685 | 0.22 | 0.30 |
| Free grilled sausage | 7900 | 0.20 | 0.30 | 6685 | 0.21 | 0.30 |
| Vaccination lottery (chance to win big) | 7915 | 0.30 | 0.35 | 6685 | 0.30 | 0.35 |
| Money | 7916 | 0.21 | 0.30 | 6685 | 0.21 | 0.31 |
| *Outcome II* |  |  |  |  |  |  |
| If money is paid for vaccination, how much is adequate? | 7916 | 26.28 | 83.56 | 6685 | 26.77 | 84.14 |
| Frame | 7944 | % |  | 6685 | % |  |
| Control group | 1564 | 19.69 |  | 1329 | 19.88 |  |
| Long COVID Frame 1 | 1580 | 19.89 |  | 1323 | 19.79 |  |
| Long COVID Frame 2 | 1597 | 20.10 |  | 1352 | 20.22 |  |
| Economic Frame 1 | 1612 | 20.29 |  | 1366 | 20.43 |  |
| Economic Frame 2 | 1591 | 20.03 |  | 1315 | 19.67 |  |
| Controls: COVID-19 vaccination & perception of situation |  |  |  |  |  |  |
| # SARS-CoV-2 vaccine doses | 7930 | 2.77 | 0.98 | 6685 | 2.77 | 0.97 |
| Immunization decision difficult & stressful | 7727 | 0.16 | 0.28 | 6685 | 0.15 | 0.28 |
| Immunization decision fast | 7898 | 0.82 | 0.32 | 6685 | 0.82 | 0.31 |
| Threat of COVID-19 crisis | 7920 | 0.44 | 0.28 | 6685 | 0.44 | 0.28 |
| Safety perception regarding COVID-19 | 7935 | 0.69 | 0.23 | 6685 | 0.69 | 0.23 |
| Controls: attitudes towards esoteric and medical belief systems & religion |  |  |  |  |  |  |
| Attitudes towards… |  |  |  |  |  |  |
| Waldorf education | 7917 | 0.40 | 0.25 | 6685 | 0.40 | 0.25 |
| Homeopathy | 7931 | 0.40 | 0.31 | 6685 | 0.38 | 0.31 |
| Mainstream medicine | 7928 | 0.77 | 0.20 | 6685 | 0.77 | 0.20 |
| Religious denomination | 7888 | % |  | 6685 | % |  |
| non-denominational | 4057 | 51.43 |  | 3447 | 51.56 |  |
| Roman-Catholic | 1556 | 19.73 |  | 1318 | 19.72 |  |
| Protestant | 1347 | 17.08 |  | 1133 | 16.95 |  |
| Evangelical Free Church | 688 | 8.72 |  | 596 | 8.92 |  |
| Orthodox | 24 | 0.30 |  | 17 | 0.25 |  |
| Jewish | 13 | 0.16 |  | 11 | 0.16 |  |
| Muslim | 36 | 0.46 |  | 30 | 0.45 |  |
| Other | 167 | 2.12 |  | 133 | 1.99 |  |
| Controls: Political & psychosocial |  |  |  |  |  |  |
| Political ideology |  |  |  |  |  |  |
| Left - Right | 7781 | 0.42 | 0.20 | 6685 | 0.41 | 0.20 |
| Green/Alternative/Liberal (GAL) - Traditional/Authoritarian/Nationalist (TAN) | 7672 | 0.45 | 0.24 | 6685 | 0.45 | 0.24 |
| Voting intention | 7884 | % |  | 6685 | % |  |
| CDU/CSU | 1164 | 14.76 |  | 962 | 14.39 |  |
| SPD | 948 | 12.02 |  | 803 | 12.01 |  |
| Greens | 2628 | 33.33 |  | 2290 | 34.26 |  |
| FDP | 648 | 8.22 |  | 558 | 8.35 |  |
| Left-Party | 558 | 7.08 |  | 473 | 7.08 |  |
| AfD | 607 | 7.70 |  | 505 | 7.55 |  |
| Others | 1331 | 16.88 |  | 1094 | 16.36 |  |
|  |  |  |  |  |  |  |
| Perception of social division | 7581 | 0.67 | 0.15 | 6685 | 0.67 | 0.14 |
| Solidarity | 7917 | 0.65 | 0.17 | 6685 | 0.66 | 0.17 |
| Extraversion (Big 5) | 7904 | 0.52 | 0.25 | 6685 | 0.52 | 0.25 |
| Neuroticism (Big 5) | 7915 | 0.42 | 0.22 | 6685 | 0.42 | 0.22 |
| Conscientiousness (Big 5) | 7911 | 0.67 | 0.20 | 6685 | 0.67 | 0.20 |
| Compatibility (Big 5) | 7907 | 0.53 | 0.19 | 6685 | 0.53 | 0.19 |
| Openness (Big 5) | 7908 | 0.62 | 0.23 | 6685 | 0.62 | 0.23 |
| Controls: Socio-demographic |  |  |  |  |  |  |
| State (Bundesland) | 7892 | % |  | 6685 | % |  |
| Baden-Wuerttemberg | 1664 | 21.08 |  | 1386 | 20.73 |  |
| Bavaria | 1085 | 13.75 |  | 931 | 13.93 |  |
| Berlin | 317 | 4.02 |  | 277 | 4.14 |  |
| Brandenburg | 169 | 2.14 |  | 145 | 2.17 |  |
| Bremen | 379 | 4.80 |  | 318 | 4.76 |  |
| Hamburg | 152 | 1.93 |  | 130 | 1.94 |  |
| Hesse | 465 | 5.89 |  | 398 | 5.95 |  |
| Mecklenburg-Vorpommern | 113 | 1.43 |  | 99 | 1.48 |  |
| Lower Saxony | 717 | 9.09 |  | 588 | 8.80 |  |
| North Rhine-Westphalia | 1518 | 19.23 |  | 1310 | 19.60 |  |
| Rhineland Palatinate | 319 | 4.04 |  | 263 | 3.93 |  |
| Saarland | 73 | 0.92 |  | 60 | 0.90 |  |
| Saxony | 288 | 3.65 |  | 244 | 3.65 |  |
| Saxony-Anhalt | 105 | 1.33 |  | 87 | 1.30 |  |
| Schleswig Holstein | 251 | 3.18 |  | 214 | 3.20 |  |
| Thuringia | 185 | 2.34 |  | 155 | 2.32 |  |
| Foreign country | 92 | 1.17 |  | 80 | 1.20 |  |
| Gender | 7860 | % |  | 6685 | % |  |
| male | 4901 | 62.35 |  | 4218 | 63.10 |  |
| female | 2914 | 37.07 |  | 2426 | 36.29 |  |
| non-binary | 45 | 0.57 |  | 41 | 0.61 |  |
| Age group | 7874 | % |  | 6685 | % |  |
| 18-30 | 1439 | 18.28 |  | 1311 | 19.61 |  |
| 31-45 | 1887 | 23.96 |  | 1706 | 25.52 |  |
| 46-60 | 2433 | 30.90 |  | 2073 | 31.01 |  |
| > 60 | 2115 | 26.86 |  | 1595 | 23.86 |  |
| Educational attainment | 7900 | % |  | 6685 | % |  |
| low | 338 | 4.28 |  | 250 | 3.74 |  |
| middle | 1607 | 20.34 |  | 1291 | 19.31 |  |
| high | 5955 | 75.38 |  | 5144 | 76.95 |  |
| Household income | 7889 | % |  | 6685 | % |  |
| I find it very difficult to make ends meet | 185 | 2.35 |  | 151 | 2.26 |  |
| I find it somewhat difficult to make ends meet | 603 | 7.64 |  | 509 | 7.61 |  |
| I can make ends meet | 2367 | 30.00 |  | 1979 | 29.60 |  |
| I can live somewhat comfortably on the income | 3256 | 41.27 |  | 2786 | 41.68 |  |
| I can live very comfortably on the income | 1375 | 17.43 |  | 1174 | 17.56 |  |
| does not apply (no own household income) | 103 | 1.31 |  | 86 | 1.29 |  |
| Health status | 7895 | 0.69 | 0.22 | 6685 | 0.70 | 0.21 |
| Controls: additional controls for supplementary cross checks |  |  |  |  |  |  |
| Self-perceived knowledge of COVID-19 immunization compared to others | 6812 | 0.70 | 0.20 | 5764 | 0.70 | 0.20 |
| Health insurance | 7888 | % |  | 6680 | % |  |
| Statutory health insurance | 5892 | 74.70 |  | 5016 | 75.09 |  |
| Private health insurance | 1996 | 25.30 |  | 1664 | 24.91 |  |
| Concrete drivers of SARS-CoV-2 vaccination decision |  |  |  |  |  |  |
| Protecting self | 7227 | 0.84 | 0.27 | 6106 | 0.84 | 0.27 |
| Protecting others | 7213 | 0.78 | 0.29 | 6098 | 0.78 | 0.29 |
| Participation in public events | 7218 | 0.58 | 0.33 | 6101 | 0.57 | 0.33 |
| Vocational mandates | 7155 | 0.37 | 0.37 | 6060 | 0.36 | 0.37 |
| Medical advice/recommendation of the Permanent Vaccination Commission (STIKO) | 7217 | 0.60 | 0.33 | 6101 | 0.60 | 0.32 |
| Peer pressure | 7195 | 0.23 | 0.28 | 6090 | 0.23 | 0.28 |

*Note*: The comparison between the original dataset (using all existing information from the survey) and the dataset reduced by listwise deletion (dropping all cases that have at least one missing value), the latter being the dataset used in the regression analyses, shows only marginal differences. The means and standard deviations as well as the percentage shares for the categorical variables are virtually identical. Therefore, item non-response does not introduce systematic bias and a complete cases regression analysis is appropriate.

## Supplementary Figure 1: Descriptive statistics of dependent variables by gender

*Note*: **Annotation: a** Percentages of survey participants’ attitudes towards four COVID-19 vaccination incentives by gender (non binary, N = 41, not presented). **b** Percentages of survey participants’ views on the appropriate level of a potential direct monetary compensation for COVID-19 vaccination by gender (non binary, N = 41, not presented). N = 6,644. Survey data from Politikpanel Deutschland, 06/30-07/17/2022 (<https://www.politikpanel.uni-freiburg.de/>).

## Supplementary Figure 2: Associations between frames and dependent variables – bivariate regression analyses, comparison between OLS and ordinal logit models (reference = control group; + 95% CI, full sample)

 *Note*: **a** & **c**: b-coefficients from bivariate OLS regression; **b** & **d**: Odds ratios from bivariate ordinal logistic regeression. N = 6,685. Survey data from Politikpanel Deutschland, 06/30-07/17/2022 (<https://www.politikpanel.uni-freiburg.de/>).

## Supplementary Figure 3: Associations between frames and dependent variables – multiple regression analyses, comparison between OLS and Ologit models (reference = control group; + 95% CI, full sample)

*Note*: **a** & **c**: b-coefficients from bivariate OLS regression; **b** & **d**: Odds ratios from bivariate ordinal logistic regeression. N = 6,685. Survey data from Politikpanel Deutschland, 06/30-07/17/2022 (<https://www.politikpanel.uni-freiburg.de/>). The b-coefficients for the OLS models are based on the main models for Outcome I and Outcome II (see Supplementary Tables 1 and 5). The coefficients for the control variables are omitted in the figure. The Ologit models are based on exactly the same cases and control for the same factors as the OLS models.

## Supplementary Figure 4: Predicted meaningfulness of vaccination incentives based on multiple OLS models with pooled frames

##

*Note*: N = 6,685. Survey data from Politikpanel Deutschland, 06/30-07/17/2022 (<https://www.politikpanel.uni-freiburg.de/>). Predictions + 95% CI based on multiple OLS models including controls.

## Supplementary Figure 5: Average importance of concrete drivers for SARS-CoV-2 vaccination

*Note*: **a**: means by sex & age group, **b**: means by voting intention. N = 6,685. Survey data from Politikpanel Deutschland, 06/30-07/17/2022 (<https://www.politikpanel.uni-freiburg.de/>).

## Supplementary Figure 6: Distribution of main demographic variables in the sample compared to reality

*Note*: Survey data (N = 6,685) from Politikpanel Deutschland, 06/30-07/17/2022 (<https://www.politikpanel.uni-freiburg.de/>). Official statistics from the Federal Statistical Office of Germany, Destatis (<https://www-genesis.destatis.de/genesis/online>). Opinion poll data is the mean of the Forschungsgruppe Wahlen, FORSA, Infratest Dimap, and YouGov polls (source: <https://www.wahlrecht.de/umfragen/>).

## Supplementary Figure 7: Money deemed acceptable as incentive for COVID-19 vaccination (pooled frames: a) Long COVID frames/Economic frames; b) all four frames pooled)

*Note***:** N = 6,685. Survey data from Politikpanel Deutschland, 06/30-07/17/2022 (<https://www.politikpanel.uni-freiburg.de/>). Predictions based on OLS regression + 95% CI. The model additionally controls for the full set of controls as in Figure 6 from the main article.

## Literature

1. B. Rammstedt, C. J. Kemper, M. C. Klein, C. Beierlein, A. Kovaleva, “Big Five Inventory (BFI-10)” (ZIS - GESIS Leibniz Institute for the Social Sciences, 2014).

1. In Germany voters have two votes for the election of the German parliament, the Bundestag. With the so called “First Vote” (*Erststimme*) voters determine a direct candidate of their constituency, with the “Second Vote” (*Zweitstimme*) electors vote for a party. This second vote determines the distribution of seats in the Bundestag and is therefore more important than the First Vote. [↑](#footnote-ref-1)
2. CDU (Christian Democratic Union) and CSU (Christian Social Union in Bavaria) are sister parties. Together they are called the Union. Both always form a single fraction in the Bundestag. While the CSU operates only in Bavaria, the CDU operates only in the 15 other German states. In the survey CDU and CSU were queried as separate items. In the analysis both were combined. [↑](#footnote-ref-2)
